# Separation and concentration of CO_2_ from air using a humidity-driven molten-carbonate membrane

**DOI:** 10.1038/s41560-024-01588-6

**Published:** 2024-07-19

**Authors:** Ian S. Metcalfe, Greg A. Mutch, Evangelos I. Papaioannou, Sotiria Tsochataridou, Dragos Neagu, Dan J. L. Brett, Francesco Iacoviello, Thomas S. Miller, Paul R. Shearing, Patricia A. Hunt

**Affiliations:** 1 Materials, Concepts & Reaction Engineering (MatCoRE) Group, School of Engineering, Newcastle University, Newcastle upon Tyne, UK; 2 Department of Chemical and Process Engineering, University of Strathclyde, Glasgow, UK; 3 Electrochemical Innovation Lab, Department of Chemical Engineering, University College London, London, UK; 4 The ZERO Institute, University of Oxford, Oxford, UK; 5 Department of Chemistry, Molecular Sciences Research Hub, Imperial College London, White City Campus, London, UK; 6 School of Chemical and Physical Sciences, Victoria University of Wellington, Wellington, New Zealand

**Keywords:** Chemical engineering, Materials for devices, Carbon capture and storage, Materials for energy and catalysis

## Abstract

Separation processes are substantially more difficult when the species to be separated is highly dilute. To perform any dilute separation, thermodynamic and kinetic limitations must be overcome. Here we report a molten-carbonate membrane that can ‘pump’ CO_2_ from a 400 ppm input stream (representative of air) to an output stream with a higher concentration of CO_2_, by exploiting ambient energy in the form of a humidity difference. The substantial H_2_O concentration difference across the membrane drives CO_2_ permeation ‘uphill’ against its own concentration difference, analogous to active transport in biological membranes. The introduction of this H_2_O concentration difference also results in a kinetic enhancement that boosts the CO_2_ flux by an order of magnitude even as the CO_2_ input stream concentration is decreased by three orders of magnitude from 50% to 400 ppm. Computational modelling shows that this enhancement is due to the H_2_O-mediated formation of carriers within the molten salt that facilitate rapid CO_2_ transport.

## Abstract (web summary)

Capture of CO_2_ from the air requires substantial amounts of energy. Here the authors report molten-carbonate membranes to concentrate CO_2_ from 400 ppm input streams that exploit ambient energy in the form of humidity differences.

## Main

Separation processes are ubiquitous. They are essential for achieving product quality and minimizing waste in a wide range of industries spanning energy, chemicals, water, food and medicines. Dilute separations are the most challenging, due to two important problems. First, thermodynamic work (an energy input) is required to raise the concentration of the species from the dilute input stream to that of a more concentrated output stream. Second, as the input stream is dilute, the kinetics of any separation process tend to be slow.

There are many important dilute separations. For example, a process for the efficient separation of CO_2_ from air is urgently required, having been identified as one of the ‘chemical separations to change the world’^1^. For CO_2_ in the air to be stored permanently underground or used as a chemical feedstock, it must first be separated from air at ~400 ppm and concentrated (a thermodynamically ‘uphill’ process). Progress has been made by developing sorbent-based processes^2,3^, but the considerable volume of sorbent material required in large air capture devices dominates capital costs^4–6^. Membranes offer an elegant alternative where the best separation performance is achieved with thin membranes, leading to reduced materials requirements and the potential for much lower capital costs. However, at low concentrations of CO_2_, even the most advanced synthetic membranes struggle to achieve reasonable permeation rates^2,7^. Furthermore, synthetic membranes with higher permeability typically have lower selectivity^8^.

**Fig. 1:**
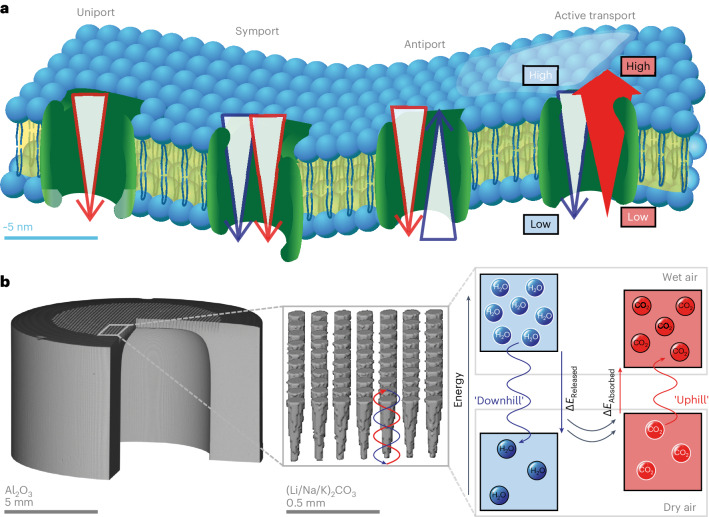
Transport in biological membranes and the humidity-driven synthetic membrane. **a**, In biological membranes, transport is typically passive, down a concentration gradient (uniport, symport and antiport); however, in active transport, uphill transport against a concentration gradient for one species (red) can be achieved via an intimate coupling with downhill transport of a second species (blue). **b**, A three-dimensional reconstruction of micro-computed X-ray tomography scans of a synthetic, supported molten-salt membrane, comprising an alumina (Al_2_O_3_) support with a ternary eutectic mixture of molten carbonates ((Li/Na/K)_2_CO_3_) held in laser-drilled artificial pores. A humidity difference (wet air and dry air) is harnessed to pump CO_2_ from one air stream to the other, against its concentration gradient, to produce a CO_2_-enriched output stream.

Biological membranes exploit selective, high-mobility carriers, that is, the transport of a target species is facilitated, leading to ultrahigh permeability. Such transport does not normally require an energy input as it is down a concentration gradient (from high to low concentration). However, during active transport, biological membranes can harness the energy released during ‘downhill’ transport of a first species to ‘pump’ a second species to higher concentration (Fig. 1a)^9^. This is an exercise in energy balancing; energy released in one process is used to drive a process requiring an energy input. Such a mechanism could be advantageous for CO_2_ capture from air where the costs associated with providing typical energy inputs, for example, heat or pressure, are likely to prove intolerable at scale^5^.

Here we report a synthetic, CO_2_-permeable, molten-carbonate membrane that has been designed to hijack a humidity difference to pump CO_2_ uphill from a dry air input stream into a wet air output stream (Fig. 1b). Air must be used for both streams as the use of any other gas (for example, an inert gas or pure H_2_O) would render the process too costly. To avoid external energy input, the two air streams are at the same temperature and pressure, and the humidity difference between the streams is exploited as an internal energy input (a humidity difference was chosen as H_2_O is the only species in air with a concentration that varies to any meaningful extent). We find that the introduction of H_2_O also increases CO_2_ flux by an order of magnitude (with no loss of CO_2_ selectivity) even as the input stream CO_2_ concentration is decreased by three orders of magnitude from 50% to 400 ppm. This intimate coupling of H_2_O and CO_2_ permeation tackles both the thermodynamic and kinetic components of this dilute separation.

## Membrane design and characterization

For the membrane to function as described above, incorporation, transport through the membrane, and release of both H_2_O and CO_2_ is required. In molten salts, such processes are typically mediated by high-mobility ionic species that act as ‘carriers’^10^. If the incorporation reactions of H_2_O and CO_2_ with generic carriers X and Y are thermodynamically favourable, then the equilibrium position of both reactions 1 and 2 will lie to the right, where the carriers are fully loaded.

Reaction 1 $${{\rm{H}}}_{2}{\rm{O}}+{\rm{X}}\rightleftarrows {\rm{X}}{\rm{\cdot }}{{\rm{H}}}_{2}{\rm{O}}$$

Reaction 2 $${\rm{C}}{{\rm{O}}}_{2}+{\rm{Y}}\rightleftarrows {\rm{Y}}{\rm{\cdot }}{\rm{C}}{{\rm{O}}}_{2}$$

However, the release reactions (reverse of reactions 1 and 2) will then tend to be unfavourable. Thus, CO_2_ is required to facilitate the release of H_2_O from X·H_2_O and equally H_2_O to facilitate the release of CO_2_ from Y·CO_2_. This can occur if X and Y represent a common carrier (now denoted Z), leading to the overall family of reactions denoted by reaction 3,

Reaction 3 $${v}_{{{\mathrm{CO}}}2}{{\rm{CO}}}_{2}+{\rm{Z}}{\cdot }{v}_{{\mathrm{H2O}}}{{\rm{H}}}_{2}{\rm{O}}{\rightleftarrows}{\rm{Z}}{\rm{\cdot }}{v}_{{{\mathrm{CO}}}2}{{\rm{CO}}}_{2}+{v}_{{\mathrm{H2O}}}{{\rm{H}}}_{2}{\rm{O}},$$

where *v*_*i*_ represents the respective stoichiometric coefficients. In this implementation, the CO_2_-rich feed stream and H_2_O-rich feed stream must be fed to opposite sides of the membrane such that CO_2_ and H_2_O can perform the function of releasing one another from the carrier, Z. We also note that, as reaction 3 involves only one carrier, charge neutrality is always achieved, regardless of the charge on Z.

Reaction 3 results in the intimately coupled counter-permeation of H_2_O and CO_2_. Reaction 3 must operate in isolation; otherwise, there will be an ineffective coupling of H_2_O and CO_2_ permeation. For example, the H_2_O driving force required to pump CO_2_ across the membrane could be reduced as a result of leaks, or through alternative permeation mechanisms for H_2_O and CO_2_ that may compete with reaction 3. If a mechanism of the form of reaction 3 occurs in isolation, however, the permeation of CO_2_ can occur against its chemical potential difference. The Gibbs free energy of the system, *G*, must fall on permeation. Now,

1 $$\Delta G={n}_{{{\rm{CO}}_2}}\left({\mu }_{{{\rm{CO}}_{2,\rm{P}}}}-{\mu }_{{{\rm{CO}}_{2,\rm{F}}}}\right)+{n}_{{{\rm{H}}_2{\rm{O}}}}\left({\mu }_{{{\rm{H}}_2{\rm{O}}_{,{\rm{P}}}}}-{\mu }_{{{\rm{H}}_2{\rm{O}}_{,{\rm{F}}}}}\right),$$

where *n*_i_ is the total amount of each species permeated, *μ*_i_ is chemical potential and P and F refer to the permeate and feed sides for CO_2_ and H_2_O, respectively (note that here the CO_2_ feed side is the H_2_O permeate side and vice versa). For the Gibbs free energy of the system to decrease, the decrease in H_2_O chemical potential on permeation must be greater than the increase in CO_2_ chemical potential. Thus, the H_2_O chemical potential at its feed side is higher than at its permeate side (as in conventional downhill membrane operation). However, in the case of CO_2_, its feed side chemical potential is lower than its permeate side (CO_2_ is driven uphill). Rearranging and using the stoichiometric ratios from reaction 3 instead of the permeation ratio,

2 $$\frac{{{\mathrm{d}}G}}{{\mathrm{d}}{n}_{{{\rm{CO}}_2}}}=\left({\mu }_{{{\rm{CO}}_{2,{\rm{P}}}}}-{\mu }_{{{\rm{CO}}_{2,{\rm{F}}}}}\right)-\frac{{v}_{{{\rm{H}}_2{\rm{O}}}}}{{v}_{{{\rm{CO}}_2}}}\left({\mu }_{{{\rm{H}}_2{\rm{O}}_{,{\rm{F}}}}}-{\mu }_{{{\rm{H}}_2{\rm{O}}_{,{\rm{P}}}}}\right),$$

and we note the equilibrium condition

3 $$\left({\mu }_{{{\rm{CO}}_{2,{\rm{P}}}}}-{\mu }_{{{\rm{CO}}_{2,{\rm{F}}}}}\right)=\frac{{v}_{{{\rm{H}}_2{\rm{O}}}}}{{v}_{{{\rm{CO}}_2}}}\left({\mu }_{{{\rm{H}}_2{\rm{O}}_{,{\rm{F}}}}}-{\mu }_{{{\rm{H}}_2{\rm{O}}_{,{\rm{P}}}}}\right),$$

where the H_2_O permeation from its feed to permeate is seen to raise the CO_2_ chemical potential on its permeate side. Assuming the chemical potentials of CO_2_ and H_2_O have an ideal dependence upon partial pressures the equilibrium condition can be rewritten as

4 $$\left(\frac{{P}_{{{\rm{CO}}_{2,{\rm{P}}}}}}{{P}_{{{\rm{CO}}_{2,{\rm{F}}}}}}\right)={\left(\frac{{P}_{{{\rm{H}}_2{\rm{O}}_{,{\rm{F}}}}}}{{P}_{{{\rm{H}}_2{\rm{O}},_{{\rm{P}}}}}}\right)}^{\frac{{v}_{{{\rm{H}}_2{\rm{O}}}}}{{v}_{{{\rm{CO}}_2}}}}.$$

Thus, we observe that the H_2_O partial pressure ratio across the membrane can be used to raise the CO_2_ partial pressure across the membrane.

Previous work on molten-carbonate membranes has used functional supports (electron, ion and mixed conducting)^11–13^. While there have been suggestions, although no strong evidence, that CO_2_ and H_2_O transport might be coupled through reaction 3 (with Z = O^2−^)^14–19^, it is very important to note that such functional supports would lead to alternative permeation mechanisms that would obscure the role of reaction 3 and possibly preclude operation of the membrane for H_2_O-driven uphill CO_2_ permeation. Furthermore, it has been common practice to operate membranes with measurable leaks, which often arise due to difficulties associated with high-temperature sealing. An inert carrier gas is used to measure these leak rates, which are subtracted from the permeation rate to give a ‘leak-free’ permeation rate. Such leaks would further disguise the effect of any reaction 3 and make uphill operation considerably more difficult as a result of back permeation of CO_2_ from its permeate to feed side. Here, to overcome both of these issues, a hot-seal-free (that is, leak-free) alumina support was designed to accommodate a molten carbonate salt (as these salts contain high-mobility carriers with near-ideal CO_2_ selectivity^10,20^). The use of alumina is intended to eliminate permeation mechanisms that rely upon charge transport in the support. Changing the nature of the support can have wider implications as species originating in the support may be dissolved in the molten salt. We also note that, although reaction 3 in some form may occur with an alumina support, it may no longer correspond to reaction 3 with Z = O^2−^.

Truncated conical pores with a diameter of ~100 µm were laser drilled into a closed-end alumina tube and filled with molten carbonate to yield a membrane that could be readily characterized (Fig. 1b, Supplementary Figs. 1 and 2 and Supplementary Table 1). The membrane was operated at >550 °C (to ensure the carbonate salt was molten) using a sweep-gas arrangement, that is, an input stream was introduced to one side of the membrane, and on the other side, a sweep gas was used to generate an output stream (Methods and Supplementary Note 1). Hereafter, these names (input, sweep and output) will be used as feed side and permeate side are now ambiguous terms due to the simultaneous counter-permeation of CO_2_ and H_2_O. Flux (of CO_2_ and H_2_O) was calculated once the measured gas concentration did not change by more than 1% within 0.2 h (sampling rate 10 s) (Supplementary Note 2).

### Downhill CO_2_ permeation

**Fig. 2:**
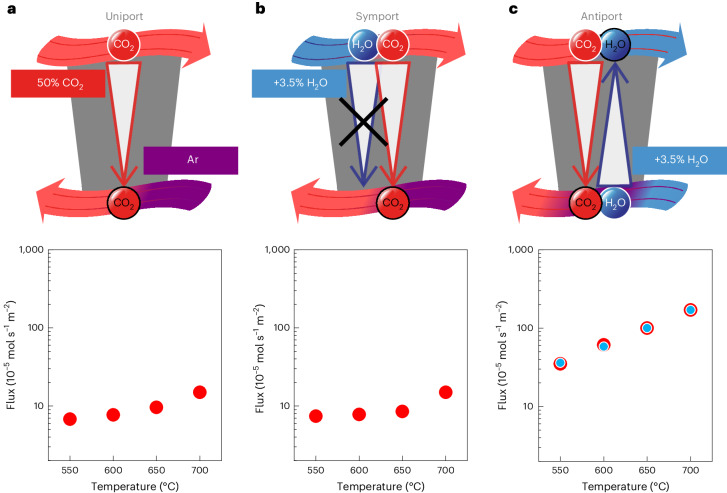
Downhill CO_2_ permeation under dry and humidified conditions. **a**–**c**, Facilitated transport (uniport (**a**), symport (**b**) and antiport (**c**)) in the synthetic membrane. In **a**, a 50% CO_2_/N_2_ input stream and an Ar sweep gas was used. In **b**, humidifying the input stream had no effect on CO_2_ flux and H_2_O did not permeate the membrane. In **c**, humidifying the sweep gas enhanced CO_2_ permeation, and H_2_O permeated the membrane with the same flux as CO_2_ but in the opposite direction, suggesting that the counter-permeation of CO_2_ and H_2_O is linked. The red points are CO_2_, and the blue points are H_2_O.

Initially, a 50% CO_2_ in N_2_ input stream was introduced to the membrane, with Ar used as a sweep gas (Fig. 2a and Supplementary Note 3). In this arrangement, there is a clear downhill concentration gradient and, thus, no energy input is required for CO_2_ permeation (note that both streams are nominally dry with <100 ppm H_2_O). This led to CO_2_ fluxes on the order of 10^−4^ mol s^−1^ m^−2^, much higher than conventional gas separation membranes (for example, polymeric membranes) but in line with expectations for molten-carbonate membranes^20^. Introducing H_2_O at 3.5% to the input stream did not affect the CO_2_ flux, and no H_2_O permeation was observed (Fig. 2b and Supplementary Note 3). However, when H_2_O at 3.5% was introduced to the sweep gas, CO_2_ fluxes increased by an order of magnitude to 10^−3^ mol s^−1^ m^−2^ and H_2_O counter-permeation (in the opposite direction to the permeation of the CO_2_) was apparent with a 1:1 H_2_O:CO_2_ permeation ratio (Fig. 2c and Supplementary Note 3). This suggested that H_2_O and CO_2_ counter-permeation could be linked with perfect 1:1 selectivity (across all conditions in Fig. 2c, an average of 1.02 ± 0.08 CO_2_ molecules are transferred for each H_2_O).

### Uphill CO_2_ permeation

**Fig. 3:**
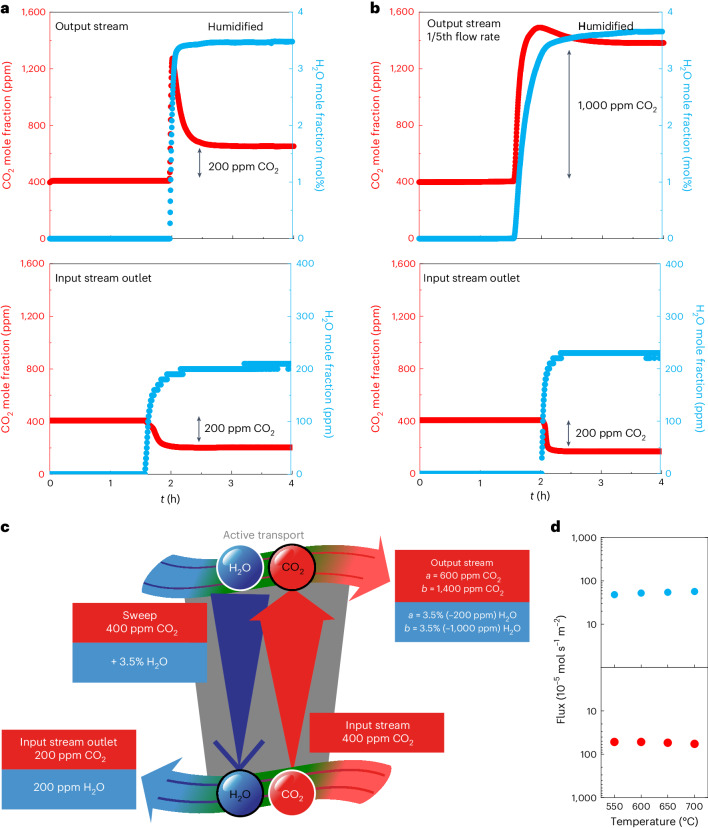
Uphill CO_2_ permeation from dry air into humidified air. **a**, Mole fraction of CO_2_ and H_2_O in the input stream outlet and output stream. Initially, both the input stream and sweep gas (equal flow rates) are dry air; however, at ~2 h the sweep gas is humidified, resulting in uphill permeation of CO_2_ from the input stream to generate a ~600 ppm CO_2_ output stream. **b**, As for **a**, however, the sweep gas flow rate is reduced such that the input stream to sweep gas flow rate ratio is 5:1, resulting in a ~1,400 ppm CO_2_ output stream. Both **a** and **b** were conducted at 550 °C. **c**, Schematic of active transport in the synthetic membrane with two air streams (~400 ppm CO_2_); however, the upper air stream is humidified. H_2_O permeates across the membrane with the same flux as CO_2_ but in the opposite direction. The H_2_O driving force provides the energetic input to transport CO_2_ against its own concentration difference, resulting in a 600 ppm (as for **a**) or 1,400 ppm (as for **b**) CO_2_ output stream. **d**, Plot of flux as a function of temperature (equal flow rates). The red points are CO_2_, and the blue points are H_2_O. CO_2_ flux is plotted on an inverted axis to highlight that CO_2_ flux is uphill.

To exploit the link between H_2_O and CO_2_ permeation, air was fed to both sides of the membrane at equal flow rates. One of these air streams must now be denoted as the input stream, while the other is the sweep. As both streams contain ~400 ppm (~0.04%) CO_2_, there is no CO_2_ concentration difference across the membrane, and the input stream concentration is reduced by three orders of magnitude compared with the experiments in Fig. 2. Indeed, no CO_2_ permeation is observed for the first ~2 h (Fig. 3a and Supplementary Note 4). However, at ~2 h, H_2_O at 3.5% was introduced to the sweep gas (Fig. 3a, top), and ~200 ppm of H_2_O permeates to the input stream (Fig. 3a, bottom). Therefore, as there is a 1:1 H_2_O:CO_2_ permeation ratio, ~200 ppm of CO_2_ is removed from the input stream (Fig. 3a, bottom), which permeates across the membrane in the opposite direction to produce a 600 ppm output stream (Fig. 3a, top). Note that there is an overshoot in the CO_2_ evolved from the membrane into the output stream. This is due to a change in the level of hydration of the membrane in the presence of H_2_O; the hydration process results in the ejection of CO_2_ (reaction 3 and Supplementary Note 5). Overall, the results in Fig. 3a show that the humidity difference between the two air streams supplies the energy required to pump CO_2_ across the membrane, resulting in removal of CO_2_ from the input stream and concentration of CO_2_ in the output stream.

The membrane in Fig. 3a captures 50% of the CO_2_ in air, as ~200 ppm of CO_2_ is removed from the input stream. The ~600 ppm output stream, and ~200 ppm remaining in the input stream, results in an enrichment ratio of 3:1 across the membrane. However, by using non-equal air flow rates (a flow rate ratio of 5:1 here), we can raise the CO_2_ concentration in the output stream further to ~1,400 ppm while maintaining 50% CO_2_ capture from air, resulting in an enrichment ratio of 7:1 (Fig. 3b and Supplementary Note 6). The same ~200 ppm of CO_2_ is removed from the input stream, but this now results in a ~1,000 ppm increase of CO_2_ in the output stream raising the CO_2_ concentration from ~400 ppm to ~1,400 ppm. Thus, the membrane can increase the concentration of CO_2_ on permeation; modifying the flow rate ratio of the two air streams allows one to pump CO_2_ further uphill while maintaining the same CO_2_ capture efficiency. Comparing Fig. 3a and Fig. 3b, we also note here the change in relative time constants associated with the H_2_O and CO_2_ concentration responses in both the input and output streams (this is expected due to the different flow rates used).

Remarkably, the membrane maintained extremely high CO_2_ fluxes (10^−3^ mol s^−1^ m^−2^) (Fig. 3c), even though the CO_2_ input stream concentration was reduced by three orders of magnitude in comparison with the cases shown in Fig. 2.

Thus, in principle, both the thermodynamic and kinetic challenges of dilute separations have been addressed in this system. First, the concentration of CO_2_ is increased on permeation. Second, H_2_O appears to address a rate-limiting step of molten carbonate membranes (Supplementary Note 7)^21^, the release of CO_2_ into the output stream. This contributes to an increase in the flux of CO_2_ from air to levels normally observed with input streams of much higher CO_2_ concentration, as the substantial H_2_O driving force now dictates the CO_2_ flux.

### Membrane performance benchmarking and permeation mechanism

It is very difficult to fairly compare the performance of this membrane with that of other CO_2_-permeable membranes. First, permeability is widely used in the membrane community for comparison purposes as it relates only to the intrinsic properties of the membrane. However, it is not usually clear if bulk properties are rate controlling, as most studies do not identify rate-determining steps (we note that, in our work, it appears that a surface-exchange step may be rate-limiting; Supplementary Note 7). Second, a conventional definition of CO_2_ permeability is of little help here as the direction of CO_2_ permeation is now opposing the direction of the CO_2_ concentration difference. Finally, the operating conditions of membranes reported in the literature are not usually sufficiently described to allow detailed comparison. For example, it is clear here that H_2_O has a very important effect on flux, yet relevant H_2_O concentrations are rarely determined (Supplementary Note 2).

Nonetheless, although a direct comparison should only be attempted with caution, we can supply some context by looking at previous work. To achieve the magnitude (but not the direction) of CO_2_ fluxes reported in Fig. 3, state-of-the-art gas separation membranes would require a driving force of between 4,000 and 4 × 10^6^ Pa CO_2_ (Supplementary Note 2)^10,22^. We must recall here that the membrane in Fig. 3 was supplied with a 40 Pa CO_2_ input stream (40 Pa being approximately equal to 400 ppm at atmospheric pressure). Thus, it is clear that this membrane not only serves to concentrate CO_2_ (uphill permeation), but it does so while delivering unparalleled kinetic performance.

Considering the potential for application of the membrane to, for example, direct air capture, we note that raising the concentration of CO_2_ from air by any substantial factor will decrease the flow rate to any further downstream separation and, thus, decrease the size and capital cost of this further separation. Therefore, such a pre-concentration stage is likely to be very important. Furthermore, the membrane is durable and practical, as it was operated for ~50 days continuously (Supplementary Note 8 and Supplementary Table 2) and can achieve 20% CO_2_ capture from air using H_2_O at levels readily achievable in any real process, that is, those that occur naturally over a diurnal cycle (Supplementary Note 9).

Regarding permeation mechanism, we note that, among many theoretically stable species in molten carbonate salts^23,24^, previous work has provided support for oxide-^14,25–27^ and carbonate-like species^28–30^ as likely CO_2_ carriers. Thus, we suggest that such species act as a common carrier (Z in reaction 3) for both H_2_O and CO_2_ here. For high fluxes, similar concentrations of Z·H_2_O and Z·CO_2_ in the melt are required; if Z·H_2_O forms too readily, the CO_2_ flux will be restricted because of the lack of Z·CO_2_. For example, if the H_2_O:CO_2_ ratio on both sides of the membrane were equal to unity then the ratio of carrier species in the melt, Z·H_2_O:Z·CO_2_, would be unity at equilibrium if the Δ*G* of reaction 3 were 0 kJ mol^−1^. However, due to concentration effects (the two orders of magnitude higher concentration of H_2_O), Δ*G* should be increased in order that the carrier binds less strongly to H_2_O.

**Fig. 4:**
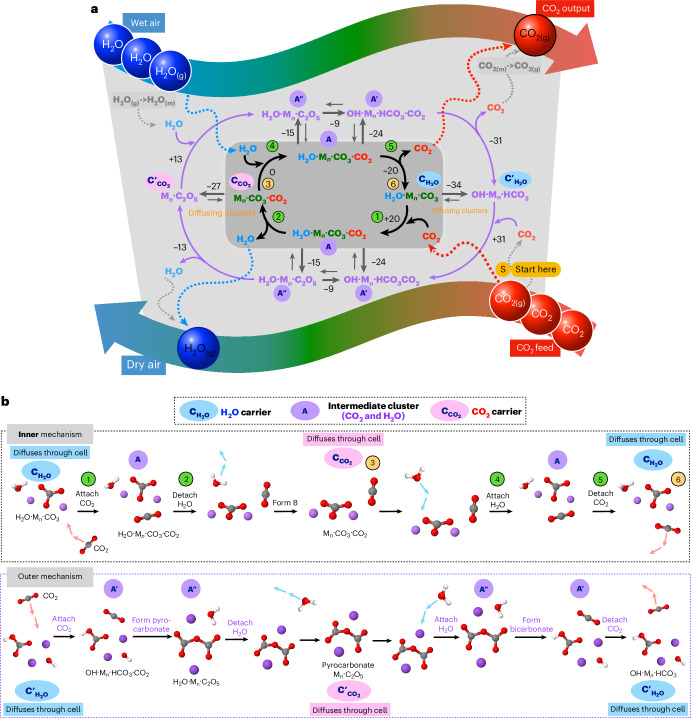
CO_2_ and H_2_O transport in molten carbonate. **a**, Chemical representation of the computed mechanisms. **b**, Representative structures showing the inner (adduct based) and outer (covalent bond breaking/making) mechanistic pathways. Atom colouring: C, grey; O, red; H, white; alkali metal, purple. Chemical transformation steps are indicated by numbered green circles and physical diffusion by orange circles. Inner mechanism: (1) CO_2_ is absorbed into the melt and interacts with a water adduct alkali-carbonate cluster C_H2O_ [H_2_O·M_*n*_·CO_3_] to form an adduct cluster A [H_2_O·M_*n*_·CO_3_·CO_2_]. (2) Subsequently, water dissociates from A, releasing a CO_2_ carrier species C_CO2_ [M_*n*_·CO_3_·CO_2_], which is a CO_2_ adduct alkali-carbonate cluster. (3) C_CO2_ ‘diffuses’ through the melt. (4) Near the surface, [M_*n*_·CO_3_·CO_2_] reacts with water to again form A [H_2_O·M_*n*_·CO_3_·CO_2_]. (5) A subsequently releases CO_2_ and reforms C_H2O_ [H_2_O·M_*n*_·CO_3_]. (6) C_H2O_ then diffuses due to the concentration gradient in H_2_O, starting the transport cycle again. The C_CO2_, A and C_H2O_ adducts can also undergo chemical reactions and, hence, exist in equilibria with other species; A′ [M_*n*_·C_2_O_5_·H_2_O], A′ [HCO_3_·M_*n*_·OH·CO_2_], C′_CO2_ pyrocarbonate [M_*n*_·C_2_O_5_] and C′_H2O_ bicarbonate/hydroxide [HCO_3_·M_*n*_·OH]. The outer mechanism between species C′_CO2_, A′, A′ and C′_H2O_ follow a similar cycle but now with transient pyrocarbonate and bicarbonate species diffusing through the melt.

Molecular density functional theory (DFT) calculations were carried out to explore the thermodynamics of potential reactions of H_2_O and CO_2_ with likely carriers in the melt (details are provided in Supplementary Discussion). Multiple viable mechanistic pathways incorporating complex equilibria and a range of active species and carrier forms have been successfully identified (Fig. 4a,b). The formation/dissociation of the Z·CO_2_ carrier cluster is facilitated by one molecule of H_2_O, and the formation/dissociation of the Z·H_2_O carrier cluster is facilitated by one molecule of CO_2_. Intermediates (that contain H_2_O and CO_2_ in a 1:1 ratio) A = H_2_O·M_*n*_·CO_3_·CO_2_, where M_*n*_ represents the local interacting alkali cations (*n* = 1, 2, 3), are accessible (inner mechanism), as are pyrocarbonate A′ = H_2_O·M_*n*_·C_2_O_5_ and bicarbonate A′ = OH·M_*n*_·HCO_3_·CO_2_ species (outer mechanism). We note that Z = O^2−^ did not appear to be a viable route for permeation. However, when, for example, oxygen-ion-conducting supports are used, dissolution of species originating from the support could provide alternative mechanisms not seen here.

**Fig. 5:**
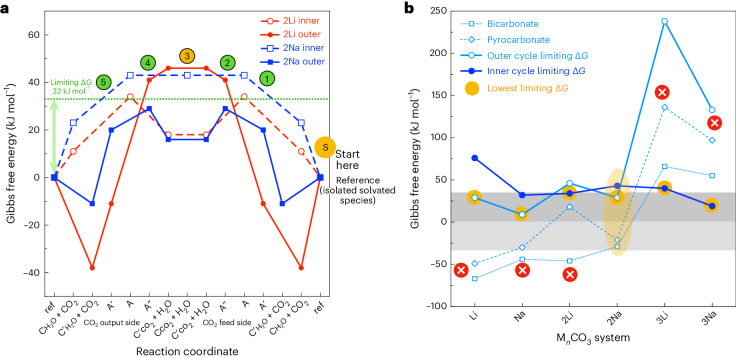
Computed Gibbs free energies for CO_2_ and H_2_O transport within the molten carbonate. **a**, Graph of the Gibbs free energies (kJ mol^−1^) for each step evaluated for solvated M_2_CO_3_ M = Li, Na, systems in the melt at 550 °C, demonstrating the competitive nature of both mechanisms. The lines are guides to the eye. On the reaction coordinate, ref refers to the isolated solvated species. **b**, Comparison of the limiting Δ*G* for all systems examined; the accessible region (0 to ±32 kJ mol^−1^) is shaded light grey. The outer cycle carrier species, bicarbonate C′_H2O_ and pyrocarbonate C′_CO2_ can be stabilized, retarding the mechanism. For M_*n*_CO_3_, only one pathway is viable (yellow highlight) due to either a reaction retarding resting state or high-energy intermediate. The lines are guides to the eye. All species’ energy cycles are available in Supplementary Discussion.

Gibbs free energies (DFT) of the relevant reactions were computed at 550 °C including a generalized solvent environment and evaluated under appropriate H_2_O and CO_2_ concentration ratios. Gibbs free energy profiles for representative mechanisms are depicted in Fig. 5a for 2Li and 2Na. To be viable, pathways must have accessible low-energy intermediates and no substantially stabilized species that ‘halt’ the permeation process (Fig. 5b). To be accessible, the Δ*G* between the reference state and highest-energy structures along a pathway must be less than the energy input from the H_2_O concentration difference across the membrane (~32 kJ mol^−1^). Overall, examining the limiting Gibbs free energies for *n* = 1, 2 and 3, accessible inner mechanisms are determined for 1Na, 2Li, 3Li and 3Na species, and an accessible outer mechanism is determined for the 2Na species.

## Conclusions

We have shown that it is possible, in principle, to address the thermodynamic and kinetic challenges of dilute separations through the careful design of a membrane system. To increase the chemical potential of a dilute species on permeation, the decrease in chemical potential of a second species was exploited. Furthermore, transferring the large chemical potential driving force associated with the second species to a kinetically limited step yielded exceedingly high permeabilities of the dilute species. This was made possible by careful membrane design that eliminated competing processes, that is, leaks and alternative permeation mechanisms, and ‘locked together’ the two species in a 1:1 permeation ratio. We demonstrated the concept using naturally occurring humidity differences in air, exploiting the H_2_O driving force to pump CO_2_ from one air stream to another while raising the concentration of CO_2_ (with a CO_2_ enrichment ratio of up to 7:1 in a single stage). One could envisage the membrane being used for direct air capture in a cascade of similar membranes, or as a pre-concentration stage before other CO_2_ separation processes that operate at higher CO_2_ concentrations. Beyond CO_2_ capture, we expect that our work will provide inspiration for the design of innovative membrane processes across a range of dilute separation applications.

## Methods

### Background and motivation of the closed-end tube membrane design

A previously unresolved engineering issue is sealing ceramic membranes at high temperature. The choice of sealant is strongly dependent on operating conditions and application; routine sealants include ceramics, noble metals and glasses. Such sealants can reduce the effective surface area for permeation and lead to performance degradation. Furthermore, leaks from failed sealants can reduce driving forces, which can lead to lower performance. From a fundamental point of view, leaks limit the quality of mechanistic/kinetic data one can extract from membrane experiments. In the case of supported molten-salt membranes, seals may also interact with the molten salt, which can corrode the seal and potentially change melt composition, that is, both membrane durability and performance. Having no hot seal is therefore a major design change that addresses multiple problems at once. Here we designed a closed-end tube membrane support (one end closed, one end open) that only required a low-temperature sealant on the open end of the tube (Supplementary Fig. 2). Leaks were below the detectable limit of our analytical instrumentation.

### Membrane support fabrication and characterization

The closed-end Al_2_O_3_ tube was custom-made with outer diameter (OD), internal diameter (ID) and length (L) 19.1 mm OD × 12.7 mm ID × 235 mm L. The closed end of the tube was hand polished to ~0.5 mm thickness using a Perspex holder and polishing paper (Struers, Silicon Carbide Grinding Paper, 220 grit) on a rotary polisher (Struers, Knuth Rotor). The polishing holder was a Perspex tube (60 mm OD × 19.1 mm ID × 180 mm L), specifically designed to fit the Al_2_O_3_ tube, with two plastic screws (M6) at 30 mm distance from each end of the holder to keep the closed-end tube perpendicular to the polishing surface. Pores were laser drilled perpendicular to the polished closed end at Laser Micromachining Limited. Supplementary Fig. 1 shows greyscale *XY* orthoslices from an X-ray micro-computed tomography reconstructions of the drilled tube. Due to the inherent Gaussian shape of the laser beam, the artificial pores exhibited a truncated conical shape (Supplementary Fig. 1c). Due to variation in the closed-end tube thickness, not all pores were through pores. A summary of the artificial pore properties calculated from the micro-computed tomography reconstructions is given in Supplementary Table 1.

### Preparation of the eutectic carbonate mixture

A carbonate eutectic mixture (32 wt% Li_2_CO_3_, 33 wt% Na_2_CO_3_ and 35 wt% K_2_CO_3_) was chosen as the ion-conducting molten salt phase because of its low melting point (~400 °C) and high ionic conductivity. The individual powders were dried at 300 °C for 24 h, ground with a mortar and pestle to reduce the particle size and then homogenized in a mixing container (Fluxana, MU-K-Mixer_50Hz) with three plastic balls of 9 mm diameter for ≥0.5 h. According to the characteristics listed in Supplementary Table 1, the volume of the artificial pores was ~0.017 cm^3^, and thus ~0.04 g of carbonates was required to fully infiltrate the artificial pores (the density of the eutectic carbonate mixture is ~2.3 g cm^−3^). The required amount of eutectic mixture was placed in a 12-mm-diameter stainless-steel die and pressed at 5 tons for 1 min (Atlas Series Hydraulic Presses T28, Specac).

### Supported molten-salt membrane preparation

The supported molten-salt membrane was prepared by placing the eutectic carbonate pellet on the laser drilled end of the tube, followed by heating beyond the melting point of the carbonates (~400 °C). This resulted in carbonate infiltration into the artificial pores of the alumina tube. Heating was carried out at a rate of 1 °C min^−1^ under a 30 cm^3^ (standard temperature and pressure, STP) min^−1^ flow of 50% CO_2_/N_2_ on both sides of the membrane to avoid carbonate decomposition. All experiments were performed with the same membrane support. Carbonates were periodically removed, using deionized water and sonication, and the membrane support was re-infiltrated with fresh carbonates, as detailed in Supplementary Table 2.

### Closed-end tube supported molten-salt membrane reactor

The membrane reactor, which was in a temperature-programmable split-tube furnace (Vecstar, VST/1150), is shown in Supplementary Fig. 2. To perform permeation experiments, the membrane support was sealed at the open end with a high-vacuum silicone grease (Dow Corning High Vacuum Grease) into a stainless-steel holder that was screwed into the base of the stainless-steel reactor. Two alumina tubes of 3 mm diameter were used to introduce the input stream and sweep gas, and a third with a closed-end was used as a thermocouple guide (RS Components, Pro K Type Thermocouple, RS 787-7793). This system (3 mm alumina tubes and closed-end tube supported molten-salt membrane) was enclosed in a quartz tube that was sealed with an O-ring to the stainless-steel base of the reactor. Thus, the reactor comprised two chambers: an internal chamber and an external chamber, which were enclosed by the supported molten-salt membrane support and the quartz tube, respectively. The volumes of the two chambers were estimated to be ~30 and ~170 cm^3^, respectively. The residence time distribution for the two chambers was determined experimentally by switching between flowing N_2_ and Ar (30 cm^3^ (STP) min^−1^). Before each switch, sufficient time was provided for the inlet and outlet compositions to reach a constant mole fraction, and the change in mole fraction following a switch was characterized (Supplementary Fig. 3). If there are no dead or stagnant zones within the reactor, then the chamber volumes determined experimentally should be close to the estimated volumes. The experimental volume of the internal and external chambers is 22.5 and 150 cm^3^, respectively. Furthermore, as the residence time distribution response of the chambers can be described by a model for well-mixed volumes, the membrane can be considered to be exposed to the outlet conditions of each chamber.

### Membrane reactor flow system

The flow system used for membrane experiments is shown in Supplementary Fig. 4. The H_2_O content in humidified gas streams was controlled using two water baths (Grant, R2, GD100). Using ice in the water bath (maintaining the temperature at ~0 °C), 0.6% H_2_O in the gas stream was achieved, and 3.5% H_2_O was achieved by adjusting the temperature of the water bath to 30 °C. A hygrometer (Vaisala, F2520137) was connected to the water bath outlet to monitor the H_2_O mole fraction in the gas stream. The output streams from the membrane reactor were analysed with two CO_2_/H_2_O infrared (IR) analysers (LI-COR, LI-840A). Additionally, a mass spectrometer (HIDEN, HALO 100-RC) was connected in series with the CO_2_/H_2_O IR analyser to record N_2_ (used to indicate any trans-membrane leaks, as described in ‘Membrane testing methodology’ section). The mass spectrometer was calibrated using pure Ar or 400 ppm N_2_/400 ppm O_2_/Ar. The calibration was performed every 12 h to account for drift of the detector. The IR analysers were calibrated before experiments using a 3-point calibration with Ar, 380 ppm CO_2_ in Ar and 1% CO_2_ in Ar. The calibration of H_2_O was conducted by the manufacturer using a 3-point calibration with Ar, 1 and 35 ppt H_2_O. To prevent condensation of H_2_O in the humidified gas streams, silicone rubber heating tapes (Watlow, series EHG) were used on certain sections of tubing. The heating tapes were connected to a temperature controller with a thermocouple sensor to maintain the temperature at ~60 °C.

### Membrane testing methodology

For permeation experiments, a flow rate of 30 cm^3^ (STP) min^−1^ was used in most cases for both the input and sweep gas streams (exceptions to this are explained in detail where they arise). Both internal and external chambers were operated at atmospheric pressure (tested with an inline pressure gauge fitted between the mass flow controllers and the two reactor chamber inlets). All gases were provided by BOC (certification level B, ±2% uncertainty, certification by analysis), and compositions are given on a molar basis. Heating and cooling of the membrane was always performed under symmetrical gas conditions (that is, input stream and sweep gas of 50% CO_2_/N_2_)_._ Membrane temperature was varied from 550 to 700 °C in 50 °C increments with heating rates of 1 °C min^−1^. Gas flow rates were regulated using mass flow controllers (Brooks SLA5850). In the downhill experiments with an Ar sweep gas, N_2_ (as a component of the input stream) was used to indicate any trans-membrane leaks and to estimate CO_2_ leak rates, if present, owing to its similar kinetic diameter to CO_2_. However, the N_2_ mole fraction in the output stream was always below the detectable limit of our analytical instrumentation (~1 ppm). In the uphill experiments where air was used as both the input stream and sweep gas (and, therefore, N_2_ was present in the output stream), the closure of the CO_2_ material balance across the two streams was confirmed.

### Computational details

Calculations were carried out using Gaussian G09 and G16 and visualized using Gaussview 6 (refs. ^31–33^). The DFT B3LYP functional has been employed, with a 6-311G(d,p) basis set, an ultrafine integration grid consisting of (pruned) 99 radial shells and 590 angular points per shell. Convergence criteria are 10^−9^ on the root-mean-square density matrix and 10^−7^ on the energy. All structures have been optimized under no symmetry constraints, and critical points are confirmed via a frequency analysis (no imaginary modes). Frequency analysis also delivers thermochemical data G, H and TS. Thermochemical data have been evaluated at 298.15, 823.15 (550 °C) and 973.15 (700 °C) using the freqchk utility employing the default options of standard state pressure (1 atm) and scaling of harmonic frequencies by 0.8928. Further details of the solvent environment and details of the evaluation of reaction energies can be found in Supplementary Discussion.

## Supplementary information

Supplementary Information Supplementary Figs. 1–4, Tables 1 and 2, Notes 1–9, Discussion and References 1–13.

## Data Availability

The experimental datasets generated during and/or analysed during the current study are available in the Newcastle University research repository at 10.25405/data.ncl.21550713 (ref. ^34^). The computational data are available on Zenodo at 10.5281/zenodo.11063599 (ref. ^35^).

## References

[1] Sholl, D. S. & Lively, R. P. Seven chemical separations to change the world. *Nature* **532**, 435–437 (2016). 27121824 10.1038/532435a

[2] Erans, M. et al. Direct air capture: process technology, techno-economic and socio-political challenges. *Energy Environ. Sci.* **15**, 1360–1405 (2022).

[3] McQueen, N. et al. A review of direct air capture (DAC): scaling up commercial technologies and innovating for the future. *Prog. Energy* **3**, 032001 (2021).

[4] Bui, M. et al. Carbon capture and storage (CCS): the way forward. *Energy Environ. Sci.* **11**, 1062–1176 (2018).

[5] Realmonte, G. et al. An inter-model assessment of the role of direct air capture in deep mitigation pathways. *Nat. Commun.* **10**, 1–12 (2019). 31332176 10.1038/s41467-019-10842-5 PMC6646360

[6] McQueen, N. et al. Cost analysis of direct air capture and sequestration coupled to low-carbon thermal energy in the United States. *Environ. Sci. Technol.* **54**, 7542–7551 (2020). 32412237 10.1021/acs.est.0c00476

[7] Keith, D., Heidel, K. & Cherry, R. in *Geo-engineering Climate Change. Environmental Necessity or Pandora’s Box?* (eds Launder, B. & Thompson, J.) 107–126 (Cambridge Univ. Press, 2010).

[8] Park, H. B., Kamcev, J., Robeson, L. M., Elimelech, M. & Freeman, B. D. Maximizing the right stuff: the trade-off between membrane permeability and selectivity. *Science* **356**, 1138–1148 (2017). 10.1126/science.aab0530 28619885

[9] Alberts, B. et al. *Molecular Biology of the Cell* (Garland Science, 2002).

[10] Mutch, G. A. et al. Supported molten-salt membranes for carbon dioxide permeation. *J. Mater. Chem. A* **7**, 12951–12973 (2019).

[11] Rui, Z., Anderson, M., Lin, Y. S. & Li, Y. Modeling and analysis of carbon dioxide permeation through ceramic-carbonate dual-phase membranes. *J. Memb. Sci.* **345**, 110–118 (2009).

[12] Wade, J. L., Lackner, K. S. & West, A. C. Transport model for a high temperature, mixed conducting CO_2_ separation membrane. *Solid State Ion* **178**, 1530–1540 (2007).

[13] Zhang, L. et al. Fast electrochemical CO_2_ transport through a dense metal-carbonate membrane: a new mechanistic insight. *J. Memb. Sci.* **468**, 373–379 (2014).

[14] Xing, W. et al. Steam-promoted CO_2_ flux in dual-phase CO_2_ separation membranes. *J. Memb. Sci.* **482**, 115–119 (2015).

[15] Sun, S., Wen, Y. & Huang, K. A new ceramic-carbonate dual-phase membrane for high-flux CO_2_ capture. *ACS Sustain. Chem. Eng.* **9**, 5454–5460 (2021).

[16] Sun, S., Billings, A., Zhang, K. & Huang, K. Direct, efficient and selective capture of low concentration of CO_2_ from natural gas flue gas using a high temperature tubular carbon capture membrane. *J. Memb. Sci.* **661**, 120929 (2022).

[17] Chen, T. et al. Coupling CO_2_ separation with catalytic reverse water-gas shift reaction via ceramic-carbonate dual-phase membrane reactor. *Chem. Eng. J.* **379**, 122182 (2020).

[18] Pang, B. et al. Mixed oxygen ionic‐carbonate ionic conductor membrane reactor for coupling CO_2_ capture with in situ methanation. *AIChE J.* **69**, e17919 (2022).

[19] Zhang, K., Sun, S., Xu, N. & Huang, K. H_2_O-enhanced CO_2_ transport through a proton conducting ceramic-molten carbonate dual-phase membrane. *J. Memb. Sci.* **650**, 120421 (2022).

[20] McNeil, L. A. et al. Dendritic silver self-assembly in molten-carbonate membranes for efficient carbon dioxide capture. *Energy Environ. Sci.* **13**, 1766–1775 (2020).

[21] Kazakli, M. et al. Controlling molten carbonate distribution in dual-phase molten salt-ceramic membranes to increase carbon dioxide permeation rates. *J. Memb. Sci.* **617**, 118640 (2021).

[22] Comesana-Gandara, B. et al. Redefining the Robeson upper bounds for CO_2_/CH_4_ and CO_2_/N_2_ separations using a series of ultrapermeable benzotriptycene-based polymers of intrinsic microporosity. *Energy Environ. Sci.* **12**, 2733–2740 (2019).

[23] Carper, W. R., Wahlbeck, P. G. & Griffiths, T. R. DFT models of molecular species in carbonate molten salts. *J. Phys. Chem. B* **116**, 5559–5567 (2012). 22512323 10.1021/jp3016694

[24] Cassir, M., Moutiers, G. & Devynck, J. Stability and characterization of oxygen species in alkali molten carbonate: a thermodynamic and electrochemical approach. *J. Electrochem. Soc.* **140**, 0–9 (1993).

[25] Xing, W. et al. Improved CO_2_ flux by dissolution of oxide ions into the molten carbonate phase of dual-phase CO_2_ separation membranes. *Sep. Purif. Technol.* **212**, 723–727 (2019).

[26] Evans, A., Xing, W. & Norby, T. Electromotive force (EMF) determination of transport numbers for native and foreign ions in molten alkali metal carbonates. *J. Electrochem. Soc.* **162**, F1135–F1143 (2015).

[27] Cerón, M. R. et al. Surpassing the conventional limitations of CO_2_ separation membranes with hydroxide/ceramic dual-phase membranes. *J. Memb. Sci.* **567**, 191–198 (2018).

[28] Zhang, L. et al. First spectroscopic identification of pyrocarbonate for high CO_2_ flux membranes containing highly interconnected three dimensional ionic channels. *Phys. Chem. Chem. Phys.* **15**, 13147–13152 (2013). 23824146 10.1039/c3cp52362d

[29] Corradini, D., Coudert, F. & Vuilleumier, R. Carbon dioxide transport in molten calcium carbonate occurs through an oxo-Grotthuss mechanism via a pyrocarbonate anion. *Nat. Chem.* **8**, 454–460 (2016). 27102679 10.1038/nchem.2450

[30] Claes, P., Moyaux, D. & Peeters, D. Solubility and solvation of carbon dioxide in the molten Li_2_CO_3_/Na_2_CO_3_/K_2_CO_3_ (43.5:31.5:25.0 mol-%) eutectic mixture at 973 K I. Experimental part. *Eur. J. Inorg. Chem.* **3**, 583–588 (1999).

[31] Frisch, M. J. et al. *Gaussian 09, Revision D.01* (Gaussian, 2013).

[32] Frisch, M. J. et al. *Gaussian 16, Revision C.01* (Gaussian, 2016).

[33] Dennington, R., Keith, T. A. & Millam, J. M. *GaussView, Version 6.1* (Semichem, 2016).

[34] Metcalfe, I. S. et al. Permeation data for "Separation and concentration of CO_2_ from air using a humidity-driven membrane" 10.25405/data.ncl.21550713 (Newcastle University, 2024). 10.1038/s41560-024-01588-6 PMC11420091 39324020

[35] Hunt, P. carbonate_reactions_QC_data_files. *Zenodo*10.5281/zenodo.11063599 (2024).

