# Supplementary material for: Separation and concentration of CO2 from air using a humidity-driven molten-carbonate membrane
Source: Nat Energy. 2024 Jul 19;9(9):1074–83. doi: 10.1038/s41560-024-01588-6 (PMC11420091; doi:10.1038/s41560-024-01588-6)
Supplement: Supplementary file 1 — Supplementary Figs. 1–4, Tables 1 and 2, Notes 1–9, Discussion and References 1–13. [file 41560_2024_1588_MOESM1_ESM.pdf]

nature energy

Supplementary information https://doi.org/10.1038/s41560-024-01588-6

Separation and concentration of CO_2 from air using a humidity-driven molten-carbonate membrane

In the format provided by the authors and unedited

Table of Contents

**Supplementary Figures**	**1**
Supplementary Figure 1	1
Supplementary Figure 2	2
Supplementary Figure 3	3
Supplementary Figure 4	4
**Supplementary Tables**	**5**
Supplementary Table 1	5
Supplementary Table 2	6
**Supplementary Notes**	**14**
Supplementary Note 1	14
Supplementary Note 2	15
Supplementary Note 3	18
Supplementary Note 4	19
Supplementary Note 5	21
Supplementary Note 6	23
Supplementary Note 7	25
Supplementary Note 8	26
Supplementary Note 9	28
**Supplementary Discussion**	**30**
**Supplementary References**	**53**

Supplementary Figures

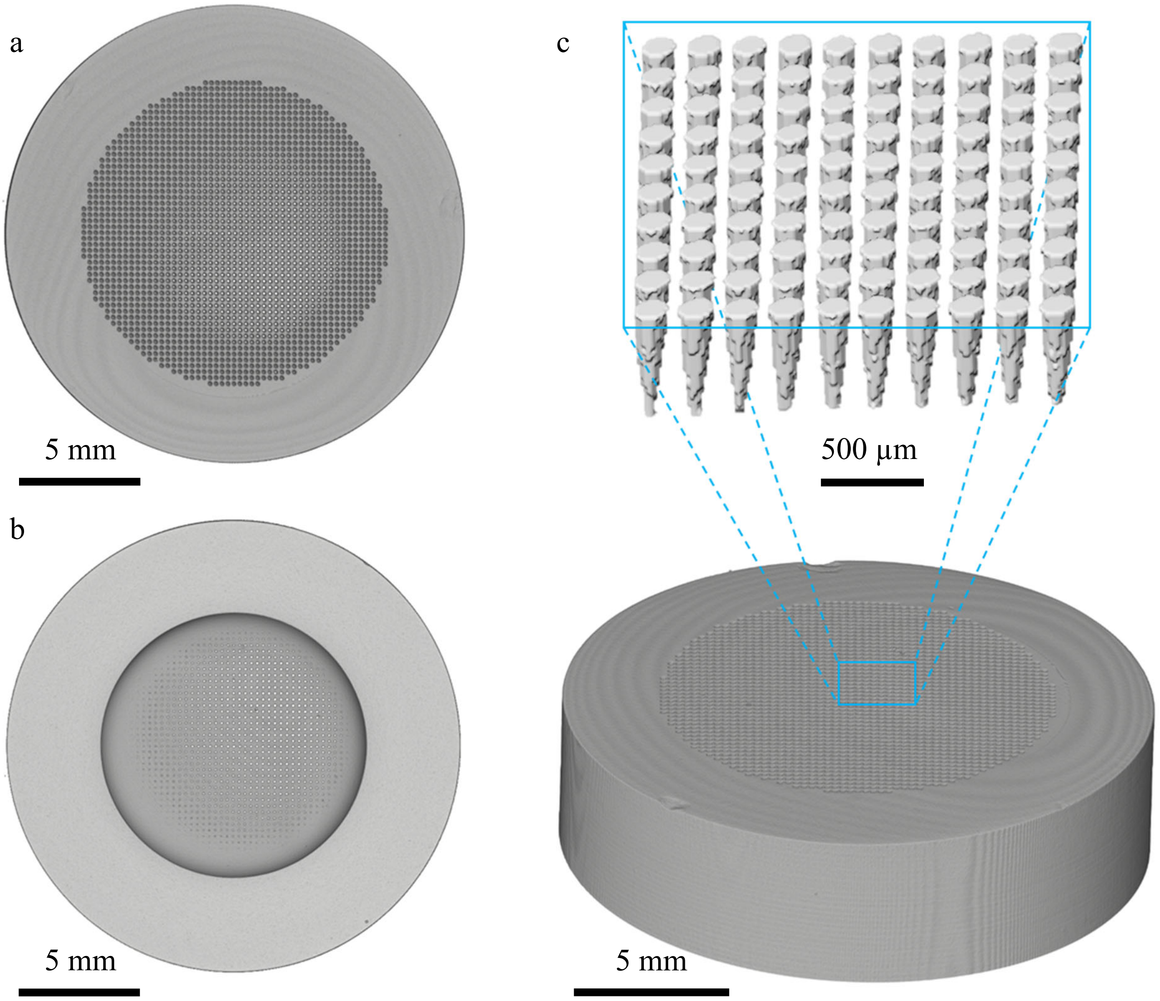

Supplementary Figure 1. X-ray micro-computed tomography reconstructions. a, Top and **b**, bottom surface of the laser-drilled closed-end tube membrane. Top and bottom surfaces refer to the laser incident and opposite surface respectively. **c**, 3D reconstruction of a selected region of the laser drilled pore space showing the truncated conical shape of the pores.

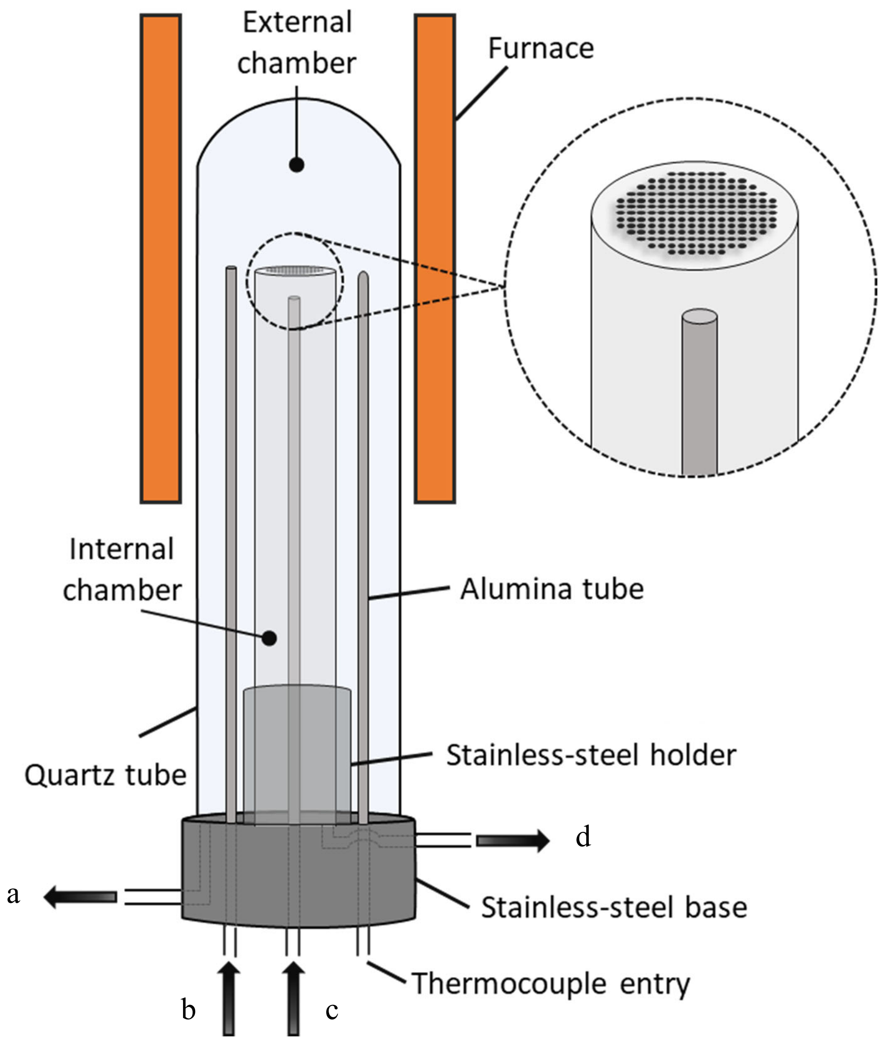

Supplementary Figure 2. Closed-end tube supported molten-salt membrane reactor. a, External chamber (gas) outlet, **b**, external chamber (gas) inlet, **c**, internal chamber (gas) inlet and **d**, internal chamber (gas) outlet. The internal and external chambers are enclosed by the closed-end tube supported molten-salt membrane and a quartz tube respectively. Reprinted (adapted) with permission from *ACS Appl. Mater. Interfaces* **12**, 16436-16641 (2020). Copyright 2020 American Chemical Society.[4]

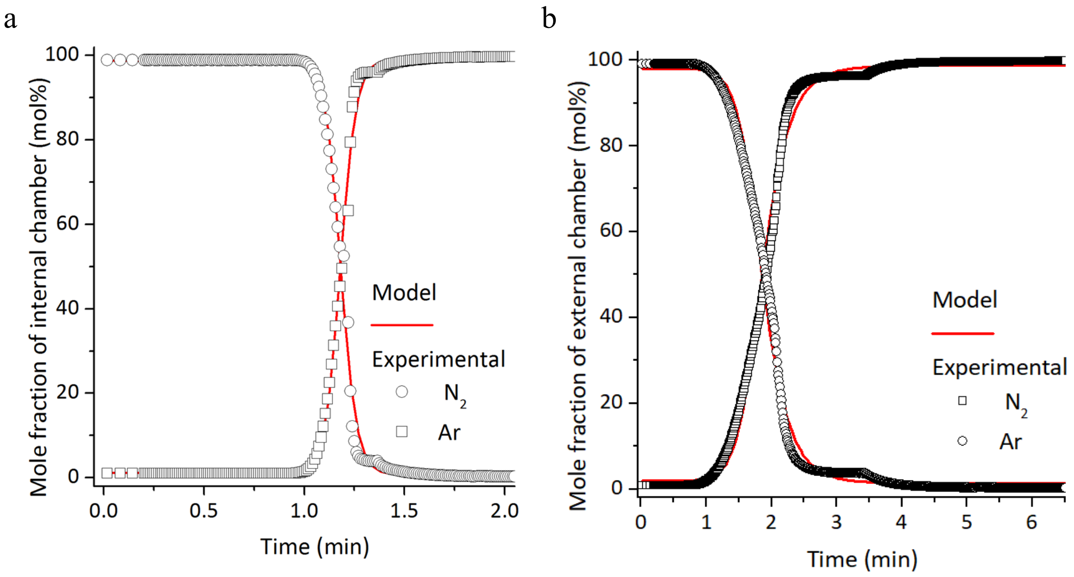

Supplementary Figure 3. Residence time distribution (RTD) of the membrane reactor. a, Internal and **b**, external chamber RTD. Data was collected with switches between flowing N_2 and Ar (30 cm^3 (STP) min^{-1}). Mean residence times for internal and external chambers are ~45 and ~300 s respectively. Red lines indicate fitting of a logistic function to experimental data.

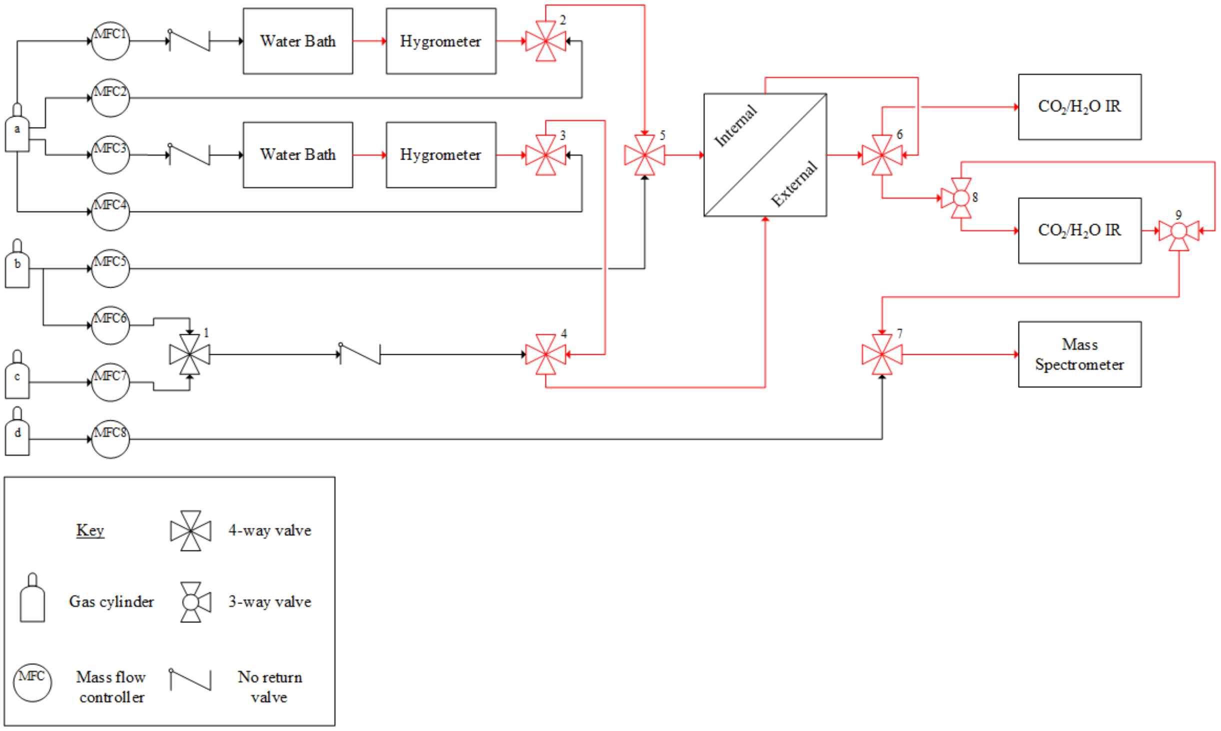

Supplementary Figure 4. Membrane reactor flow system. Gas cylinders a - c were changed based on the experiment (see detail below). Gas cylinder d supplies a calibration mixture to the mass spectrometer. Black lines are stainless steel or PTFE 1/8th inch tubing, with red colouring indicating that these were heated lines. Valve 1 was used to switch between gases to be delivered to the external chamber during heating up and permeation. Valves 2 and 3 were used to switch between dry (nominally dry with <100 ppm H_2O) and humidified gases delivered to the internal and external chamber respectively. Valves 4 and 5 were used to switch between different gas compositions delivered to the internal and external chamber respectively. Valve 6 was used to switch between the analyzers for the analysis of the internal chamber and external chamber gas outlets. Valve 7 was used to switch between analysis of the gas outlets and calibration of the mass spectrometer (conducted every 12 h). Note that any unconnected 4-way valve ports imply that this port was connected to a vent line for extraction. Valves 8 and 9 were used to bypass the CO_2/H_2O IR analyser when the outlet streams contained high CO_2 concentration (above the 2% saturation limit of the detector). No return valves were used to avoid damage to the MFCs from any reverse flow of the humidified gas streams. For some experiments a t-piece was added after selected MFCs such that inert and CO_2-containing gases could be blended to achieve various CO_2 mole fractions.

Supplementary Tables

Supplementary Table 1. Laser drilled artificial pore properties. Summary of laser-drilled pore properties calculated from micro-CT analysis.

Total number of pores	2030
Number of through pores	925 ± 15
Average diameter of pores on top surface, mm	0.177 ± 0.017
Average diameter of pores on bottom surface, mm	0.072 ± 0.002
Centre-to-centre pitch of pores, mm	0.246 ± 0.005
Average base thickness, mm	0.77 ± 0.05
Total volume of all pores, mm^3	17.2 ± 0.7
Total volume of through pores, mm^3	7.8 ± 0.3
Surface area of pores on top surface, mm^2	34.8 ± 2.8
Surface area of pores on bottom surface, mm^2	3.0 ± 1.3

Supplementary Table 2. Operating conditions for the supported molten-salt membrane. Temperature, duration and gas compositions at inlet and outlet of internal and external chambers in chronological order during approximately 50 days of membrane operation. As several experimental conditions are returned to numerous times, this table facilitates statistical analysis of the entire dataset associated with the work. For example, for experiments with a 50% CO_2 feed gas and an Ar sweep gas at 700 °C, the average CO_2 concentration in the outlet is 71 ppm (standard deviation 3 ppm, n = 16) which translates to an error smaller than the data points used in the article figures. Cooling implies that carbonates were re-infiltrated as detailed in the materials section.

T °C	Duration, h	Internal chamber gas inlet	External chamber gas inlet	Internal chamber %CO_2 outlet	External chamber %CO_2 outlet
Heating at 1 °C min^{-1}					
550	14.0	50% CO_2 /N_2	Ar	50	-
550	3.8	90% CO_2 /N_2	Ar	90	0.0043
550	3.6	50% CO_2 /N_2	Ar	50	0.0036
550	3.5	77% CO_2 /N_2	Ar	77	0.0038
550	3.0	90% CO_2 /N_2	Ar	90	0.0040
550	3.0	25% CO_2 /N_2	Ar	25	0.0020
550	3.2	50% CO_2 /N_2	3.5%H_2O/Ar	50	0.0162
550	4.4	50% CO_2 /N_2	Ar	50	0.0025
550	4.0	50% CO_2 /N_2	3.5%H_2O/Ar	50	0.0150
550	6.1	50% CO_2 /N_2	Ar	50	0.0029
550	8.1	Ar	50% CO_2 /N_2	0.0037	50
550	5.2	Ar	3.5%H_2O/50% CO_2 /N_2	0.0039	50
Heating at 1 °C min^{-1}					
600	10.6	50% CO_2 /N_2	Ar	50	0.0033
600	4.7	77% CO_2 /N_2	Ar	77	0.0042
600	3.5	90% CO_2 /N_2	Ar	90	0.0044
600	1.6	25% CO_2 /N_2	Ar	25	0.0032
600	8.3	50% CO_2 /N_2	Ar	50	0.0037
600	4.2	50% CO_2 /N_2	3.5%H_2O/Ar	50	0.0272
600	3.0	50% CO_2 /N_2	Ar	50	0.0035
600	4.3	Ar	50% CO_2 /N_2	0.0028	50
600	0.9	Ar	3.5%H_2O/50% CO_2 /N_2	0.0031	50
Heating at 1 °C min^{-1}					
650	12.6	50% CO_2 /N_2	Ar	50	0.0053
650	1.5	77% CO_2 /N_2	Ar	77	0.0056
650	0.5	90% CO_2 /N_2	Ar	90	0.0058
650	1.1	25% CO_2 /N_2	Ar	25	0.0050
650	0.3	50% CO_2 /N_2	Ar	50	0.0053
650	8.6	50% CO_2 /N_2	3.5%H_2O/Ar	50	0.0422
650	0.6	50% CO_2 /N_2	Ar	50	0.0041

650	9.6	Ar	50% CO_2 /N_2	0.0038	50
650	1.2	Ar	3.5%H_2O/50% CO_2 /N_2	0.0044	50
650	0.5	50% CO_2 /N_2	Ar	50	0.0043
Heating at 1 °C min^{-1}					
700	8.5	50% CO_2 /N_2	Ar	50	0.0071
700	1.2	77% CO_2 /N_2	Ar	77	0.0071
700	1.3	90% CO_2 /N_2	Ar	90	0.0072
700	1.0	25% CO_2 /N_2	Ar	25	0.0069
700	0.2	50% CO_2 /N_2	Ar	50	0.0071
700	3.2	50% CO_2 /N_2	3.5%H_2O/Ar	50	0.0703
700	1.0	50% CO_2 /N_2	Ar	50	0.0068
700	4.8	50% CO_2 /N_2	3.5%H_2O/Ar	50	0.0675
700	2.9	50% CO_2 /N_2	Ar	50	0.0075
700	7.0	Ar	50% CO_2 /N_2	0.0051	50
700	4.3	Ar	3.5%H_2O/50% CO_2 /N_2	0.0043	50
700	8.1	50% CO_2 /N_2	Ar	50	0.0073
700	2.2	77% CO_2 /N_2	Ar	77	0.0079
700	0.7	50% CO_2 /N_2	Ar	50	0.0071
700	1.2	90% CO_2 /N_2	Ar	90	0.0084
700	0.8	50% CO_2 /N_2	Ar	50	0.0069
Cooling at 1 °C min^{-1}					
Heating at 1 °C min^{-1}					
550	11.2	50% CO_2 /N_2	Ar	50	0.0031
550	6.5	50% CO_2 /N_2	1000 ppmCO_2/Ar	50	0.1025
550	1.7	90% CO_2 /N_2	1000 ppmCO_2/Ar	90	0.1043
550	0.7	77% CO_2 /N_2	1000 ppmCO_2/Ar	77	0.1039
550	1.2	25% CO_2 /N_2	1000 ppmCO_2/Ar	25	0.1021
550	12.4	50% CO_2 /N_2	380 ppmCO_2/Ar	50	0.0399
550	1.7	77% CO_2 /N_2	380 ppmCO_2/Ar	77	0.0403
550	1.7	90% CO_2 /N_2	380 ppmCO_2/Ar	90	0.0405
550	2.7	25% CO_2 /N_2	380 ppmCO_2/Ar	25	0.0392
550	3.3	50% CO_2 /N_2	Ar	50	0.0029
550	2.6	50% CO_2 /N_2	3.5%H_2O/Ar	50	0.0153
550	7.8	1000 ppmCO_2/Ar	1000 ppmCO_2/Ar	0.1	0.1024
550	1.6	50% CO_2 /N_2	1000 ppmCO_2/Ar	50	0.1006
550	6.5	50% CO_2 /N_2	3.5%H_2O/ 1000 ppmCO_2/Ar	50	0.1100
550	2.2	1000 ppmCO_2/Ar	1000 ppmCO_2/Ar	0.1	0.1000
550	1.1	50% CO_2 /N_2	1000 ppmCO_2/Ar	50	0.1016
550	11.7	50% CO_2 /N_2	Ar	50	0.0022
550	8.9	380 ppmCO_2/Ar	380 ppmCO_2/Ar	0.0380	0.0380
550	1.8	50% CO_2 /N_2	380 ppmCO_2/Ar	50	0.0403
550	0.6	380 ppmCO_2/Ar	380 ppmCO_2/Ar	0.0380	0.0380

550	7.1	50% CO_2 /N_2	3.5%H_2O/ 380 ppmCO_2/Ar	50	0.1120
550	0.9	50% CO_2 /N_2	Ar	50	0.0022
		Cooling at 1 °C min^{-1}			
		Heating at 1 °C min^{-1}			
550	14.4	50% CO_2 /N_2	Ar	50	0.0025
550	2.6	77% CO_2 /N_2	Ar	77	0.0032
550	3.2	90% CO_2 /N_2	Ar	90	0.0034
550	1.4	50% CO_2 /N_2	Ar	50	0.0024
550	2.2	3.5%H_2O/50% CO_2 /N_2	Ar	50	0.0023
550	4.2	25% CO_2 /N_2	Ar	25	0.0015
550	3.4	50% CO_2 /N_2	Ar	50	0.0024
550	10.1	50% CO_2 /N_2	3.5%H_2O/Ar	50	0.0185
550	3.3	50% CO_2 /N_2	Ar	50	0.0025
550	11.3	50% CO_2 /N_2	1.1% CO_2 /N_2	50	1.1013
550	7.2	1.1% CO_2 /N_2	1.1% CO_2 /N_2	1.1	1.1000
550	3.9	90% CO_2 /N_2	1.1% CO_2 /N_2	90	1.1025
550	4.2	77% CO_2 /N_2	1.1% CO_2 /N_2	77	1.1020
550	5.9	25% CO_2 /N_2	1.1% CO_2 /N_2	25	1.1009
550	4.2	50% CO_2 /N_2	1.1% CO_2 /N_2	50	1.1013
550	3.9	50% CO_2 /N_2	380 ppmCO_2/Ar	50	0.0400
550	3.9	380 ppmCO_2/Ar	380 ppmCO_2/Ar	0.0380	0.0380
550	3.4	50% CO_2 /N_2	380 ppmCO_2/Ar	50	0.0401
550	3.9	90% CO_2 /N_2	380 ppmCO_2/Ar	90	0.0414
550	4.3	77% CO_2 /N_2	380 ppmCO_2/Ar	77	0.0410
550	5.7	50% CO_2 /N_2	380 ppmCO_2/Ar	50	0.0401
550	3.3	25% CO_2 /N_2	380 ppmCO_2/Ar	25	0.0395
550	5.1	50% CO_2 /N_2	Ar	50	0.0028
550	8.5	50% CO_2 /N_2	380 ppmCO_2/Ar	50	0.0400
550	11.3	50% CO_2 /N_2	1.1% CO_2 /N_2	50	1.1012
550	4.4	1.1% CO_2 /N_2	1.1% CO_2 /N_2	1.1	1.1000
550	15.5	50% CO_2 /N_2	Ar	50	0.0028
		Heating at 1 °C min^{-1}			
600	10.9	50% CO_2 /N_2	Ar	50	0.0034
600	5.0	3.5%H_2O/50% CO_2 /N_2	Ar	50	0.0034
600	4.4	50% CO_2 /N_2	Ar	50	0.0034
600	4.4	50% CO_2 /N_2	3.5%H_2O/Ar	50	0.0269
		Heating at 1 °C min^{-1}			
650	10.2	50% CO_2 /N_2	Ar	50	0.0041
650	5.4	3.5%H_2O/50% CO_2 /N_2	Ar	50	0.0040
650	4.8	50% CO_2 /N_2	Ar	50	0.0041
650	4.8	50% CO_2 /N_2	3.5%H_2O/Ar	50	0.0471

Heating at 1 °C min^{-1}					
700	10.1	50% CO_2 /N_2	Ar	50	0.0067
700	5.4	3.5%H_2O/50% CO_2 /N_2	Ar	50	0.0065
700	4.1	50% CO_2 /N_2	Ar	50	0.0067
700	5.3	50% CO_2 /N_2	3.5%H_2O/Ar	50	0.0739
Cooling at 1 °C min^{-1}					
Heating at 1 °C min^{-1}					
450	13.5	50% CO_2 /N_2	Ar	50	0.0015
450	1.5	3.5%H_2O/50% CO_2 /N_2	Ar	50	0.0015
450	0.5	50% CO_2 /N_2	Ar	50	0.0016
450	6.1	50% CO_2 /N_2	3.5%H_2O/Ar	50	0.0030
450	1.0	50% CO_2 /N_2	Ar	50	0.0015
Heating at 1 °C min^{-1}					
500	10.9	50% CO_2 /N_2	Ar	50	0.0017
500	2.3	3.5%H_2O/50% CO_2 /N_2	Ar	50	0.0017
500	8.5	50% CO_2 /N_2	Ar	50	0.0018
500	2.4	50% CO_2 /N_2	3.5%H_2O/Ar	50	0.0070
500	0.9	50% CO_2 /N_2	Ar	50	0.0016
Heating at 1 °C min^{-1}					
550	12.8	50% CO_2 /N_2	Ar	50	0.0026
550	12.0	380 ppmCO_2/Ar	380 ppmCO_2/Ar	0.0380	0.0380
550	9.8	380 ppmCO_2/Ar	3.5%H_2O 439 ppmCO_2/Ar	0.0325	0.0481
550	5.3	380 ppmCO_2/Ar	380 ppmCO_2/Ar	0.0380	0.0380
550	5.9	380 ppmCO_2/Ar	3.5%H_2O 439 ppmCO_2/Ar	0.0324	0.0480
550	4.4	380 ppmCO_2/Ar	380 ppmCO_2/Ar	0.0380	0.0380
550	0.6	50% CO_2 /N_2	Ar	50	0.0026
Heating at 1 °C min^{-1}					
700	15.8	50% CO_2 /N_2	Ar	50	0.0071
700	3.0	50% CO_2 /N_2	3.5%H_2O/Ar	50	0.0730
700	4.2	50% CO_2 /N_2	Ar	50	0.0067
700	8.0	1.1% CO_2 /N_2	1.1% CO_2 /N_2	1.1	1.1000
700	5.2	50% CO_2 /N_2	1.1% CO_2 /N_2	50	1.1025
700	1.0	90% CO_2 /N_2	1.1% CO_2 /N_2	90	1.1031
700	1.2	77% CO_2 /N_2	1.1% CO_2 /N_2	77	1.1033
700	0.9	25% CO_2 /N_2	1.1% CO_2 /N_2	25	1.1021
700	1.1	50% CO_2 /N_2	1.1% CO_2 /N_2	50	1.1025
700	9.2	439 ppmCO_2/Ar	439 ppmCO_2/Ar	0.0439	0.0439
700	5.7	439 ppmCO_2/Ar	Ar	0.0435	0.0004
700	2.1	1.1% CO_2 /N_2	Ar	1.1	0.0009
700	1.9	50% CO_2 /N_2	Ar	50	0.0071

700	3.3	50% CO_2 /N_2	439 ppmCO_2/Ar	50	0.0487
700	1.1	90% CO_2 /N_2	439 ppmCO_2/Ar	90	0.0496
700	1.5	50% CO_2 /N_2	439 ppmCO_2/Ar	50	0.0488
700	0.9	90% CO_2 /N_2	439 ppmCO_2/Ar	90	0.0495
700	1.3	77% CO_2 /N_2	439 ppmCO_2/Ar	77	0.0493
700	0.8	25% CO_2 /N_2	439 ppmCO_2/Ar	25	0.0483
700	0.9	50% CO_2 /N_2	439 ppmCO_2/Ar	50	0.0498
700	8.3	439 ppmCO_2/Ar	439 ppmCO_2/Ar	0.0439	0.0439
700	8.5	50% CO_2 /N_2	Ar	50	0.0071
700	3.0	50% CO_2 /N_2	439 ppmCO_2/Ar	50	0.0487
700	2.6	50% CO_2 /N_2	1.1% CO_2 /N_2	50	1.1025
700	6.1	1.1% CO_2 /N_2	1.1% CO_2 /N_2	1.1	1.1000
700	3.3	50% CO_2 /N_2	1.1% CO_2 /N_2	50	1.1025
			Cooling at 1 °C min^{-1}		
			Heating at 1 °C min^{-1}		
550	6.7	50% CO_2 /N_2	Ar	50	0.0031
550	1.1	90% CO_2 /N_2	Ar	90	0.0042
550	0.9	77% CO_2 /N_2	Ar	77	0.0036
550	1.3	25% CO_2 /N_2	Ar	25	0.0027
550	1.1	50% CO_2 /N_2	Ar	50	0.0031
550	1.2	50% CO_2 /N_2	439 ppmCO_2/Ar	50	0.0457
550	4.2	439 ppmCO_2/Ar	439 ppmCO_2/Ar	0.0439	0.0439
550	1.8	90% CO_2 /N_2	439 ppmCO_2/Ar	90	0.0462
550	1.1	77% CO_2 /N_2	439 ppmCO_2/Ar	77	0.0460
550	1.3	25% CO_2 /N_2	439 ppmCO_2/Ar	25	0.0449
550	0.8	50% CO_2 /N_2	1.1% CO_2 /N_2	50	1.1011
550	6.5	1.1% CO_2 /N_2	1.1% CO_2 /N_2	1.1	1.1000
550	2.1	50% CO_2 /N_2	1.1% CO_2 /N_2	50	1.1011
550	6.7	1.1% CO_2 /N_2	1.1% CO_2 /N_2	1.1	1.1000
550	2.3	50% CO_2 /N_2	1.1% CO_2 /N_2	50	1.1011
550	5.2	1.1% CO_2 /N_2	1.1% CO_2 /N_2	1.1	1.1000
550	2.7	90% CO_2 /N_2	1.1% CO_2 /N_2	90	1.1023
550	6.3	1.1% CO_2 /N_2	1.1% CO_2 /N_2	1.1	1.1000
550	2.9	77% CO_2 /N_2	1.1% CO_2 /N_2	77	1.1018
550	5.8	1.1% CO_2 /N_2	1.1% CO_2 /N_2	1.1	1.1000
550	2.9	25% CO_2 /N_2	1.1% CO_2 /N_2	25	1.1008
550	1.1	50% CO_2 /N_2	Ar	50	0.0031
550	1.1	90% CO_2 /N_2	Ar	90	0.0042
550	0.9	77% CO_2 /N_2	Ar	77	0.0036
550	0.7	25% CO_2 /N_2	Ar	25	0.0027
550	1.1	50% CO_2 /N_2	1.1% CO_2 /N_2	50	1.1010
550	5.0	1.1% CO_2 /N_2	1.1% CO_2 /N_2	1.1	1.1000
550	0.9	90% CO_2 /N_2	1.1% CO_2 /N_2	90	1.1023
550	6.7	1.1% CO_2 /N_2	1.1% CO_2 /N_2	1.1	1.1000
550	1.1	77% CO_2 /N_2	1.1% CO_2 /N_2	77	1.1017

550	1.3	25% CO_2 /N_2	1.1% CO_2 /N_2	25	1.1007
550	1.1	50% CO_2 /N_2	439 ppmCO_2/Ar	50	0.0457
550	1.4	90% CO_2 /N_2	439 ppmCO_2/Ar	90	0.0462
550	1.4	77% CO_2 /N_2	439 ppmCO_2/Ar	77	0.0460
550	0.9	25% CO_2 /N_2	439 ppmCO_2/Ar	25	0.0449
550	4.9	439 ppmCO_2/Ar	439 ppmCO_2/Ar	0.0439	0.0439
Heating at 1 °C min^{-1}					
700	9.4	50% CO_2 /N_2	439 ppmCO_2/Ar	50	0.0492
700	1.1	90% CO_2 /N_2	439 ppmCO_2/Ar	90	0.0450
700	0.9	77% CO_2 /N_2	439 ppmCO_2/Ar	77	0.0498
700	0.8	25% CO_2 /N_2	439 ppmCO_2/Ar	25	0.0488
700	9.3	50% CO_2 /N_2	Ar	50	0.0075
700	1.3	90% CO_2 /N_2	Ar	90	0.0087
700	0.9	77% CO_2 /N_2	Ar	77	0.0083
700	1.3	25% CO_2 /N_2	Ar	25	0.0068
700	2.2	1.1% CO_2 /N_2	1.1% CO_2 /N_2	1.1	1.1
700	3.8	50% CO_2 /N_2	1.1% CO_2 /N_2	50	1.1030
700	1.1	90% CO_2 /N_2	1.1% CO_2 /N_2	90	1.1036
700	1.3	77% CO_2 /N_2	1.1% CO_2 /N_2	77	1.1038
700	0.9	25% CO_2 /N_2	1.1% CO_2 /N_2	25	1.1026
700	2.1	1.1% CO_2 /N_2	1.1% CO_2 /N_2	1.1	1.1
Cooling at 1 °C min^{-1}					
Heating at 1 °C min^{-1}					
550	12.8	50% CO_2 /N_2	Ar	50	0.0028
550	5.8	air	air	0.0409	0.0409
550	3.2	air	3.5%H_2O/air	0.0171	0.0639
550	3.3	air	air	0.0409	0.0409
550	3.1	air	3.5%H_2O/air	0.0175	0.0637
550	3.5	air	air	0.0409	0.0409
550	3.3	air	3.5%H_2O/air	0.0171	0.0631
550	3.7	air	air	0.0409	0.0409
Heating at 1 °C min^{-1}					
600	9.1	air	air	0.0409	0.0409
600	4.0	air	3.5%H_2O/air	0.0170	0.0588
600	5.2	air	air	0.0409	0.0409
600	3.5	air	3.5%H_2O/air	0.0171	0.0579
600	3.2	air	air	0.0409	0.0409
Heating at 1 °C min^{-1}					
650	5.8	air	air	0.0409	0.0409
650	3.5	air	3.5%H_2O/air	0.0155	0.0580
650	3.7	air	air	0.0409	0.0409
650	3.9	air	3.5%H_2O/air	0.0159	0.0577
650	4.2	air	air	0.0409	0.0409
Cooling at 1 °C min^{-1}					

Heating at 1 °C min^{-1}					
550	7.8	50% CO_2 /N_2	Ar	50	-
550	9.1	air	air	0.0409	0.0409
550	3.1	air	3.5%H_2O/air	0.0175	0.0637
550	2.8	air	air	0.0409	0.0409
550	3.2	air	3.5%H_2O/air	0.0170	0.0650
550	3.2	air	air (flow↓)	0.0409	0.0409
550	3.1	air	3.5%H_2O/air (flow↓)	0.0190	0.1380
550	2.8	air	air	0.0409	0.0409
550	3.1	air	3.5%H_2O/air	0.0160	0.0640
550	3.3	air	air	0.0409	0.0409
550	3.3	air	air	0.0409	0.0409
Cooling at 1 °C min^{-1}					
Heating at 1 °C min^{-1}					
550	9	50% CO_2 /N_2	Ar	50	-
550	2.5	air	air	0.0409	0.0409
550	1.3	air	air (flow↓)	0.0409	0.0409
550	5.1	air	3.5%H_2O/air (flow↓)	0.0200	0.1383
550	3.1	air	air (flow↓)	0.0409	0.0409
550	2.5	air	air	0.0409	0.0409
550	2.5	air	3.5%H_2O/air	0.169	0.0651
550	2.5	air	air	0.0409	0.0409
550	3.3	0.6%H_2O/air	3.5%H_2O/air	0.0330	0.0485
550	3.0	air	air	0.0409	0.0409
550	5.8	50% CO_2 /N_2	Ar	50	0.0024
550	7.5	Ar	Ar	-	-
550	6.7	50% CO_2 /N_2	Ar	50	0.0023
Heating at 1 °C min^{-1}					
600	6.1	air	air	0.0409	0.0409
600	3.9	air	3.5%H_2O/air	0.0175	0.0583
600	5.5	air	air	0.0409	0.0409
600	4.0	air	3.5%H_2O/air	0.0172	0.0580
600	2.2	air	air	0.0409	0.0409
Heating at 1 °C min^{-1}					
650	7.8	air	air	0.0409	0.0409
650	7.2	air	3.5%H_2O/air	0.0165	0.0575
650	2.2	air	air	0.0409	0.0409
650	3.9	air	3.5%H_2O/air	0.0161	0.0577
650	2.5	air	air	0.0409	0.0409
Heating at 1 °C min^{-1}					
700	7.5	air	air	0.0409	0.0409
700	3.7	air	3.5%H_2O/air	0.0150	0.0562
700	2.1	air	air	0.0409	0.0409

700	3.6	air	3.5%H_2O/air	0.0148	0.0566
700	5.8	air	air	0.0409	0.0409
Cooling at 1 °C min^{-1}					
Heating at 1 °C min^{-1}					
700	12.6	50% CO_2 /N_2	Ar	50	0.0079
700	5.9	air	air	0.0409	0.0409
700	1.6	3.5%H_2O/air	air	0.0780	0.0136
700	2.2	3.5%H_2O/air	3.5%H_2O/air	0.0409	0.0409
700	2.0	air	3.5%H_2O/air	0.0154	0.0670
700	8.1	air	air	0.0409	0.0409
700	3.3	air	3.5%H_2O/air	0.0153	0.0661
700	3.1	3.5%H_2O/air	3.5%H_2O/air	0.0409	0.0409
700	2.7	3.5%H_2O/air	air	0.0552	0.0248
700	4.3	air	air	0.0409	0.0409
Cooling at 1 °C min^{-1}					
Heating at 1 °C min^{-1}					
550	22	air	air	0.0409	0.0409
550	5	0.6%H_2O/air	3.5%H_2O/air	0.0333	0.0476
550	6	air	air	0.0409	0.0409
Cooling at 1 °C min^{-1}					
Heating at 1 °C min^{-1}					
700	12.4	50% CO_2 / N_2	Ar	50	0.0076
700	5.9	air	air	0.0409	0.0409
700	4.2	3.5%H_2O/air	3.5%H_2O/air	0.0409	0.0409
700	4.1	air	air	0.0409	0.0409
700	4.4	3.5%H_2O/air	3.5%H_2O/air	0.0409	0.0409
700	4.1	air	air	0.0409	0.0409
700	4.4	3.5%H_2O/air	3.5%H_2O/air	0.0409	0.0409
700	3.9	air	air	0.0409	0.0409
Cooling at 1 °C min^{-1}					

Supplementary Notes

Supplementary Note 1. Membrane operation. To facilitate discussion of membrane operation, several terms must be defined. The feed and permeate sides of the membrane refer to the 'faces' of the membrane. Thus, an input stream is introduced to the feed side, and on the permeate side, a sweep gas is used to generate an output stream. Unless stated otherwise, the flow rates on either side of the membrane are equal. Internal and external chambers differentiate the volumes enclosed by the supported molten-salt membrane support and quartz tube reactor housing, respectively (Supplementary Figure 2). Inlet and outlet are appended to stream or chamber to *e.g.*, define where gas composition was measured. Symmetrical conditions indicate that both input and sweep streams are of the same composition, whereas asymmetrical indicates distinct gas compositions (and thus a driving force for permeation). Downhill or uphill (permeation) illustrates whether a species permeates towards lower or higher concentration, respectively. We refer to internal and external chambers when discussing the underlying data, but when results are presented in the main text, we assign one side of the membrane to be input and one to be sweep or output. This assignment depends upon the nature of the experiment and in many cases demonstrating uphill permeation is arbitrary. Supplementary Table 2 summarises the entire set of operating conditions, in chronological order, under which the membrane was investigated during approximately 50 days of operation.

Supplementary Note 2. Calculation of flux, and permeability. For the downhill experiments, permeation rates are calculated using the flow rate (Q) and the average mole fractions of H_2O or CO_2 at steady state in the outlet stream of the side they permeated into (usually in the external chamber gas). The mole fractions were taken once steady CO_2 levels do not change more than 1% within 0.2 h (sampling rate 10 s). However, for the uphill experiments, and to measure rate accurately, CO_2 permeation rates need to be calculated in a different manner because of the presence of CO_2 in the inlet to the permeate side (sweep stream). The rates are calculated from the difference in CO_2 mole fraction (at steady state) in the dry, or drier, outlet stream between a symmetrical condition (sym) with no permeation and an asymmetrical condition (asym) with permeation and the flow rate (Q'), where $'$ refers to the dry side of the membrane (usually the internal chamber gas):

$$\mathrm{CO_2\ permeation\ rate(uphill)} = \left(x'_{\mathrm{CO_2(sym)}} - x'_{\mathrm{CO_2(asym)}}\right) \cdot Q' \qquad \text{Equation 1}$$

CO_2 mole fractions during asymmetrical conditions are measured on a wet basis (IR analyser readings of the humidified streams in the outlet of the reactor). CO_2 material balances were performed by measuring the CO_2 content in the stream outlets of both sides of the membrane during uphill experiments. The closure of the CO_2 material balance was confirmed at steady state and the rate of consumption of CO_2 on the internal chamber gas outlet was equal to the rate of CO_2 evolution on the external chamber gas outlet (where $'$ refers to the dry side and $''$ to the humidified side of the membrane):

$$\overbrace{\left(x'_{\mathrm{CO_2(sym)}} - x'_{\mathrm{CO_2(asym)}}\right) \cdot Q'}^{\text{rate of consumption}} = \overbrace{\left(x''_{\mathrm{CO_2(asym)}} - x''_{\mathrm{CO_2(sym)}}\right) \cdot Q''}^{\text{rate of evolution}} \qquad \text{Equation 2}$$

CO_2 flux ($j_{\mathrm{CO_2}}$) is calculated by defining an active membrane area, A:

$$j_{\mathrm{CO_2}} = \frac{\mathrm{CO_2\ permeation\ rate}}{A} \qquad \text{Equation 3}$$

Here, we approximate this area as the total volume of through pores divided by average base thickness (both from Supplementary Table 1), arriving at an area on the order of 10^{-5} m^2.

Flux through a membrane depends upon the permeability (P_M) multiplied by a driving force and divided by the membrane thickness (l). Here we use the average base thickness from Supplementary Table 1 (on the order of 10^{-4} m). Normally, for a CO_2 permeable membrane, it is assumed that the driving force is simply the CO_2 partial pressure difference across the membrane:

$$j_{\mathrm{CO_2}} = \frac{P_{M,\mathrm{CO_2}}}{l} \left(P_{\mathrm{CO_2}}{}' - P_{\mathrm{CO_2}}{}''\right) \qquad \text{Equation 4a}$$

Recent work on supported molten-salt membranes,[1] however, has suggested that when transport in the molten-carbonate phase is the rate-determining step for permeation, the flux depends on the natural log ratio of CO_2 partial pressures. In this case, we denote permeability as $\hat{P}_M$:

$$j_{CO_2} = \frac{\hat{P}_{M,CO_2}}{l} \; ln\frac{P_{CO_2}'}{P_{CO_2}''}$$ Equation 4b

However, here we are no longer dealing with the independent permeation of a species (see Figure 3 for example of coupled, counter permeation of CO_2 and H_2O). The flux across the membrane depends upon the combined driving force arising from both CO_2 and H_2O partial pressure asymmetry. In this case, we denote permeability as $P_{M,\text{counter}}$ and $\hat{P}_{M,\text{counter}}$:

$$j_{CO_2} = \frac{P_{M,\text{counter},CO_2}}{l} \; \{(P_{CO_2}' - P_{CO_2}'') + (P_{H_2O}'' - P_{H_2O}')\}$$ Equation 5a

Or,

$$j_{CO_2} = \frac{\hat{P}_{M,\text{counter},CO_2}}{l} \; ln\frac{P_{CO_2}'}{P_{CO_2}''}\frac{P_{H_2O}''}{P_{H_2O}'}$$ Equation 5b

If we are to compare this work to the literature, we require a benchmark. Permeability may at first appear to be useful as it is, in principle, an intrinsic property of the membrane. Clearly, however, permeability is only of use generally (i.e., for membranes of different thickness) if the rate is controlled by bulk processes and not interfacial processes. As most studies do not identify rate-determining steps, it is therefore not usually clear if bulk properties are rate controlling. For example, as discussed in Supplementary Note 7, we highlight that in our work, a surface-exchange step appears to be rate-limiting.

Nonetheless, we can attempt to provide context by benchmarking against previous work. Most CO_2 separation membranes are polymeric, with state-of-the-art membranes having a CO_2 permeability on the order of 10^{-13} mol m^{-1} s^{-1} Pa^{-1}.[2] Whilst supported molten-salt membranes have not been studied to the same extent, previous literature reports CO_2 permeabilities between 10^{-12} and 10^{-10} mol m^{-1} s^{-1} Pa^{-1} for downhill CO_2 permeation.[3] To achieve the magnitude (but not the direction) of the CO_2 fluxes reported in our work (10^{-3} mol s^{-1} m^{-2}) with a 40 Pa (10^1 Pa) CO_2 input stream (i.e., the maximum possible CO_2 partial pressure using air at atmospheric pressure as an input stream) and a practical minimum membrane thickness (10^{-4} m), permeability would have to be multiple orders of magnitude higher than previously reported. Depending on the comparison chosen, this would be five orders higher (for the 10^{-13} mol m^{-1} s^{-1} Pa^{-1} stated above for polymeric membranes), and between two and four orders higher (for the 10^{-12} to 10^{-10} mol m^{-1} s^{-1} Pa^{-1} range stated above for supported molten-salt membranes). In the case of alumina-supported, molten-salt membranes, permeability would need to be about four orders higher.

Alternatively, we could consider the CO_2 driving force that would be required to achieve the fluxes we report (10^{-3} mol s^{-1} m^{-2}), assuming the previously reported state-of-the-art permeabilities, but not imposing the limitation of the input stream being at atmospheric pressure (we note that this would most likely lead to intolerable costs). In this hypothetical case, polymeric membranes would require a driving force of 4 x 10^6 Pa CO_2, and supported molten-salt membranes would require 4,000 to 4 x 10^5 Pa CO_2. We must recall that the fluxes we reported were achieved when the input stream concentration was 40 Pa CO_2 (40 Pa is approximately equal to 400 ppm at atmospheric pressure).

However, in all the cases above, significant caution is needed as water-driven uphill permeation of CO_2 is unique, and a simple permeability comparison does not capture the profound differences between downhill and uphill CO_2 permeation. For instance, a conventional definition of permeability leads to negative permeabilities when considering uphill permeation.

Moving beyond a simple permeability comparison, we also note that there is no other work in the literature that reports characterised mixing conditions alongside the measurement of the four (CO_2 and H_2O, on the two sides of the membrane), or even two (CO_2 or H_2O, on the two sides of the membrane), membrane partial pressures required for a full understanding of operation. The absence of characterisation of mixing and full partial pressure data sets in the literature makes detailed and direct comparison of our work to that literature impossible, even when apparently similar conditions are used (e.g., the same feed partial pressures).

Supplementary Note 3. Downhill experiments in dry and humidified gas streams. An example of downhill permeation experiments at 550 °C are shown in Supplementary Figure 5 with the data also contributing to Figure 2. The operating conditions of the membrane varied with time. At times t_1, t_2, t_3 and t_5 the input stream (internal chamber inlet) is 77, 90, 50 and 25% CO_2 / N_2 respectively with Ar as the sweep gas (external chamber inlet). The corresponding amount of CO_2 that permeated was 32, 34, 24 and 15 ppm respectively. At t_4, the input stream is 3.5% H_2O in 50% CO_2 / N_2 with Ar as the sweep gas. At t_7, the input stream is 50% CO_2 / N_2 with 3.5% H_2O in Ar as the sweep gas. At times t_6 and t_8 the conditions used at t_3 were repeated. The internal chamber outlet was monitored by a CO_2/H_2O IR analyser (in this case to record H_2O) and a mass spectrometer (to record CO_2) in series and the external chamber outlet was monitored by a CO_2/H_2O IR analyser.

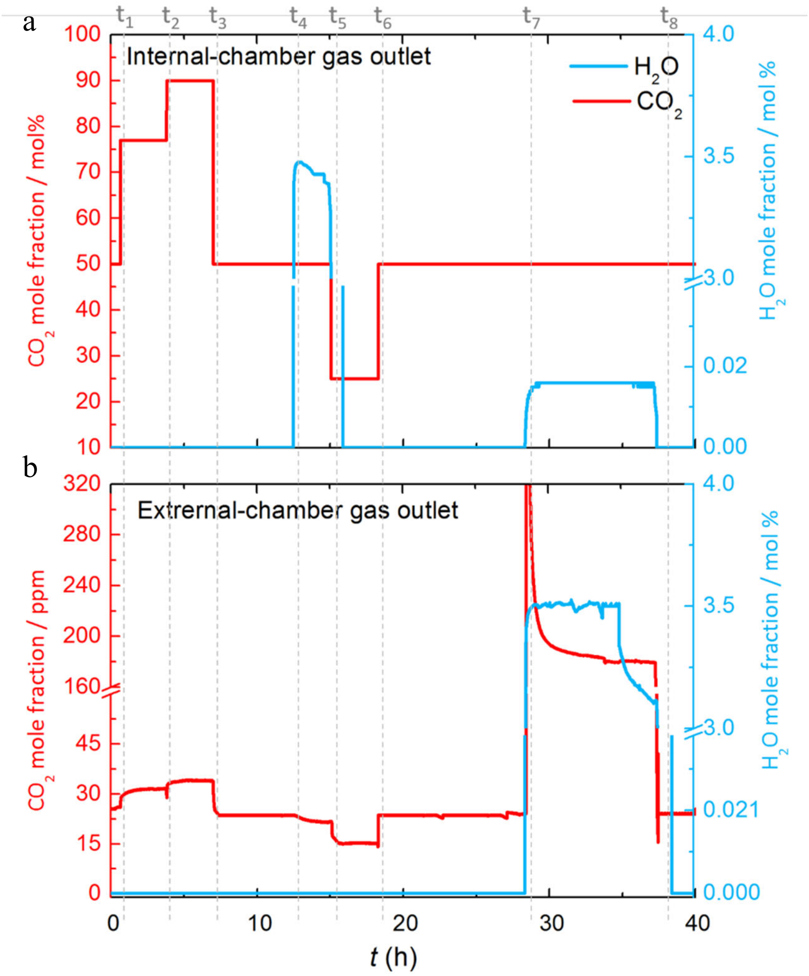

Supplementary Figure 5. Downhill experiments in dry and humidified gas streams. Mole fraction of CO_2 and H_2O in both a, internal and b, external chamber gas outlets at 550 °C. At times t_1, t_2, t_3, t_5, t_6 and t_8: input stream of 77, 90, 50, 25, 50 and 50% CO_2 / N_2 respectively and Ar sweep gas. At t_4, input stream of 3.5% H_2O in 50%CO_2 /N_2 and Ar sweep gas. At t_7, input stream of 50% CO_2 / N_2 and 3.5% H_2O in Ar sweep gas.

Supplementary Note 4. Uphill experiments in dry and humidified air streams. This experiment is presented to explain the concept of symmetrical and asymmetrical conditions during uphill experiments (Supplementary Figure 6 with the data also contributing to Figure 3a). Initially the membrane was held under symmetrical conditions (sym) with air (409 ppm CO_2 / 20% O_2 / N_2) as both input stream and sweep gas, for approximately two hours. At t_1, the sweep gas was switched to humidified air (3.5% H_2O in 409 ppm CO_2 / 20% O_2 / N_2). During symmetrical conditions the mole fraction of CO_2 is stable at 409 ppm at the input stream and output stream outlets. During asymmetrical conditions, the CO_2 concentration drops to ~170 ppm at the input stream outlet and rises to ~650 ppm in the output stream. Thus, the mass balance during asymmetrical operation closes, *i.e.*, the amount of CO_2 consumed from the input stream is equal to the amount of CO_2 in the output stream. It is also observed that ~230 ppm H_2O permeates from the sweep gas, into the input stream (measured at the input stream outlet), which gives a 1:1 (H_2O:CO_2) counter-permeation ratio. In Supplementary Figure 6 it is also observed that when humidified air is used as the sweep gas, initially the CO_2 mole fraction overshoots, while the mole fraction of CO_2 in the input stream does not show the same behaviour. Transient CO_2 evolution and consumption during gas switches is discussed in Supplementary Note 5.

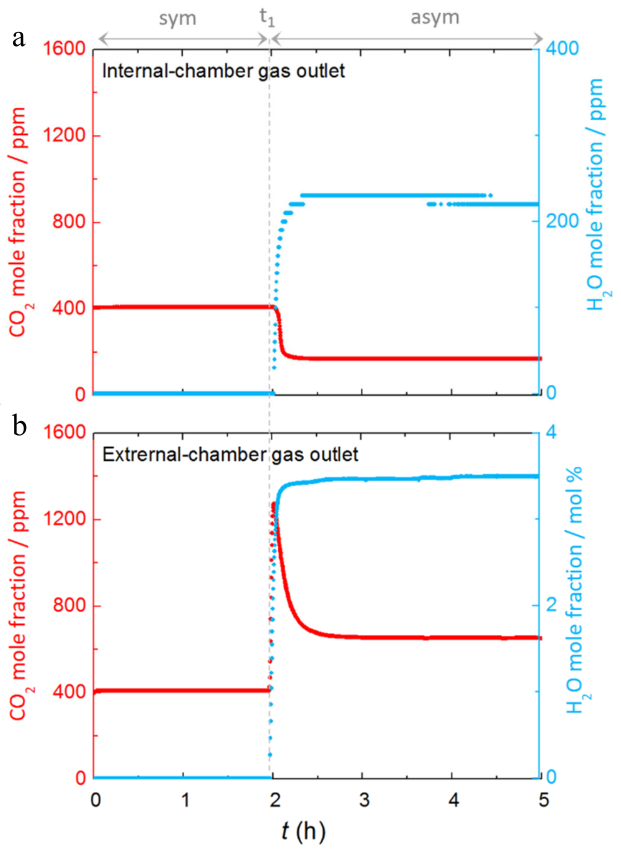

Supplementary Figure 6. Uphill experiments in dry and humidified air streams. Mole fraction of CO_2 and H_2O in both a, internal and b, external chamber gas outlets at 550 °C. Symmetrical operation (sym), where the input stream and sweep gas are both 409 ppm CO_2 / 20% O_2 / N_2.

Asymmetrical operation (asym), where the input stream is 409 ppm CO_2 / 20% O_2 / N_2 and the sweep gas is 3.5% H_2O in 409 ppm CO_2 / 20% O_2 / N_2.

Supplementary Note 5. Chemical response of the membrane upon subsequent introduction and removal of H_2O in the gas phase. It was observed that upon subsequent introduction and removal of H_2O in the gas phase, CO_2 was produced from, and consumed by, the membrane, respectively. This suggests that the composition of the melt is also changing during these transients. Supplementary Figure 7 shows the chemical response of the membrane upon subsequent introduction and removal of 3.5% H_2O in the gas phase under symmetrical (non-permeating) conditions where air was used as an input stream and sweep gas. The amount of CO_2 evolved from the melt was obtained through peak integration. The introduction and removal of H_2O was repeated three times.

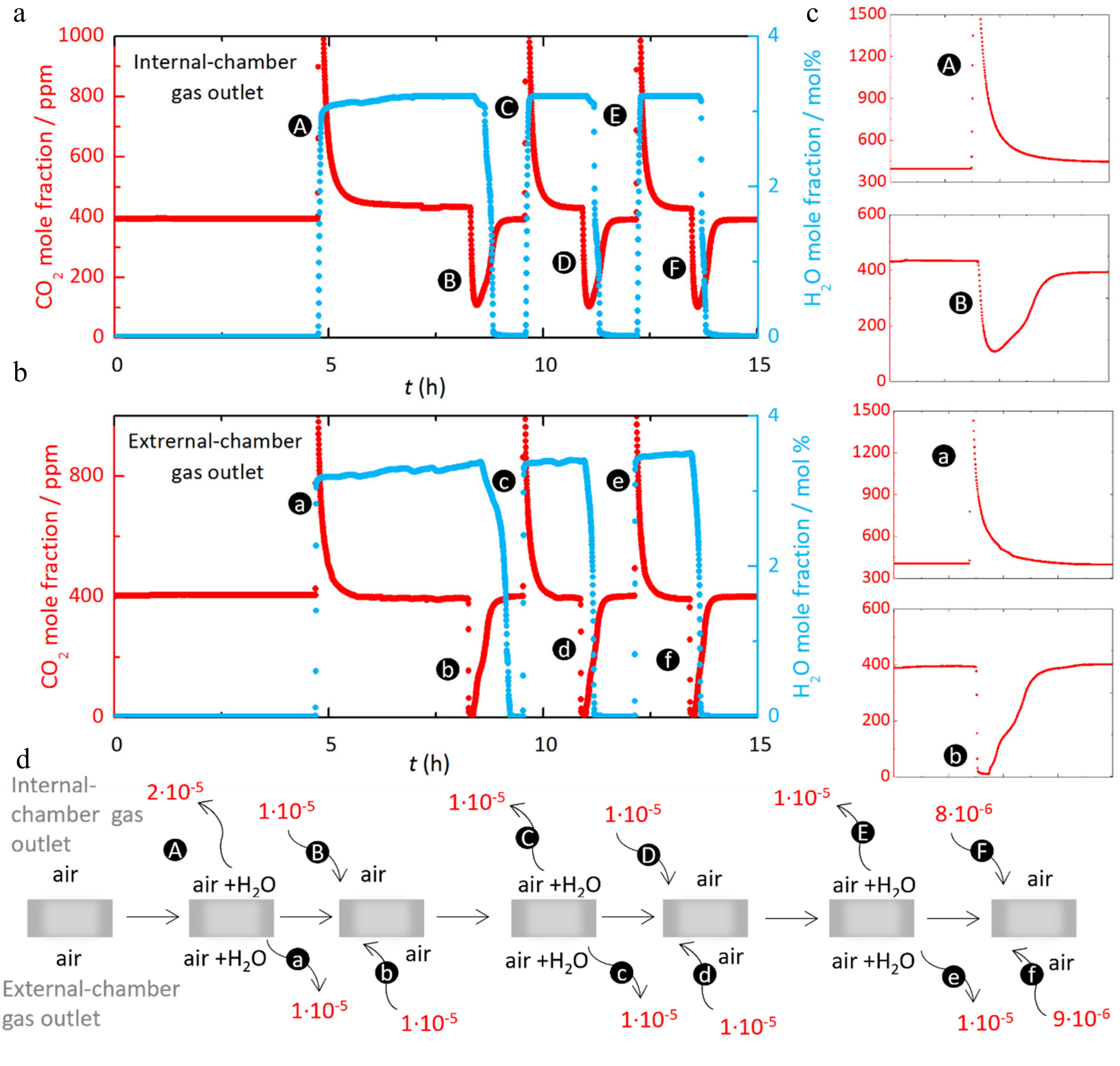

Supplementary Figure 7. Chemical response of the membrane. The mole fraction of CO_2 and H_2O, **a**, in the internal chamber gas outlet and **b**, in the external chamber gas outlet at 700 °C upon introduction and removal of H_2O in the gas phase under non-permeating conditions. **c**, Magnified view of some of the peaks of CO_2 evolution and consumption during the introduction

and the removal of H_2O respectively. **d**, Schematic of the membrane showing the moles of CO_2 evolved or consumed on both sides of the membrane upon introduction and removal of H_2O.

Supplementary Note 6. Non-equal air flow rates. The effect of non-equal air flow rates on CO_2 capture from air under uphill conditions was investigated by changing the flow rate of the sweep gas (Supplementary Figure 8 with the data also contributing to Figure 3b). Initially the membrane was held under symmetrical conditions (sym) with air (409 ppm CO_2 / 20% O_2 / N_2) as both input stream and sweep gas, for approximately one hour. At t_1, the sweep gas was switched to humidified air (3.5% H_2O in 409 ppm CO_2 / 20% O_2/ N_2). The flow rate of the input stream was held at 30 cm^3 (STP)/min, whilst the sweep gas was reduced to 6 cm^3 (STP)/min. The H_2O and CO_2 mole fractions at the input stream outlet were 210 and 190 ppm, respectively, while the product stream CO_2 mole fraction was raised to 1380 ppm. Note that the increase in CO_2 mole fraction in the product stream is five times higher than the decrease in the input stream, consistent with the ratio of the corresponding flow rates (also demonstrating an absence of mass transfer limitations with flow rate). At t_2, the membrane was again held under symmetrical conditions (sym) with air (409 ppm CO_2 / 20% O_2 / N_2) as both input stream and sweep gas, for approximately two hours. At t_3, the sweep gas was switched to humidified air (3.5% H_2O in 409ppm CO_2 / 20% O_2/ N_2). The flow rate of both the input stream and sweep gas were 30cm^3 (STP)/min. The H_2O and CO_2 mole fractions at the input stream outlet of the internal chamber gas outlet were measured to be 220 ppm and 160 ppm, respectively, while the product stream CO_2 mole fraction was raised to 640 ppm.

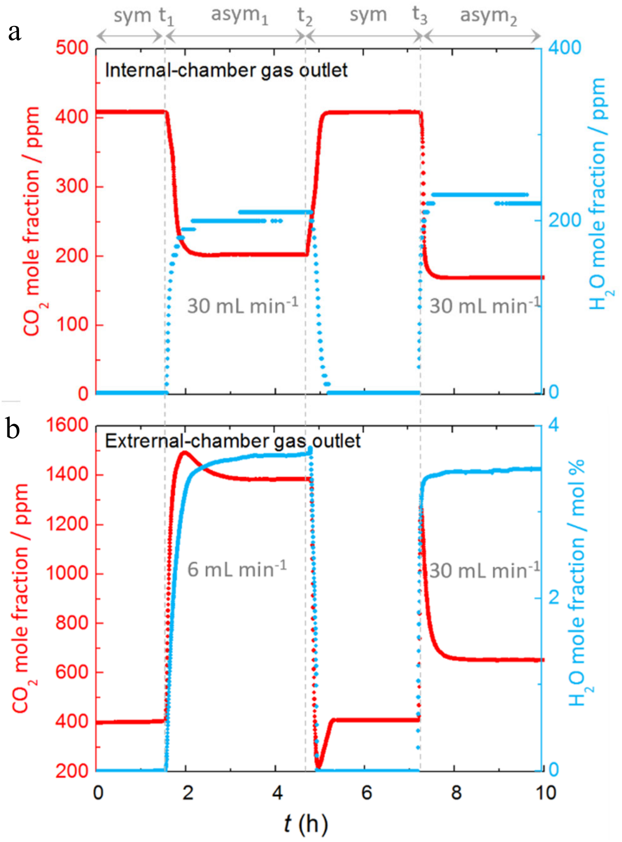

Supplementary Figure 8. Non-equal air flow rates. Mole fraction of CO_2 and H_2O in both a, internal and b, external chamber gas outlets at 550 °C. Symmetrical operation (sym), where the input stream and sweep gas are both 409ppm CO_2/20%O_2/N_2. Asymmetrical operation, where the input stream is 409ppm CO_2/20%O_2/N_2, and the sweep gas is 3.5% H_2O in 409ppm

CO_2/20%O_2/N_2, $asym_1$: 30cm^3/min input stream and 6 cm^3/min sweep gas, $asym_2$: 30 cm^3/min input stream and sweep gas.

Supplementary Note 7. Reversing inlets. Due to the difference between the total surface areas of the artificial pores on the top and bottom surface of the membrane (Supplementary Figure 1, and Supplementary Table 1), reversing the direction of CO_2 permeation can provide an indication of whether CO_2 permeation rate is limited by CO_2 consumption or CO_2 evolution into/from the melt. Supplementary Figure 9 shows that at 600 °C and above, CO_2 permeation increases when CO_2 is evolved from the greater artificial pore area (top surface). Similar results have been observed for the experiments conducted under humidified conditions (uphill and downhill) that were tested at 700 °C, and in previous literature.[5]

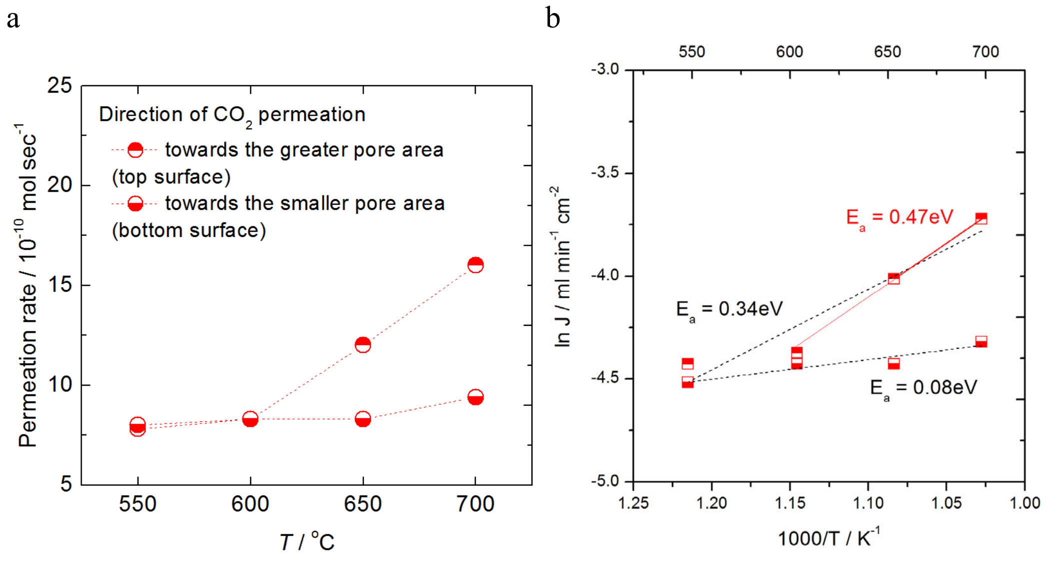

Supplementary Figure 9. Reversing inlets. a, CO_2 permeation rate with temperature for both directions of CO_2 permeation and **b**, the corresponding Arrhenius plot. The direction of CO_2 permeation is changed by swapping the input stream and sweep gas inlets (50%CO_2/N_2 and Ar). Dashed lines in **a** are guides to the eye. All lines in **b** indicate the fitting of a linear function to experimental data.

Supplementary Note 8. Supported molten-salt membrane stability and CO_2 balance under long-term operation. The membrane was tested continuously over a period of 250 h (Supplementary Figure 10). During this period, temperature, gas phase compositions, and H_2O content was varied. The membrane was operated at 550, 600, 650 and 700 °C, the input stream was varied from 25 to 90% CO_2 / N_2 and the sweep gas was Ar, 380 ppm CO_2 /Ar,, 1%CO_2 /Ar or 3.5%H_2O/Ar. Over this period, the average permeation rate measured is ~ 1×10^{-9} mol s^{-1}. The molar amount of CO_2 permeated during this time will be ~1×10^{-3} mol. If this is compared to the molar hold up of the carbonates (~0.04 g or ~4×10^{-4} mol, average molecular weight of carbonates is 100 g mol^{-1}), it can be shown that carbonate decomposition cannot account for the measured CO_2 permeation.

Comparing Data Points 1, 5 and 17 (Ar sweep gas), to 15 (439 ppm CO_2 in Ar sweep gas), and 6 and 12 (1% CO_2 in Ar sweep gas), suggests that in the absence of H_2O, the permeation rate depends upon the log ratio of CO_2 partial pressure across the membrane. Also, Data Points 1, 5, and 17 show that the permeation rate does not change when returning to the same experimental condition three times over ~five days, giving further confidence in membrane stability (and repeatability of experiments).

a

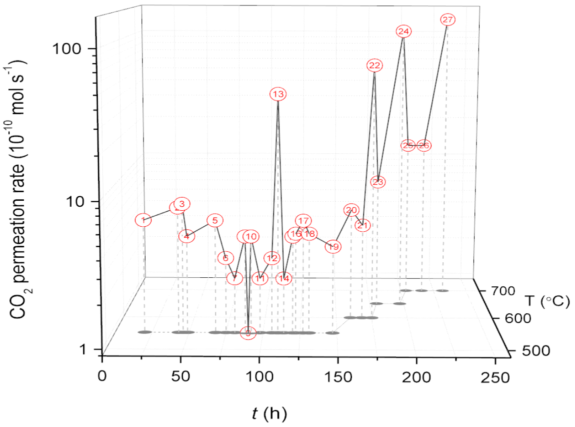

b

Data points	Internal chamber gas inlet	External chamber gas inlet
1, 5, 17, 20,23, 25, 26	50%CO_2/N_2	Ar
2	77%CO_2/N_2	Ar
3	90%CO_2 /N_2	Ar
4, 21	25%CO_2 /N_2	Ar
6, 12	50%CO_2/N_2	1%CO_2/Ar
7, 11,14	25%CO_2/N_2	1%CO_2/Ar
8	77%CO_2/N_2	1%CO_2/Ar
10	90%CO_2/N_2	1%CO_2/Ar
9	1%CO_2/N_2	1%CO_2/Ar
13, 22,24,27	50%CO_2 /N_2	3.5%H_2O/Ar
15	50%CO_2/N_2	439 ppmCO_2/Ar
16, 18	77%CO_2/N_2	439 ppmCO_2/Ar
17	90%CO_2/N_2	439 ppmCO_2/Ar
19	25%CO_2/N_2	439 ppmCO_2/Ar

Supplementary Figure 10. Supported molten-salt membrane stability. CO_2 permeation rate calculated from the CO_2 mole fraction in the external chamber gas outlet at 550, 600, 650 and

700 °C over a period of 250h. **a**, Permeation rate against time at 550, 600, 650 and 700 °C under **b**, different gas stream inlets. Lines in **a** are guides to the eye.

Supplementary Note 9. Uphill experiments in humidified gas streams with different H_2O content. In Supplementary Figure 11, uphill CO_2 permeation with humidified input stream and sweep gas is shown. The membrane was initially under symmetrical conditions (sym) with air (409 ppm CO_2 / 20% O_2 / N_2) as both input stream and sweep gas. At t_1, the input stream and sweep gas were switched to humidified air, with different H_2O concentrations (0.6% and 3.5% H_2O in 409 ppm CO_2 / 20% O_2 / N_2, respectively). The flow rate of both the input stream and sweep gas was 30 cm^3 (STP) min^{-1}. The CO_2 mole fraction at the input stream outlet was reduced to 330 ppm and, in the product stream, it was raised to 485 ppm. Again, the mass balance closes. At t_2, the membrane was returned to the initial symmetrical conditions.

Comparing this result with Supplementary Figure 6, here, the introduction of 0.6% H_2O in the input stream reduces the water ratio across the membrane from ~150 to ~5 (a factor of ×30). However, the CO_2 permeation rate drops by a factor of ×3. This suggests that the CO_2 permeation rate depends upon the log ratio of H_2O partial pressure across the membrane.

Reflecting on the uphill permeation data (Figure 3, Supplementary Figures 6 and 11, and Supplementary Table 2 etc), it appears that the CO_2 partial pressure ratio across the membrane does not have a strong effect on the permeation rate in the presence of a H_2O driving force. Therefore, under these conditions, the flux expression takes the form:

$$j_{CO_2} = \frac{\hat{P}_{M,counter,CO_2}}{l} \; ln \frac{p_{H_2O''}}{p_{H_2O'}} \qquad \text{Equation 6}$$

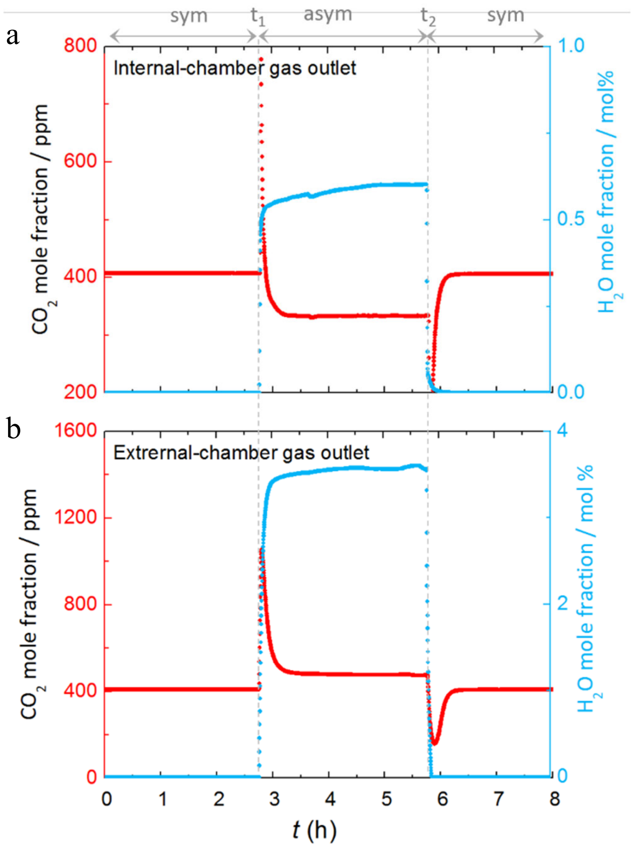

Supplementary Figure 11. Uphill experiments in humidified gas streams with different H_2O content. Mole fraction of CO_2 and H_2O in both **a**, internal and **b**, external chamber gas outlets at 550 °C. Symmetrical operation (sym), where the input stream and sweep gas are both 409 ppm CO_2/20%O_2/N_2. Asymmetrical operation (asym), where the input stream is 0.6% H_2O in 409 ppm CO_2/20%O_2/N_2 and the sweep gas is 3.5% H_2O in 409 ppm CO_2/20%O_2/N_2.

Supplementary Discussion

Molecular Density Functional Theory (DFT) Calculations

Computational Details

Solvent environment

A number of environments have been modelled (**Supplementary Figure 12**). Species denoted (gp) are evaluated in the gas-phase (**Supplementary Figure 12a**). Ions or molecules interacting on the surface are well represented by gas phase calculations of the ion or molecule with 1-2 explicit solvating ions (**Supplementary Figure 12b**). Species present on the surface can be a wide range of ions and clusters, for example: M^+, $[CO_3]^{2-}$, $[OH]^-$, $[HCO_3]^-$, $[M{\bullet}CO_3]^-$, $2M{\bullet}CO_3$, $[3M{\bullet}CO_3]^+$, $M{\bullet}OH$, $(M{\bullet}OH)_2$, and $[2M{\bullet}OH]^+$. At operational temperatures (550-700ºC) water will only be present in vapour form and a monolayer (or more) of condensed water on the surface is not expected.

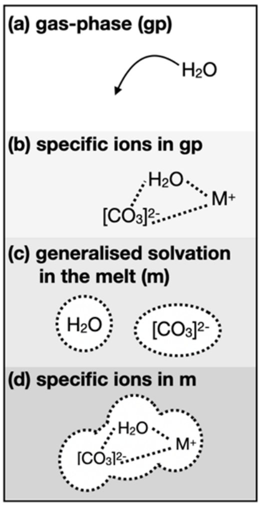

Supplementary Figure 12. Solvent environments. (a) individual species in the gas-phase, **(b)** explicitly solvated species in the gas-phase, **(c)** individual species within a generalised SMD solvation environment and, **(d)** explicitly solvated species in the generalised SMD solvation environment.

Within the bulk environment, the effects of generalised molecular solvation are provided by the continuum solvation model SMD (universal Solvation Model based on solute electron Density). Species evaluated with SMD in the melt are denoted (m) (**Supplementary Figure 12c**). Generally solute solvation with 1-2 explicit ions surrounded by a generalised solvent environment is considered a better model for a fully solvated system (**Supplementary Figure 12d**).

SMD has been developed specifically to enable consideration of solute ions in *any* condensed phase liquid medium.[6-8] Parameters employed include: static dielectric ε=3.0, optical permittivity ε_∞=2.25, Abraham's H-bond acidity α=0, Abraham's basicity β=0.99, macroscopic surface tension γ=273 cal mol^{-1} A^{-2}, halogenicity ψ=0 and the number of aromatic carbon atoms ϕ=0. A limited range of alternative parameters and atomic radii have been tested. Frequency analysis with SMD often returned larger variation in the formally zero contributions for the removal of CoM motion, even with the SCF convergence criteria set to 10^{-11} on the RMS density matrix and 10^{-9} on the energy (scf=conver=11), and optimisation convergence criteria set to $1*10^{-5}$ and $4*10^{-5}$ for the RMS forces and displacement respectively (opt=tight). Frequencies in the formally zero modes up to ±45 were accepted for the SMD level calculations only (it is normal to accept formally zero modes up to ±15 cm^{-1}).

Reaction paths

For each species there are a multitude of possible reaction paths, thus the reactions outlined herein are for selected reasonable pathways (a particular reaction will be denoted by R). An illustrative example for each of the reaction calculations outlined below is provided in **Supplementary Table 3,** for the reaction of H_2O with $[CO_3]^{2-}$ to form the adduct $[H_2O{\bullet}CO_3]^{2-}$. Reactions have been evaluated; entirely in the gas-phase R(gp) (**Supplementary Table 3a**), entirely within the melt phase R(m) (**Supplementary Table 3b**) and evaluated in a *mixed* environment R(x), where neutral gases are evaluated in the gas-phase and the anions are treated in the melt environment (**Supplementary Table 3c**)

Reaction paths are also evaluated via two different *stepwise* mechanisms that include explicit solvation. For the first path denoted stepwise(R -> solvate) in **Supplementary Table 3d**, reactants form the product adduct *in the gas phase*, and subsequently the *adduct is solvated* within the melt. For the second path denoted stepwise (solvate -> R) in **Supplementary Table 3e**, *reactants are solvated* within the melt, and subsequently form an adduct within the melt environment. These stepwise mechanisms provide an indication of the localised ΔG associated with individual reaction steps and the different impact on ΔG of specific environments. An average overall thermodynamic mechanism can be considered to be a "sum" of all the short time specific individual interactions represented within the stepwise mechanisms.

(**a**) R(gp)	$H_2O_{(gp)} + [CO_3]^{2-}_{(gp)} \rightleftharpoons [H_2O{\bullet}CO_3]^{2-}_{(gp)}$
(**b**) R(m)	$H_2O_{(m)} + [CO_3]^{2-}_{(m)} \rightleftharpoons [H_2O{\bullet}CO_3]^{2-}_{(m)}$
(**c**) R(x)	$H_2O_{(gp)} + [CO_3]^{2-}_{(m)} \rightleftharpoons [H_2O{\bullet}CO_3]^{2-}_{(m)}$
(**d**) stepwise(R -> solvate)	reaction: $H_2O_{(gp)} + [CO_3]^{2-}_{(gp)} \rightleftharpoons [H_2O{\bullet}CO_3]^{2-}_{(gp)}$<br>followed by solvation of product:<br>$[H_2O{\bullet}CO_3]^{2-}_{(gp)} \rightarrow [H_2O{\bullet}CO_3]^{2-}_{(m)}$
(**e**) stepwise(solvate-> R)	solvation or reactants: $H_2O_{(gp)} \rightarrow H_2O_{(m)}$<br>followed by reaction:<br>$H_2O_{(m)} + [CO_3]^{2-}_{(m)} \rightleftharpoons [H_2O{\bullet}CO_3]^{2-}_{(m)}$

Supplementary Table 3. **Illustrative example of the different reaction Gibbs free energies evaluated.**

Reaction energies

The following are specific details that feed into the evaluation of the reaction energies reported. Reaction energies are evaluated ΔG=G(products)-G(reactants), ΔG(R) will be reported for the melt at 550 °C unless otherwise indicated.

The focus is on thermodynamic reaction energies, ie the stability of products relative to reactants. Kinetics are assumed to be unimportant, in that the reaction barriers E_a are assumed to be small for all reactions, ie activation energies are assumed accessible. This is supported by the experimental studies undertaken here. The CO_2 excess on feed side leads to permeation rates of the order of 10^{-9} mol/s. Using E_a=-(ln(k)*RT)/1000 and a reaction rate of $1*10^{-9}$ (from the experiments) and a temperature of 823- 973 K activation energies of ≈12 kJ/mol are accessible.

When gas phase and solvated phase energies are compared, care is required in the evaluation of Gibbs free energies. Ideal gas standard state is 1mol, and a solution standard state is a 1M solution. As is standard practice, we will convert (where required) 1mol gas-phase results to a 1M solution to allow for direct comparison of energies.[9] $\Delta G^*=-T\Delta S=RT\ln(V_{gas}/V_{soln})$ is the free energy change of **1 mol of an ideal gas at 1atm to 1M in solution, evaluated at 550ºC ΔG*= +29 kJ/mol**. Where appropriate these standard state corrections are explicitly noted in the discussion: $X_{gp}\rightarrow X_{soln}$ are corrected by +ΔG* and vaporisation energies $X_{soln}\rightarrow X_{gp}$ are corrected by -ΔG* evaluated at the relevant temperature.

The solvation energy for absorbing gaseous water (+3 kJ/mol) corrected for the state change (+29 kJ/mol) gives +32 kJ/mol at 550ºC, and conversely the **Gibbs free energy for releasing water vapour is -32 kJ/mol**. The solvation energy for absorbing CO_2 (+39 kJ/mol) corrected for the state change (+29 kJ/mol) is +68 kJ/mol, and conversely for **releasing CO_2 is -68 kJ/mol**. Thus both CO_2 and H_2O would prefer to evaporate out of the melt.

For a reaction to proceed $\Delta G=\Delta G^\ominus+RT\ln Q$ should be zero or negative where Q= [P]/[R] ie the ratio of the pressure or concentration of the products (P) over the reactants (R). To reach completion we can require maximum ΔG=0 for Q=9999. Thus, for a reaction gone to completion, the standard state $\Delta G^\ominus$=-Rln(9999)*T=96.57*T difference between the products and reactions will be -22.8 kJ/mol (T=298K), and **-42 kJ/mol for T=550 ºC** or -53 kJ/mol at 700ºC. Any reaction with a more negative ΔG will go to completion. Both reactants and products will be present for reactions with ΔG between $\pm\Delta G^\ominus$, products favoured for -ΔG and reactants favoured for +ΔG between $\pm\Delta G^\ominus$ and a 50%/50% reactants/products at ΔG=0.

Computationally we evaluate $\Delta G^\ominus$, thus to obtain an overall ΔG a correction needs to be made for the concentration of H_2O and CO_2. The CO_2 feed side has CO_2 going in (and water going out), the CO_2 output side (permeate side) has CO_2 coming out (and water going in). Concentration effects are accounted for by G_{conc}=+RTln(Q) where Q= [P]/[R]. For the humid reactions, we assume water [R(H_2O)]=30,000ppm on H_2O input side, and [P(H_2O)]=200ppm on the H_2O output side, the ratio is 200/30,000=0.0067 and ln(0.0067)=-5.01. RT at 823.25K (550 ºC) is 6.84 kJ/mol. **$G_{conc}(H_2O)$**=+6.84*-5.01=**-34.27 kJ/mol.** For the CO_2 reactions [R(CO_2)]=400ppm on the CO_2 feed side and [P(CO_2)]=600ppm on the CO_2 output side, the ratio is 600/400=1.5 and ln(1.5)=+0.41. RT at 823.25K (550 ºC) is 6.84 kJ/mol. **$G_{conc}(CO_2)$**=+6.84*+0.41=**+2.80 kJ/mol.** Thus at 550 ºC the concentration correction $G_{conc}(H_2O)$=-34.3 and $G_{conc}(CO_2)$=+2.8 kJ/mol generate an overall concentration correction of the Gibbs free energy of **-31.5 kJ/mol from the CO_2 feed** to CO_2 **output side (550 ºC)**. Corrections for concentration are discussed in the text as required.

Mechanistic Explanation

Overview

Key knowledge and inferences from experimental data

- When a concentration gradient of CO_2 from high to low concentration is present, CO_2 passes through at a rate of 10^{-9} mol s^{-1}, ***thus CO_2 can permeate the membrane***
- H_2O (3.5%) on the CO_2 feed side does not affect the CO_2 permeation rate and no H_2O permeated. This means it is ***not just a surface-H_2O facilitated uptake of CO_2.***
- H_2O (3.5%) introduced on the CO_2 permeate side, increased the permeation rate by an order of magnitude to 10^{-8} mol s^{-1} and water counter-permeation occurred in a 1:1 (H_2O:CO_2) ratio
- A water concentration gradient ***means there is some water absorbed into the melt***
- CO_2 permeation enhancement occurs only when there is a higher concentration of H_2O on the CO_2 permeate side, this means a ***water concentration gradient is required***.
- The water concentration gradient provides the energy to enable the CO_2 to be transported up-hill, under the experimental conditions, the $G_{conc}(H_2O)$=-34.27 and the $G_{conc}(CO_2)$=+2.80 kJ/mol. 34.37/2.80=12, ie there is enough energy released to deliver a maximum of 12 CO_2 molecules per molecule of H_2O. ***thus one could expect a more than 1:1 ratio***.
- We postulate that the 1:1 relationship indicates the process is not just reliant on the energy supplied, but is also ***dependent on a direct 1:1 interaction of water and CO_2***
- For ***every molecule of H_2O or CO_2 absorbed a molecule is released*** (±32, ±68 kJ/mol respectively), thus there is no net energy cost.
- The reaction proceeds under the relevant concentration conditions, ***thus the total energy required to surmount reaction barriers does not exceed that released internally plus that delivered by the net concentration gradient of -31.5 kJ/mol*** (from the CO_2 feed to permeate side at 550 ºC).
- The mechanism by which water, at the molecular scale, facilitates CO_2 transport is unclear, the ***impact of water could be in the permeation process and/ or water could be required to facilitate the release of CO_2 on the permeate side.***
- H_2O or CO_2 do ***not need to remain as molecular*** species in the melt
- There is ***experimental verification of the presence of [OH]- ions*** in the melt, however there is no strong evidence for pyrocarbonate, $[C_2O_5]^{2-}$ or bicarbonate $[HCO_3]^{-}$ in the bulk environment. This does not mean these species do not exist, ***transient formation is possible within the melt and/or on the surface***.
- After changing the conditions of the experiments, there are significant oscillations and it takes some time to settle into a steady state

In summary, given the macroscopic reaction proceeds we are interested in the molecular scale mechanism, we assume: (i) no significant reaction barriers and thus reaction thermodynamics are dominating, (ii) that a 1:1 (H_2O:CO_2) ratio is required at "entry" and "exit, (iii) that CO_2 and H_2O do not need to remain as molecular species, and (iv) that transient species can exist that have not yet been experimentally observed.

Introduction to the Mechanism

Our DFT studies have shown a mechanism that can be summarized in the simplified diagram in **Supplementary Figure 13** below. Here the red (CO_2•M_nCO_3), blue (M_nCO_3•H_2O) and purple species (CO_2•M_nCO_3•H_2O) *represent more than one chemical structure/cluster.* The mechanism is complex due to the almost identical stability of a range of chemical structures. The collective purple species (**A**) represent a chemical "handshake" between CO_2 and H_2O. The collective red species represent the CO_2 carriers ($\mathbf{C_{CO_2}}$) within the melt and the collective blue species represent the H_2O carriers ($\mathbf{C_{H_2O}}$) within the melt. The specific chemical species are described in more depth below in **Supplementary Figure 16**.

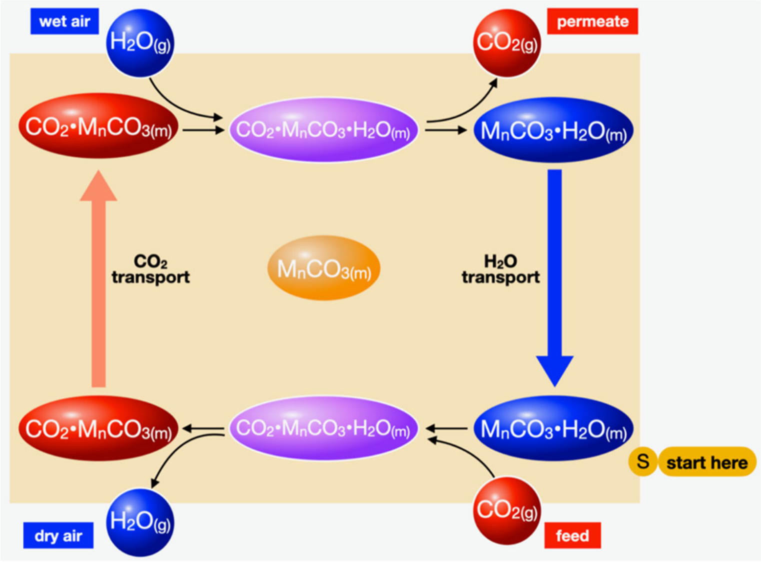

Supplementary Figure 13. Simplified CO_2 and H_2O transport mechanism in molten carbonate.

In a simple mechanism, the chemical bonds of the CO_2 or H_2O are not broken and adduct clusters form. The "•" symbol represents the formation of an adduct, where the chemical species retain their molecular structure and non-covalently interact with each other. In this simplified mechanism, starting on the bottom right and moving clockwise around the mechanism: CO_2 is absorbed into the melt and reacts with a water carbonate species (blue) to form a mixed cluster hand-shake species (purple). Subsequently water dissociates from the purple cluster to form the red CO_2 carbonate species (CO_2 carrier, $\mathbf{C_{CO_2}}$) which diffuses through the melt to the permeate side. Near the surface the red species reacts with water to form another mixed cluster (purple) which subsequently loses CO_2 to form the blue water carbonate species (H_2O carrier, $\mathbf{C_{H_2O}}$). The blue species then diffuses through the melt to the feed side, picking up a CO_2 molecule and continuing the cycle. Overall the concentration gradient of water drives the H_2O transport, releasing energy which is used to facilitate the slightly up-hill transfer of CO_2 from the feed to permeate side.

Details of the Mechanism

Using the DFT methodology outlined above, we can establish the specific and detailed geometric structure and energy of each of the "red", "blue" and "purple" species or clusters. In a typical reaction one species will have a clear lowest energy. However, in this case a range of species with different structures and conformers can be considered to be in equilibrium (they have almost identical low energies). For example:

- the purple mixed cluster includes, [CO_2•M_nCO_3•H_2O] **A** and the adduct species due to dissociation of water to form [OH•M_n•HCO_3•CO_2] **A"**, or association of CO_2 and CO_3 to form pyrocarbonate [H_2O•$M_nC_2O_5$] **A'** as shown in **Supplementary Figure 14(a)**
- the red CO_2 carrier carbonate species include, [CO_2•M_nCO_3] **C_{CO_2}** and the pyrocarbonate [H_2O•$M_nC_2O_5$] **C'_{CO_2}** as shown in **Supplementary Figure 14(b)**
- the blue H_2O carrier carbonate species include, [M_nCO_3•H_2O] **C_{H_2O}** which dissociates water forming hydroxide and bicarbonate [OH•M_n•HCO_3] **C'_{H_2O}** as shown in **Supplementary Figure 14(c)**

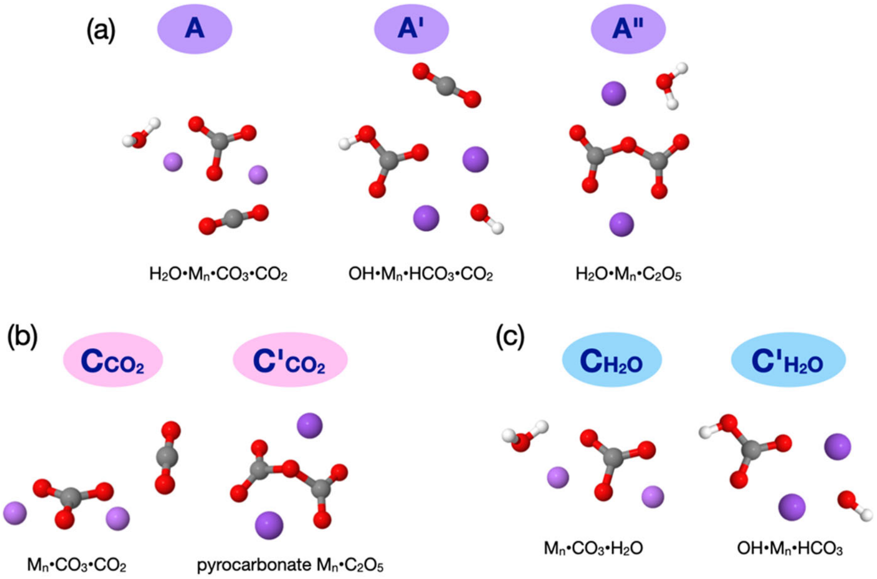

Supplementary Figure 14. Examples of the hand-shake and carrier cluster species. In **(a)** mixed clusters, **(b)** CO_2 carrier carbonate species and, **(c)** H_2O carrier carbonate species, atoms are in standard colours, C grey, H white, O red, alkali metal M purple.

Different "M_nCO_3" species can stabilise individual adducts leading to different preferred minimum energy structures. For example, each $[CO_3]^{2-}$ dianion can be closely coordinated by 1, 2 or 3 metal cations leading to negative, neutral, or cationic clusters (immersed in the general melt environment), as shown in **Supplementary Figure 15**. Moreover the metal cation can be any of (Li, Na, K), we have focused the investigation around species where the metal cation is the same, ie Li_3 and not Li_2Na

- we can have single metal association $[M•CO_3]^-$
- bimetallic association M_2•CO_3
- trimetallic association $[M_3•CO_3]^+$

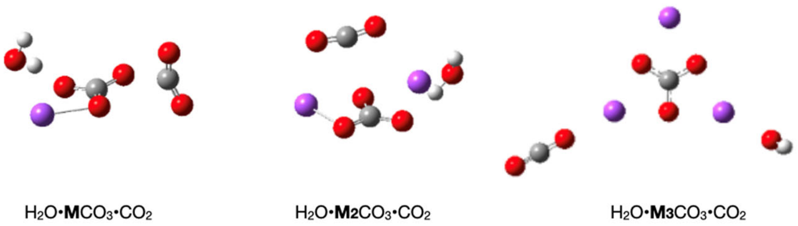

Supplementary Figure 15. Examples of different stable conformers of [CO_2•M_nCO_3•H_2O] A formed with M, M_2 and M_3.

Here we have identified the Z carrier species introduced in the main text as $\mathbf{C_{CO_2}}$ CO_2•M_nCO_3 (and $\mathbf{C'_{CO_2}}$) as the carrier for H_2O and $\mathbf{C_{H_2O}}$ M_nCO_3•H_2O (and $\mathbf{C'_{H_2O}}$) as the carrier for CO_2. In addition to the carrier species Z, a single molecular hand-shake species "A" (CO_2•M_nCO_3•H_2O) that includes both H_2O and CO_2 in a 1:1 ratio, forms as a transient intermediate.

Thus a very wide range of structures and conformers has been examined using DFT calculations, the individual species are documented in **Section 3**. From the information obtained a detailed understanding of the interconnection between species has been built up, which is summarised in **Supplementary Figure 16** below which is essentially the same as **Figure 4a** in the main paper, but now with some of the numerical data, for the 2Na cluster species (blue) in **Figure 4b** of the main paper and **Supplementary Figure 18** below.

Arrows (grey and black) indicate structures that can interconvert. The black arrows of the inner ring represent the most basic mechanism, where no covalent bonds have been broken and simple adducts are formed. This mechanism as already been described above and represented in **Supplementary Figure 13**. The grey arrows of the outer ring represent a mechanism where individual species have undergone chemical reactions, breaking covalent bonds (and forming more stable species).

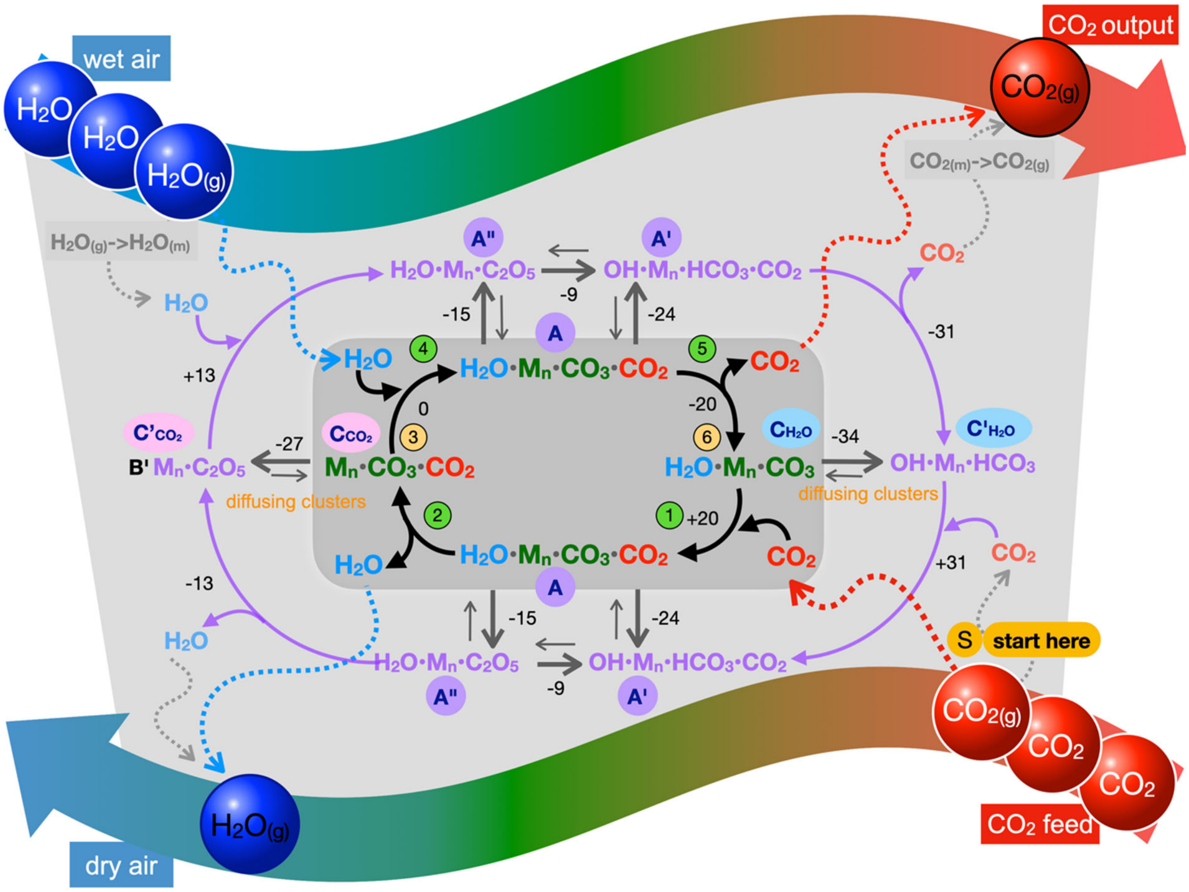

Supplementary Figure 16. **CO_2 and H_2O transport in molten carbonate**. The energy values (kJ mol^{-1}) are those for the 2Na cluster species (blue in **Figure 4b** of the main paper).

We start on the bottom right and moving clockwise around the mechanism (similar to the description for **Supplementary Figure 13**). CO_2 is absorbed into the melt and reacts with a water carbonate species [OH•M_n•HCO_3] **C'_{H_2O}** to form a mixed cluster [HCO_3•M_n•OH•CO_2] **A'** which is in equilibrium with the pyrocarbonate [M_n•C_2O_5•H_2O] **A"** species (and **A**). Subsequently water dissociates from **A"** to form the pyrocarbonate [M_n•C_2O_5] **C'_{CO_2}** (which is also in equilibrium with **C_{CO_2}**). **C'_{CO_2}** can diffuse through the melt to the permeate side. Near the surface **C'_{CO_2}** reacts with water to form the adduct **A"** [M_n•C_2O_5•H_2O] which is in equilibrium **A'** (and **A**). **A'** subsequently loses CO_2 to form [HCO_3•M_n•OH] **C'_{H_2O}** which diffuses through the melt to the feed side due to a concentration gradient in H_2O. The cycle is now complete as **C'_{H_2O}** absorbs CO_2. **Supplementary Figure 17** below, which is the same as **Figure 4b** in the main paper, provides a structural representation of the two cycles (inner and outer), for a [M_2CO_3] carbonate cluster. Information for the [M•CO_3]$^-$ and [M_3•CO_3]$^+$ carbonate clusters is provided in **Section 3**.

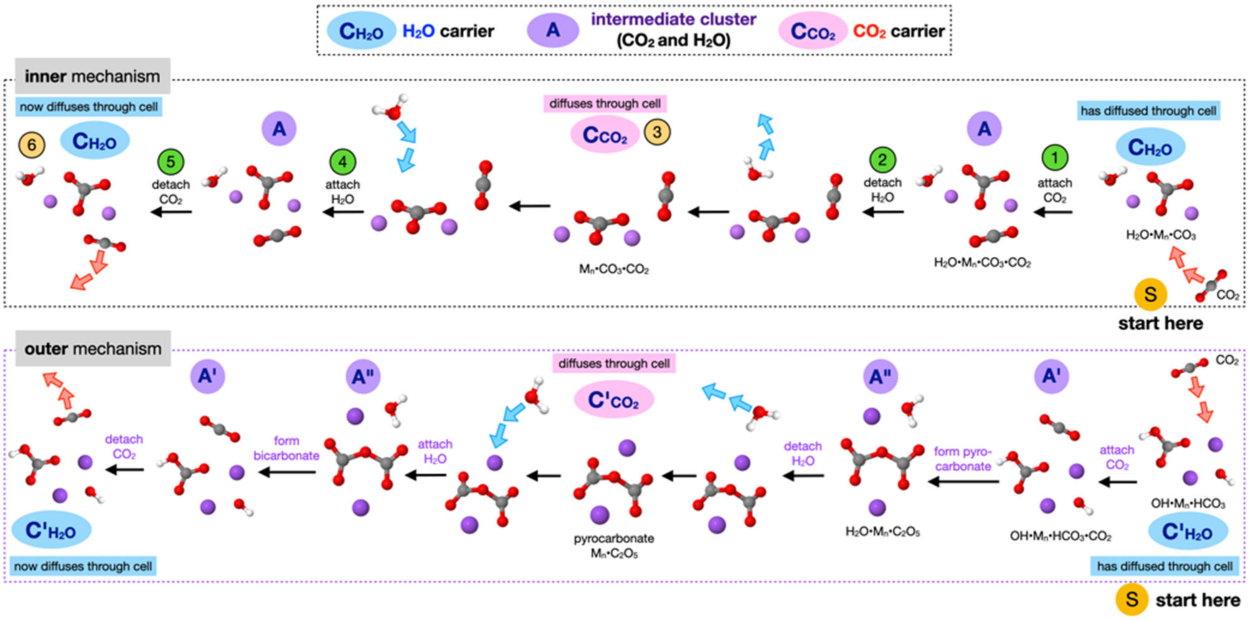

Supplementary Figure 17. **Structural representation of the inner and outer mechanisms.**

The energy of the inner and outer species determines which mechanism will dominate. **Supplementary Figure 18** shows the energy profile for CO_2 permeation for the $Na_2{\bullet}CO_3$ system evaluated in the melt at 550ºC. This information is also contained in **Figure 5a** of the paper (**Figure 5a** also contains information for the $Li_2{\bullet}CO_3$ system). The reference state is that for the individual isolated species, eg G(reference state) $=G(CO_2)_m+G(H_2O)_m+G(Na_2{\bullet}CO_3)_m$. For **Supplementary Figure 18** below the inner mechanism is the dashed line and the outer mechanism is the solid line.

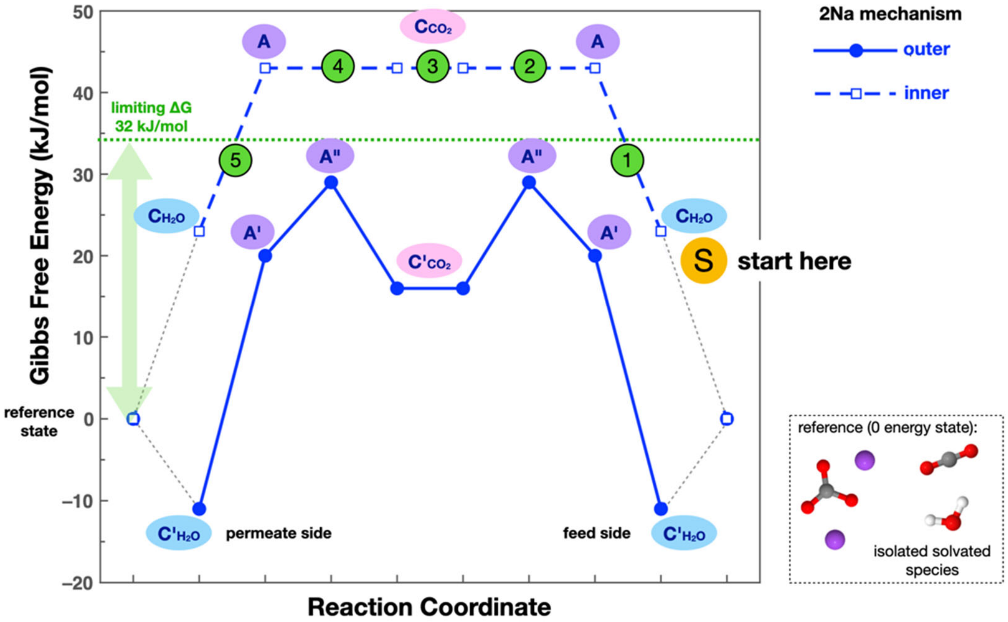

Supplementary Figure 18. ΔG (kJ mol^-1^) diagram for the inner and outer mechanisms for the $Na_2{\bullet}CO_3$ system. Lines are guides to the eye.

In **Fig S18**, following the cycle represented by the numbers and the letter S (in green/orange circles), for the inner mechanism of Na_2•CO_3 carbonate. Starting point is the carrier (blue) H_2O•Na_2•CO_3 (**C_{H_2O}**) which is +23 kJ/mol above the reference state. (1) CO_2 associates with H_2O•Na_2•CO_3 (**C_{H_2O}**) to form the purple hand-shake adduct H_2O•Na_2•CO_3•CO_2 (**A**) at a cost of +20 kJ/mol. However, there is no energy cost to step (2), where H_2O is released and the (red) cluster (**C_{CO_2}**) M_2•CO_3•CO_2 is formed. In (3) **C_{CO_2}** moves through the melt to the permeate side and in (4) **C_{CO_2}** picks up water forming **A** again. In (4) releasing CO_2 and formation of **C_{H_2O}** is downhill -20 kJ/mol. The reaction profile is necessarily symmetric, CO_2 is absorbed on one side, transported through the melt, and released on the permeate side. The energy required (limiting ΔG) as input is +43 kJ/mol.

In contrast to the inner mechanism, the formation of the carrier **C'_{H_2O}** is -11 kJ/mol relative to the reference state. However, **C'_{CO_2}** grabbing a CO_2 molecule to form **A'** is more energy expensive. In the outer mechanism going from **C'_{H_2O}** → **A'** is +31 kJ/mol, while in the inner mechanism **C_{H_2O}** → **A** is +20 kJ/mol. Nevertheless, due to the stability of **C'_{H_2O}**, **A'** lies lower in energy than **A** (relative to the reference). Energy released in the formation of **C'_{H_2O}** will be available in the system, hence it is the ΔG relative to the reference state that defines the limiting ΔG. The ΔG required to go from the reference state→ **A** is the limiting (largest) ΔG of the inner cycle. The ΔG to go from the reference state → **A"** is the limiting ΔG of the outer cycle. The energy required (limiting ΔG) as input is +29kJ/mol.

	Reaction	ΔΔG					
		1Li$^+$	1Na$^+$	2Li$^+$	2Na$^+$	3Li$^+$	3Na$^+$
	inner reactions						
ref ⇌ C_{H_2O} +CO_2	M_nCO_3+CO_2+H_2O ⇌ H_2O•M_n•CO_3+CO_2	+8	+1	+11	+23	-6	-11
C_{H_2O} + CO_2 ⇌ A	H_2O•M_n•CO_3 + CO_2 ⇌ H_2O•M_n•CO_3•CO_2	+68	+31	+23	+20	+27	+15
A ⇌ C_{CO_2}+H_2O	H_2O•M_n•CO_3•CO_2 ⇌ H_2O + M_n•CO_3•CO_2	-39	-13	-16	0	+13	+4
	**limiting ΔG**	+76	+32	+34	+43	+40	+19
	equilibria from central to outer species						
C_{H_2O} ⇌ C'_{H_2O}	H_2O•M_n•CO_3 ⇌ HO•M_n•HCO_3	-49	-63	-49	-35	+65	+46
A ⇌ A'	H_2O•M_n•CO_3•CO_2 ⇌ HO•M_n•HCO_3•CO_2	-85	-24	-44	-24	+68	+64
A ⇌ A"	CO_2•CO_3•M_n•H_2O ⇌ C_2O_5•M_n•H_2O	-47	-25	+8	-15	+132	+86
C_{CO_2} ⇌ C'_{CO_2}	M_n•CO_3•CO_2 ⇌ M_n•C_2O_5	-52	-35	+29	-27	+141	+109
	bicarbonate average	-67	-44	-46	-29	+66	+55
	pyrocarbonate average	-49	-30	+18	-21	+136	+97
	outer reactions						
ref ⇌ C'_{H_2O} +CO_2	M_nCO_3+CO_2+H_2O ⇌ HO•M_n•HCO_3+CO_2	-42	-61	-38	-11	+58	+50
C'_{H_2O} +CO_2⇌A'	HO•M_n•HCO_3 + CO_2 ⇌ HO•M_n•HCO_3•CO_2	+33	+70	+27	+31	+31	+34
A'⇌A"	HO•M_n•HCO_3•CO_2 ⇌ C_2O_5•M_n•H_2O	+38	-1	+52	+9	+64	+22
A"⇌ C'_{CO_2}+H_2O	C_2O_5•M_n•H_2O ⇌ C_2O_5•M_n+ H_2O	-43	-23	+5	-13	+85	+27
	**limiting ΔG**	+29	+8	+41	+29	+153	+106

Supplementary Table 4. Gibbs free reaction energies (kJ mol^{-1}) at 550 °C. Shaded region is for 2Na$^+$ clusters (depicted in **Supplementary Figure 13**). The blue text indicates individual steps with |ΔG| < 32 kJ/mol.

The ΔG available from the water concentration gradient is **-31.5 kJ/mol**, given by the pale green arrow and dotted green line on **Fig S18**. Thus the inner cycle (simple adduct mechanism) is not accessible for Na_2CO_3 but the outer cycle (chemical reaction mechanism) is accessible.

Supplementary Table 4 presents the energies for each stage of the mechanism for all the clusters studied $M_n(CO_3)$ where n=1-3 and M=Li^+ and Na^+ (the shaded column gives the numbers for the Na_2•CO_3 system used as an example in **Supplementary Figure 18**). Graphs the inner and outer cycles for each system studied are given in **Supplementary Figures 19** and **20** respectively. **Supplementary Figure 19** allows comparison of the inner mechanism between the different $M_n(CO_3)$ systems M=Li, Na and n=1,2,3, and similarly **Supplementary Figure 20** compares the outer mechanisms.

The limiting ΔG Gibbs free energies for the inner and outer cycles are not always for the formation of **A** or **A"**, in some cases it is the formation of $\mathbf{C_{CO_2}}$. The limiting ΔG for each reaction is given in **Supplementary Table 4**, red values are inaccessible, green values are accessible, and orange values are borderline. In **Supplementary Figures 19.** and **20** the accessible region (0-32 kJ/mol) is shaded light grey. From this data it is evident that the inner cycle is accessible for all M_nCO_3 except with 3Li^+ or 2Na^+ cations. The outer cycle is accessible for 1Li^+, 1Na^+ and 2Na^+ cation species.

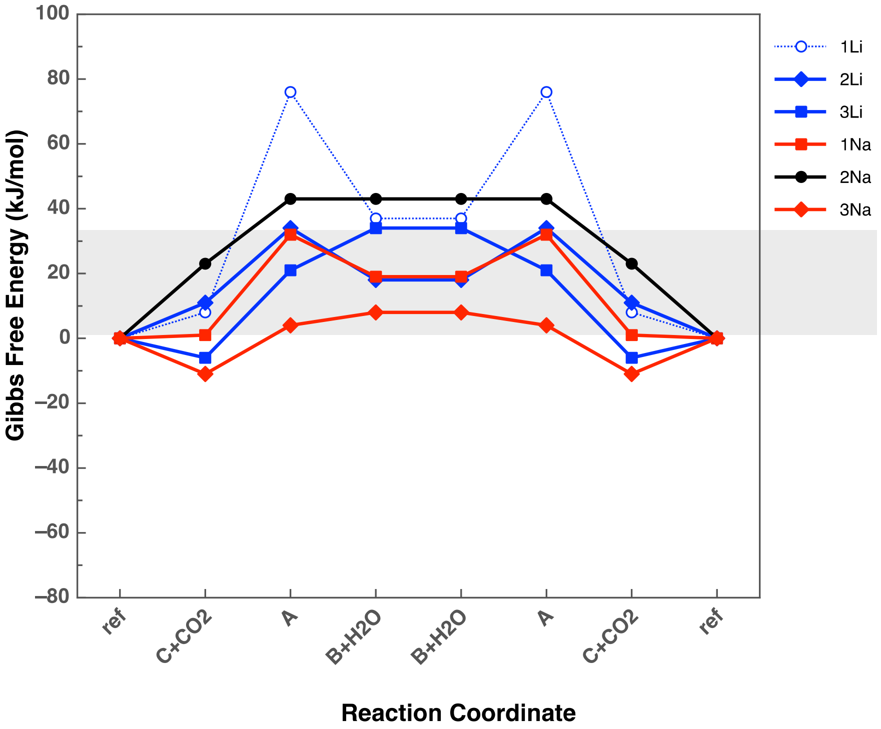

Supplementary Figure 19. Inner reaction mechanism profile for M = Li/Na and n = 1, 2, 3. Gibbs free energy in kJ mol^{-1}, relative to $M_nCO_3 + CO_2 + H_2O$. Shaded grey area is the accessible energy region due to the concentration gradient. Lines are guides to the eye.

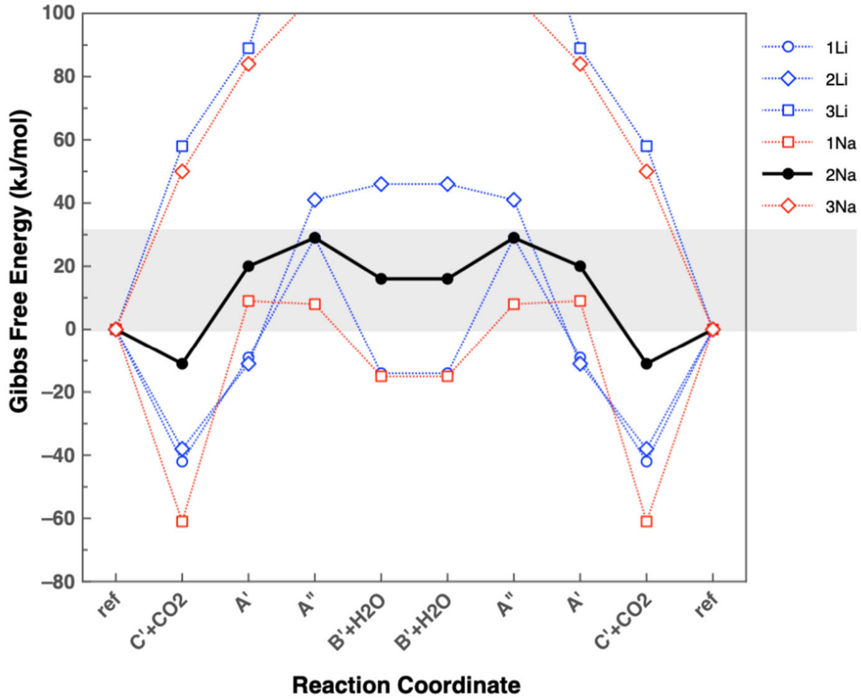

Supplementary Figure 20. Outer reaction mechanism profile for M = Li/Na and n = 1, 2, 3. Gibbs free energy in kJ mol-1, relative to $M_nCO_3 + CO_2 + H_2O$. Shaded grey area is the accessible energy region due to the concentration gradient. Lines are guides to the eye.

It is generally accepted that the hydroxide but not pyrocarbonate or bicarbonate species are present in a melt. However, the calculations undertaken here indicate that pyrocarbonate $\mathbf{C'_{CO_2}}$ and bicarbonate species $\mathbf{C'_{H_2O}}$ can form and are critical to the reaction mechanism. Bicarbonate (and hydroxide) formation have been experimentally identified in ternary alkali metal carbonate melts equilibrated with water under the introduction of CO_2. Bicarbonate has been suggested to dominate below 450ºC and hydroxide dominate above 550ºC, the intermediate region is expected to exhibit a mix, with a ratio of $[HCO_3]^-/[OH]^-$ estimated to be ≈0.42 at 500ºC for a (pure) Na_2CO_3 melt.[10,11] In some cases $[HCO_3]^-$ can be the dominant proton carrier.[12,13]

Care should be taken not to over emphasise electrochemical oxidation/reduction reactions (which are far better studied) relative to the equilibrium reactions occurring within a non-conductive membrane. Moreover excess oxygen (O^{2-}) is not present (as in solid oxide fuel cells).

The formation of a hydroxide only carrier species is possible, $\mathbf{C'_{H_2O}} \rightleftharpoons \mathbf{C''_{H_2O}}$ $HCO_3{\bullet}M_n{\bullet}OH \rightleftharpoons M_n(OH)_2{\bullet}CO_2$, n=2,3 however, for n=1 $M{\bullet}(OH)_2{\bullet}CO_2$ is unstable, **Supplementary Table 5**. There is a significant cost in energy to form hydroxide only species for M_2 but much less so for M_3 particularly for $3Li^+$ where the equilibrium is facile (+2 kJ/mol). *Thus the contribution of Li^+ ions in the melt is important*. At the surface (gas-phase) hydroxide formation is slightly more favourable but the energy is still high relative to the reference state.

$C'_{H_2O} \rightleftharpoons C''_{H_2O}$	$1Li^+$	$1Na^+$	$2Li^+$	$2Na^+$	$3Li^+$	$3Na^+$
melt	-	-	+41	+50	+2	+22
gas-phase	-	-	+25	+44	-10	-28

Supplementary Table 5. Gibbs free reaction energies (kJ mol^{-1}) at 550 °C.

The formation of a bicarbonate species occurs in the "outer" mechanism. **Supplementary Figure 20** (C'+CO_2 step) and **Supplementary Table 4** show that the outer mechanism has stable "resting states" (ΔG <-38 kJ/mol) for $[HCO_3]^-$ for 1Li, 2Li and 1Na. Moreover, $[HCO_3]^-$ is unstable for the 3M clusters (ΔG>50 kJ/mol). The energy for the higher energy processes could be supplied by other reactions within the melt. Thus, only the 2Na cluster is left as a possible outer mechanism (thick black line in **Supplementary Figure 20**) able to *both bind and release* the $[HCO_3]^-$ at moderate Gibbs free energies. Most of the systems studied here favour the inner path, shown as bold lines in **Supplementary Figure 19**.

Nevertheless, these results, combined with the experimental evidence outlined above indicate that bicarbonate (and pyrocarbonate) can be present and active in the melt. Making a prediction based on these results, there is potential to increase the "active" presence of bicarbonate by increasing the Na^+ concentration in the melt.

The exact and relevant species are very difficult to characterise, especially at the very low concentrations and high temperatures found in operational devices. The pyrocarbonate and bicarbonate are expected to be transient species in a high temperature regime (>500ºC). The proton in $[HCO_3]^-$ can easily rapidly transfer between $[CO_3]^{2-}$ ions. A similar process could occur for the CO_2 in C_2O_5 where the exchange CO_2•CO_3•M_n•H_2O ⇌ C_2O_5•M_n•H_2O for 2Li was found to be only +8kJ/mol, **Supplementary Table 4**.

Supplementary Figure 21. summarises key information for the reaction profiles for each M_nCO_3 system, and is the same as that given in **Figure 5b** of the paper. The solid dark blue line shows the inner (simple adduct) cycle limiting ΔG, demonstrating that the inner adduct cycle is only accessible for M_nCO_3 clusters of *$1Na^+$ and $3Na^+$, and is borderline for $2Li^+$ cations*. The outer cycle, light blue solid line, is accessible for $1Li^+$, $1Na^+$ and $2Na^+$ cation clusters. However $1Li^+$ and $1Na^+$ form particularly stable pyrocarbonate **C'_{CO_2}** and bicarbonate species **C'_{H_2O}**, also called "resting states" which will retard a reaction. Localisation of energy into the specific coordinates required to facilitate a large single reaction step is unlikely. Thus, while the limiting ΔG is low, the inner mechanism is unlikely to facilitate transport due to the retarding nature of the resting states. This leaves *$2Na^+$ the most likely route followed by the outer mechanism.*

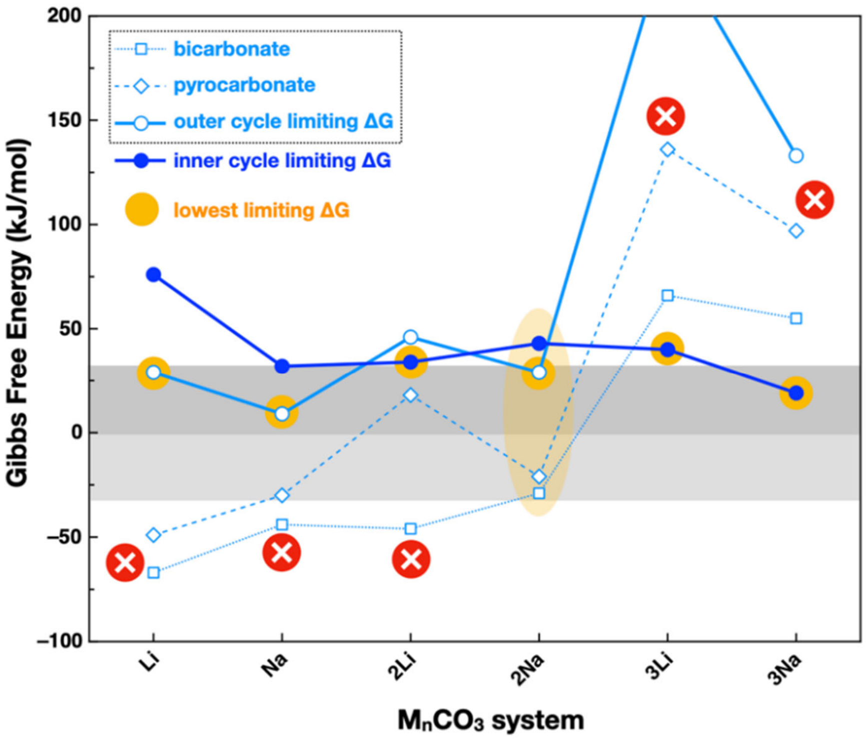

Figure. S21. Specific reaction Gibbs free energies by species. Shaded grey area is the accessible energy region due to the concentration gradient. Lines are guides to the eye.

Individual Data

Species A, A' and A", conformers based on H_2O-M_n-CO_3-CO_2 (M=Li, Na, n=1,2,3)

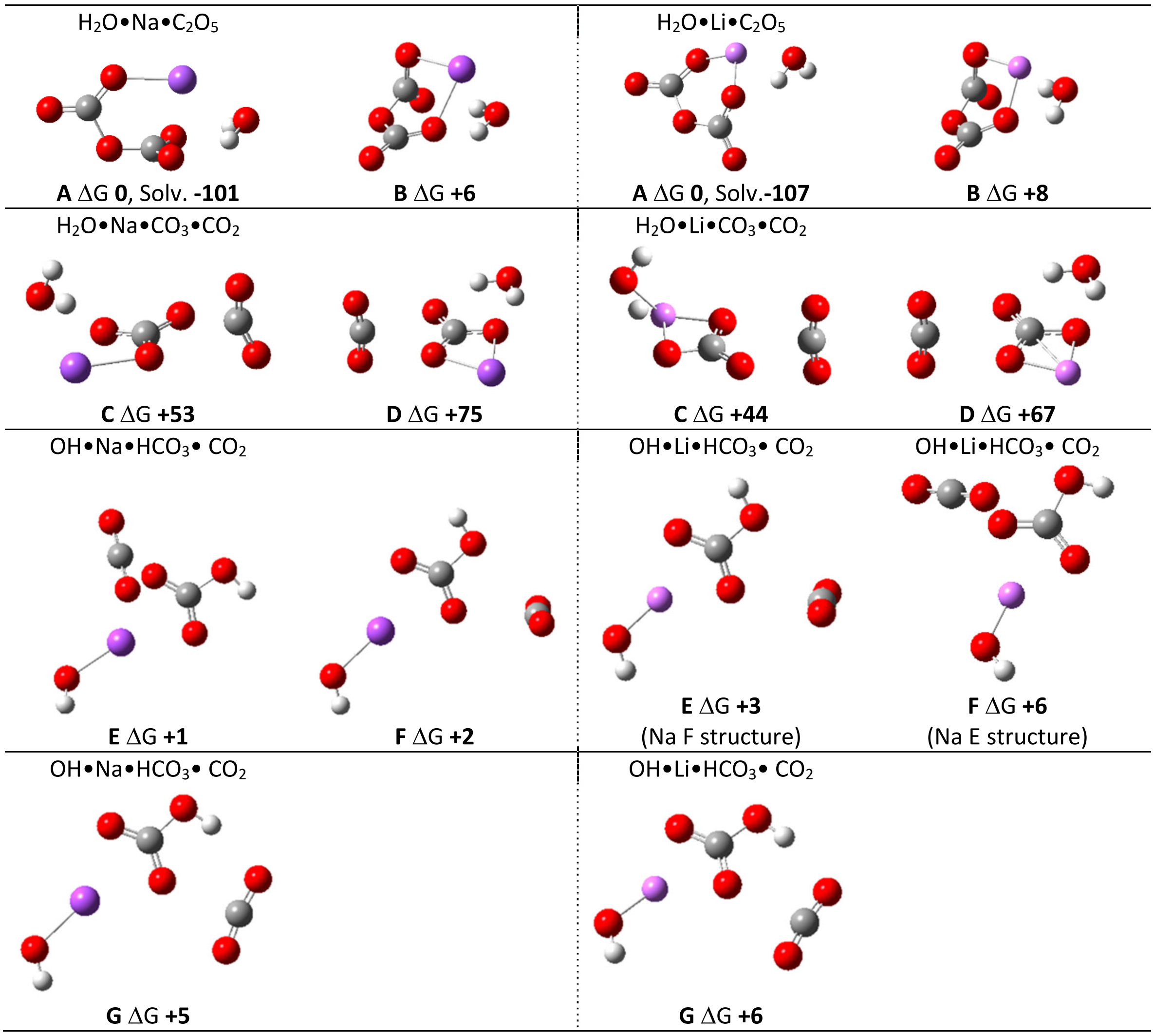

Supplementary Figure 22. Conformers A: H_2O•M_n•CO_3•CO_2 and A': HO•M_n•HCO_3•CO_2 M_n where M=Li or Na and n = 1. ΔG relative Gibbs free energies in the gas-phase to the lowest energy conformer. Solv. are solvation Gibbs free energies for the melt. Both Gibbs free energies in kJ mol^{-1} at 25 °C.

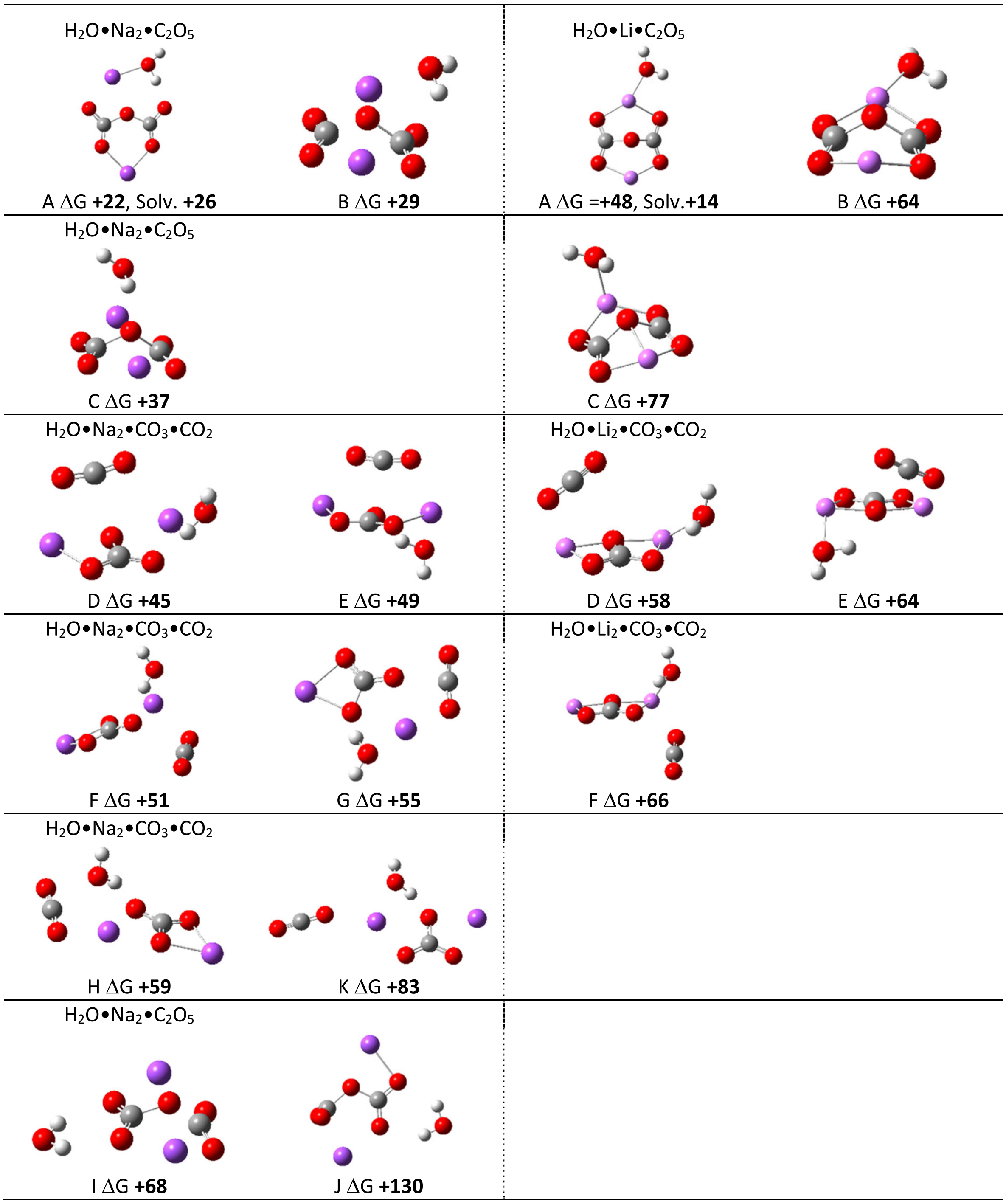

Supplementary Figure 23. Conformers A": C_2O_5•M_n•H_2O and related where M=Li or Na and n = 2. ΔG relative Gibbs free energies in the gas-phase to the lowest energy conformer. Solv. are solvation Gibbs free energies for the melt. Both Gibbs free energies in kJ mol^{-1} at 25 ºC.

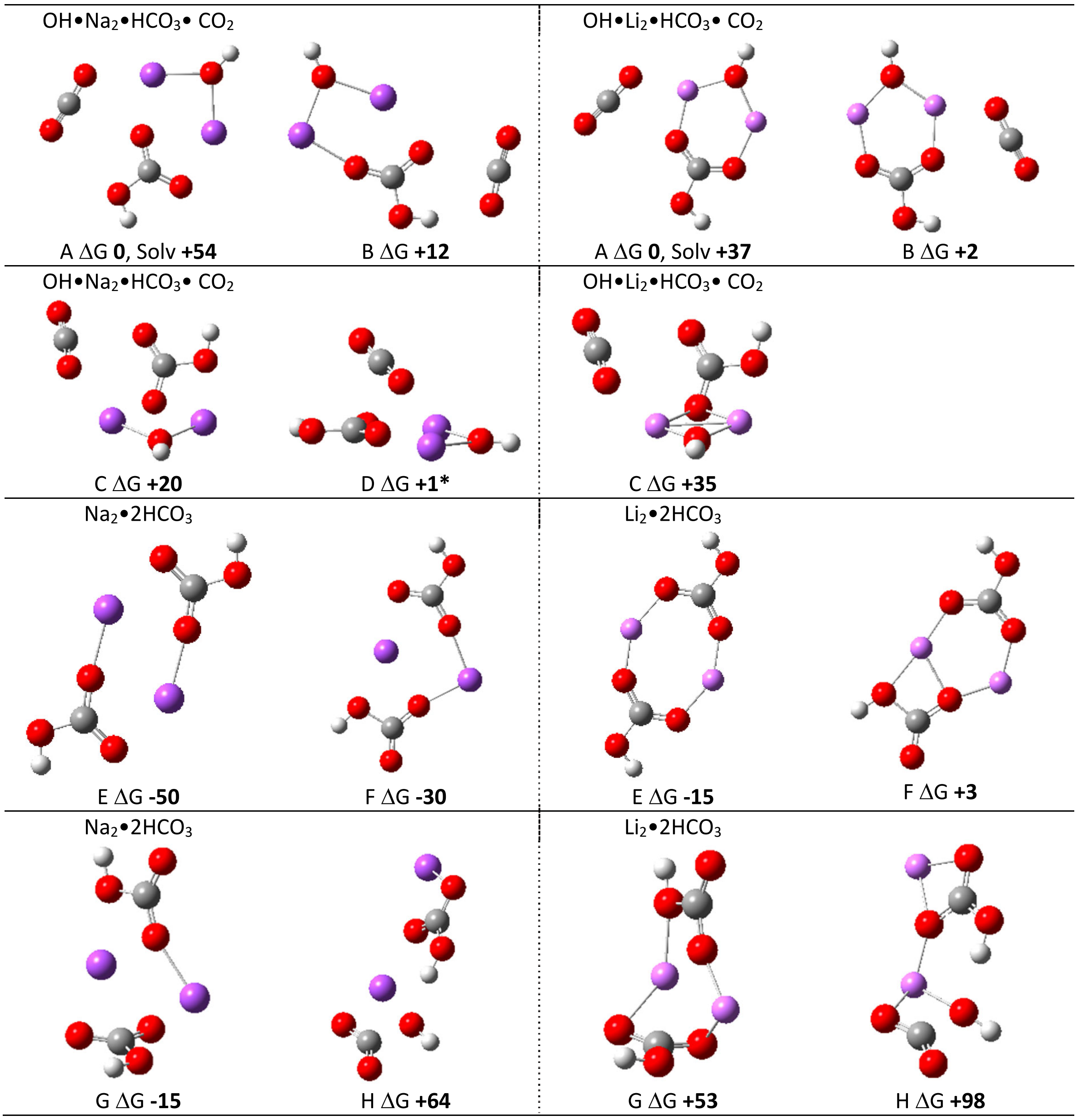

Figure. S24. Conformers A': $HO{\bullet}M_n{\bullet}HCO_3{\bullet}CO_2$ Mn where M=Li or Na and n = 2. ΔG relative Gibbs free energies in the gas-phase to the $OH{\bullet}M_2{\bullet}HCO_3{\bullet}CO_2$ conformer in the gas phase. Solv. are solvation Gibbs free energies in the melt. Both Gibbs free energies in kJ mol-1 at 25 ºC. * is only solvent stable, gas-phase goes to conformer A.

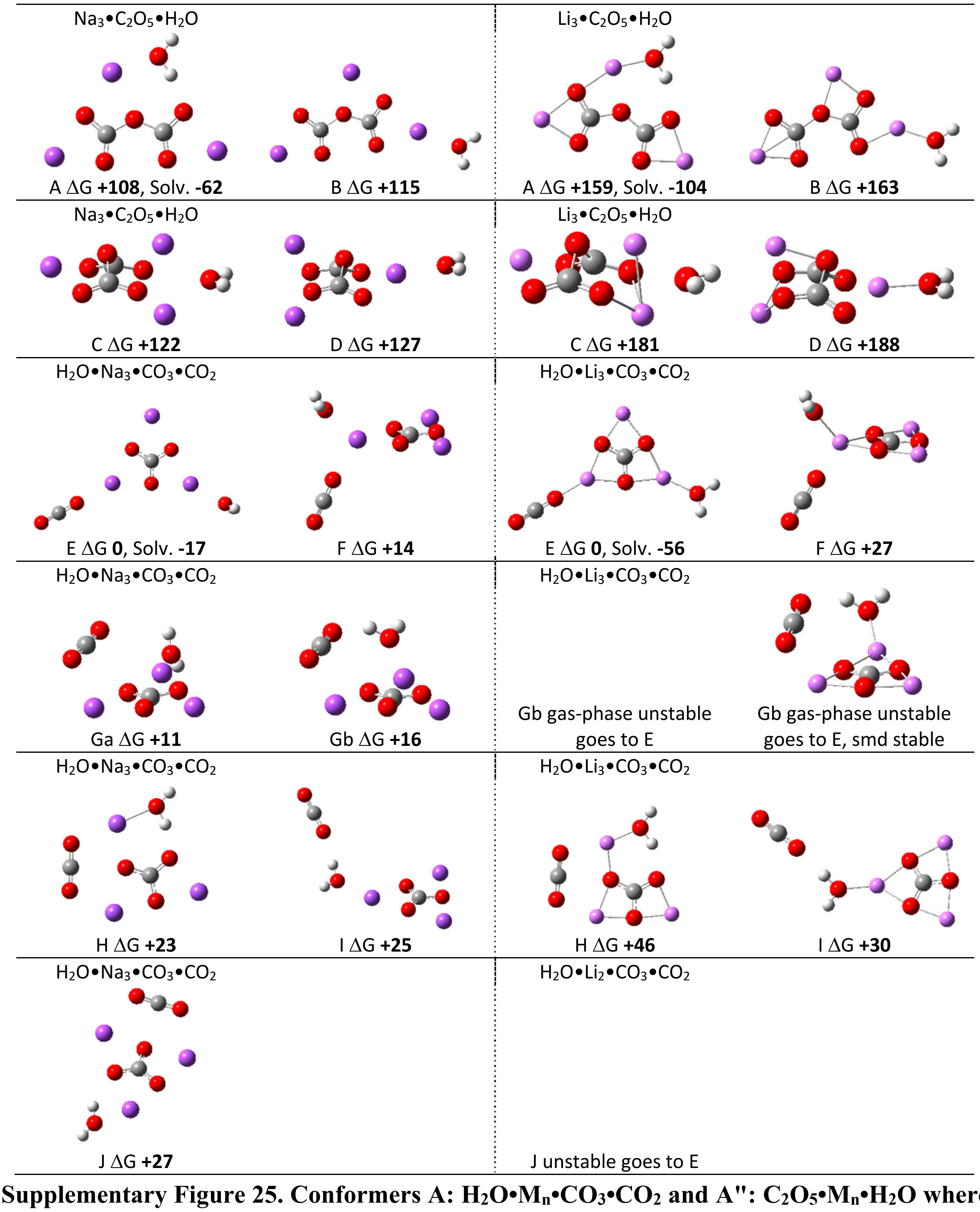

Supplementary Figure 25. Conformers A: H_2O•M_n•CO_3•CO_2 and A": C_2O_5•M_n•H_2O where M=Li or Na and n = 3. ΔG relative Gibbs free energies in the gas-phase to the lowest energy H_2O•M_3•CO_3•CO_2 conformer. Solv. are solvation Gibbs free energies in the melt. Both Gibbs free energies in kJ mol^{-1} at 25 ºC.

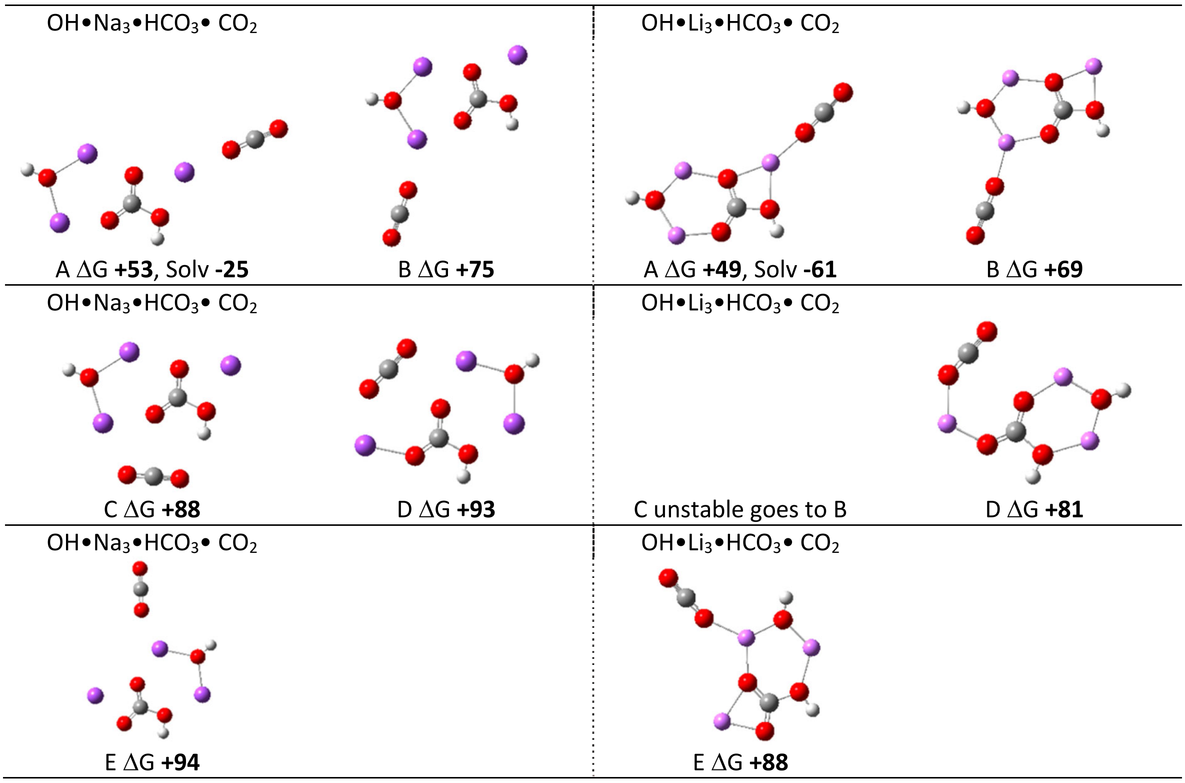

Supplementary Figure 26. Conformers A': $OH•M_n•HCO_3•CO_2$, M_n where M=Li or Na and n = 3. ΔG relative Gibbs free energies in the gas-phase to the lowest energy $H_2O•M_3•CO_3•CO_2$ conformer. Solv. are solvation Gibbs free energies in the melt. Both Gibbs free energies in kJ mol^{-1} at 25 °C.

<u>Species C_{CO_2} and C'_{CO_2}, conformers based on M_n-CO_3-CO_2 (M=Li, Na, n=1,2,3)</u>

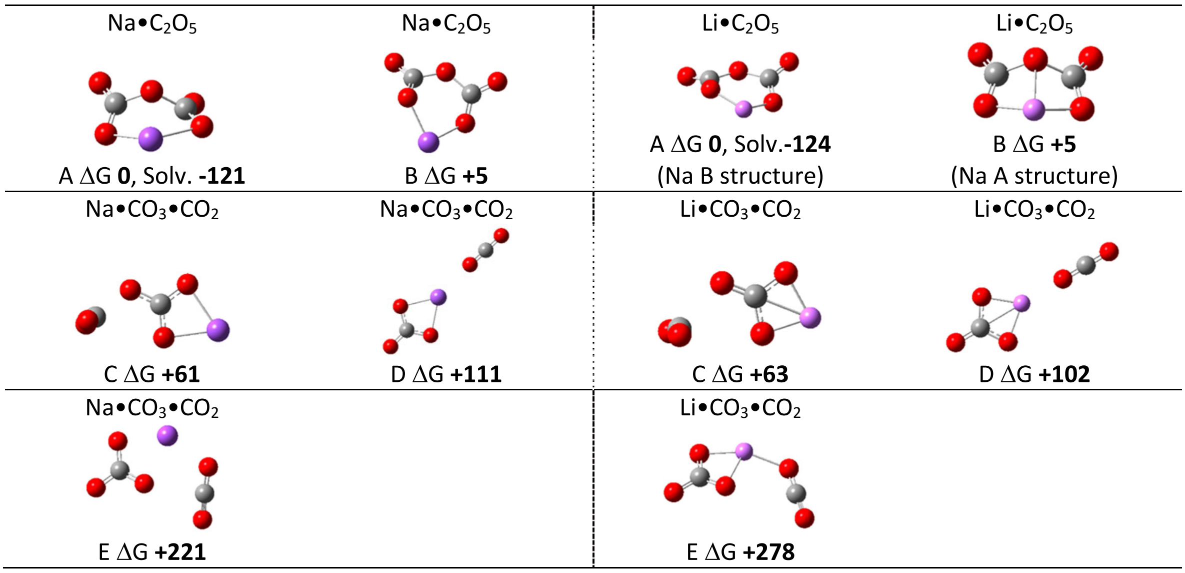

Supplementary Figure 27. Conformers C_{CO2} and C'_{CO2}: $M_n•CO_3•CO_2$ and B': $M_n•C_2O_5$ and related where M=Li or Na and n = 1. ΔG relative Gibbs free energies in the gas-phase to the

lowest energy gas-phase conformer. Solv. are solvation Gibbs free energies in the melt. Both Gibbs free energies in kJ mol^{-1} at 25 ºC.

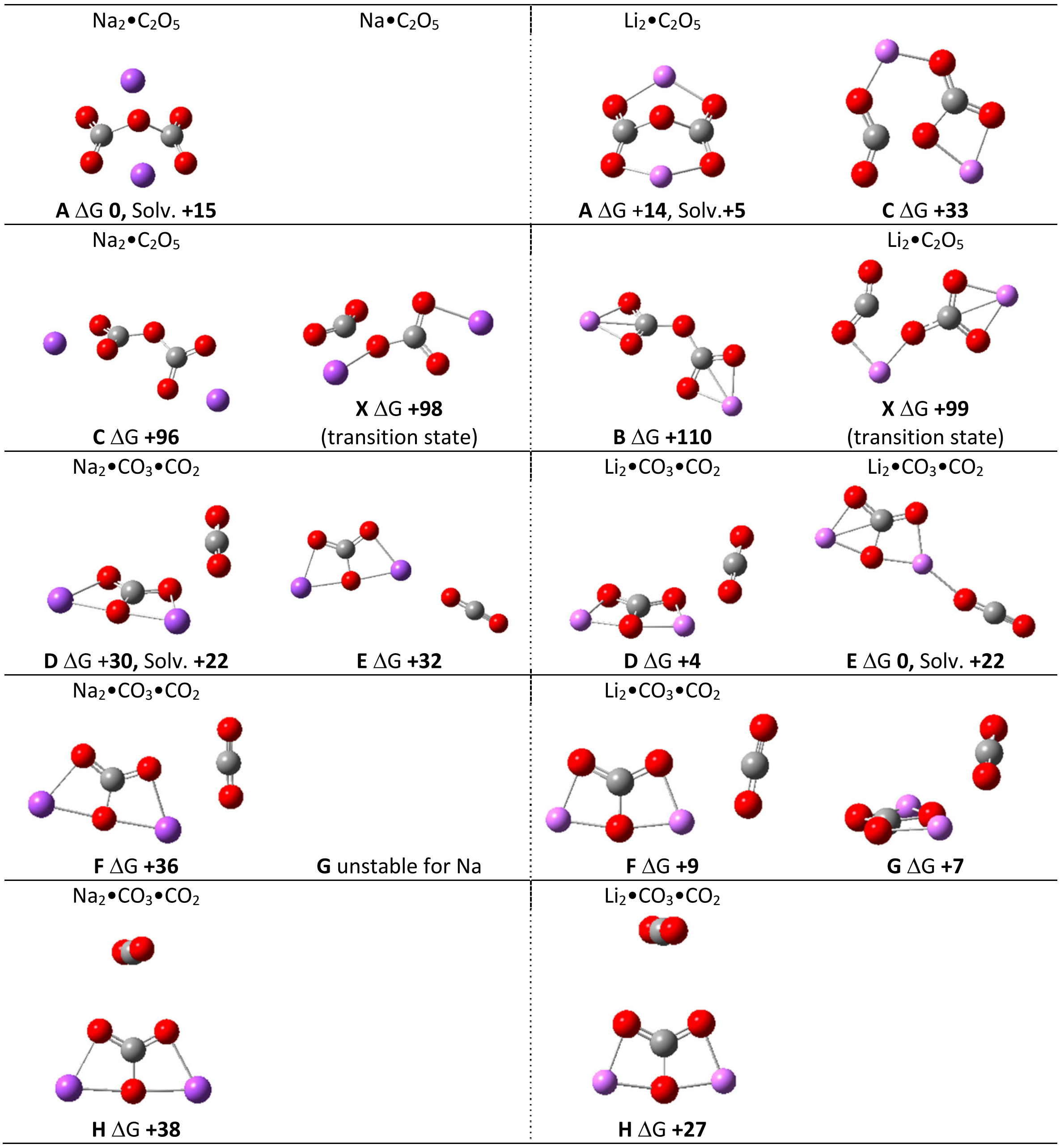

Supplementary Figure 28. Conformers C_{CO2}: M_n•CO_3•CO_2 and and C'_{CO2}: M_n•C_2O_5 and related where M=Li or Na and n = 2. ΔG relative Gibbs free energies in the gas-phase to the lowest energy (similar) conformer. Solv. are solvation Gibbs free energies in the melt. Both Gibbs free energies in kJ mol^{-1} at 25 ºC.

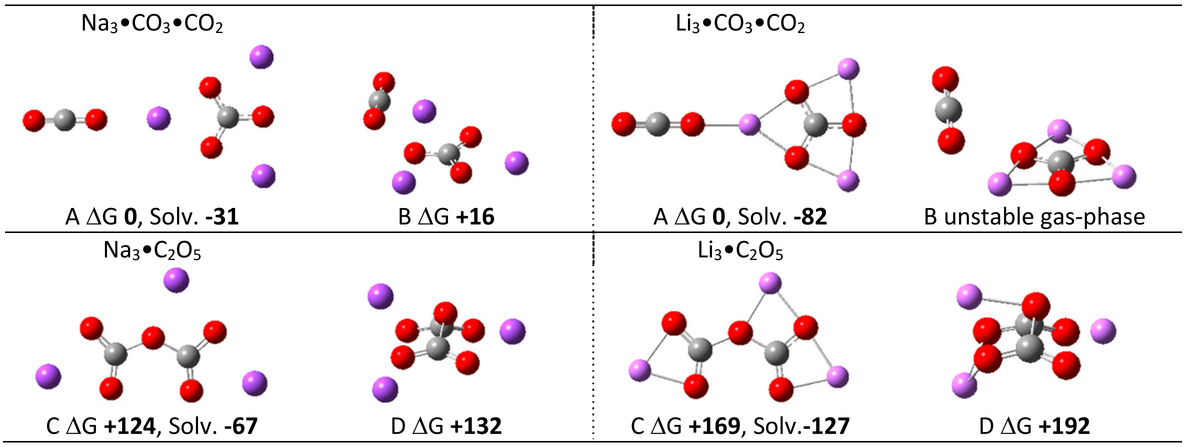

Supplementary Figure 29. Conformers C_{CO2}: M_n•CO_3•CO_2 and C'_{CO2}: M_n•C_2O_5 and related where M=Li or Na and n = 3. ΔG relative Gibbs free energies in the gas-phase to the lowest energy conformer. Solv. are solvation Gibbs free energies in the melt. Both Gibbs free energies in kJ mol^{-1} at 25 ºC.

Species C_{H_2O} and C'_{H_2O} composed of H_2O-M_n-CO_3

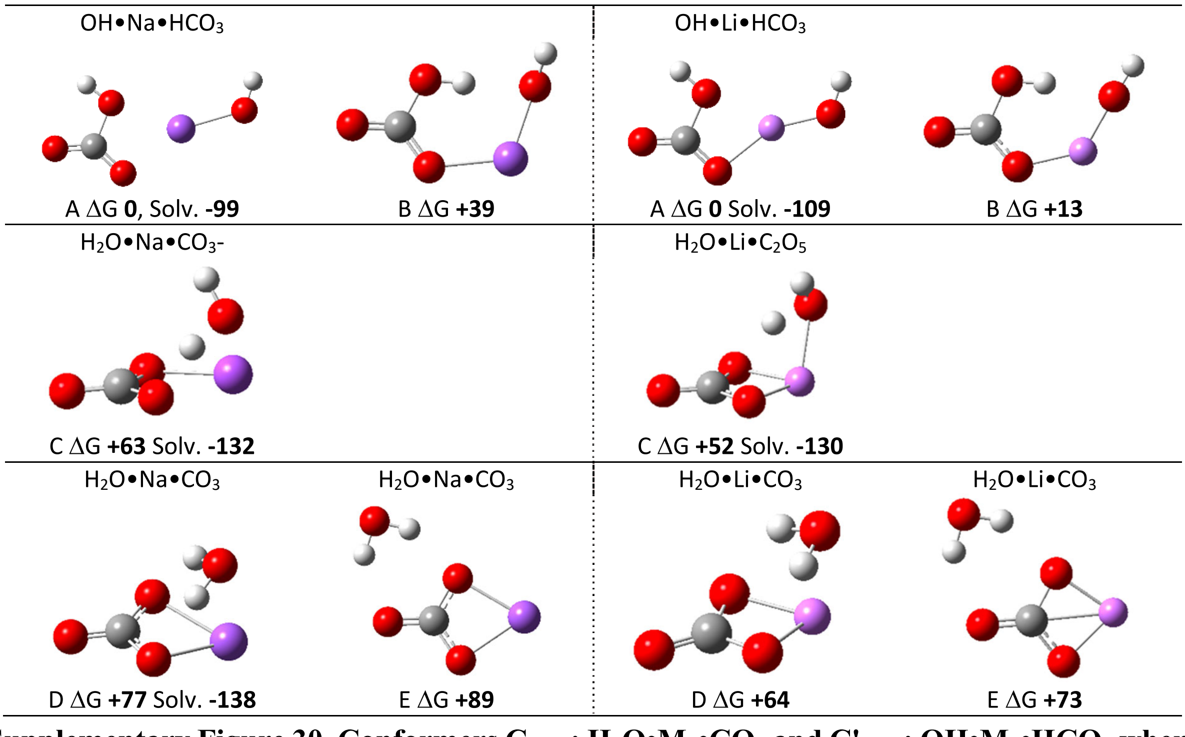

Supplementary Figure 30. Conformers C_{H2O}: H_2O•M_n•CO_3 and C'_{H2O}: OH•M_n•HCO_3 where M=Li or Na and n = 1. ΔG relative Gibbs free energies in the gas-phase to the lowest energy conformer. Solv. are solvation Gibbs free energies in the melt. Both Gibbs free energies in kJ mol^{-1} at 25 ºC.

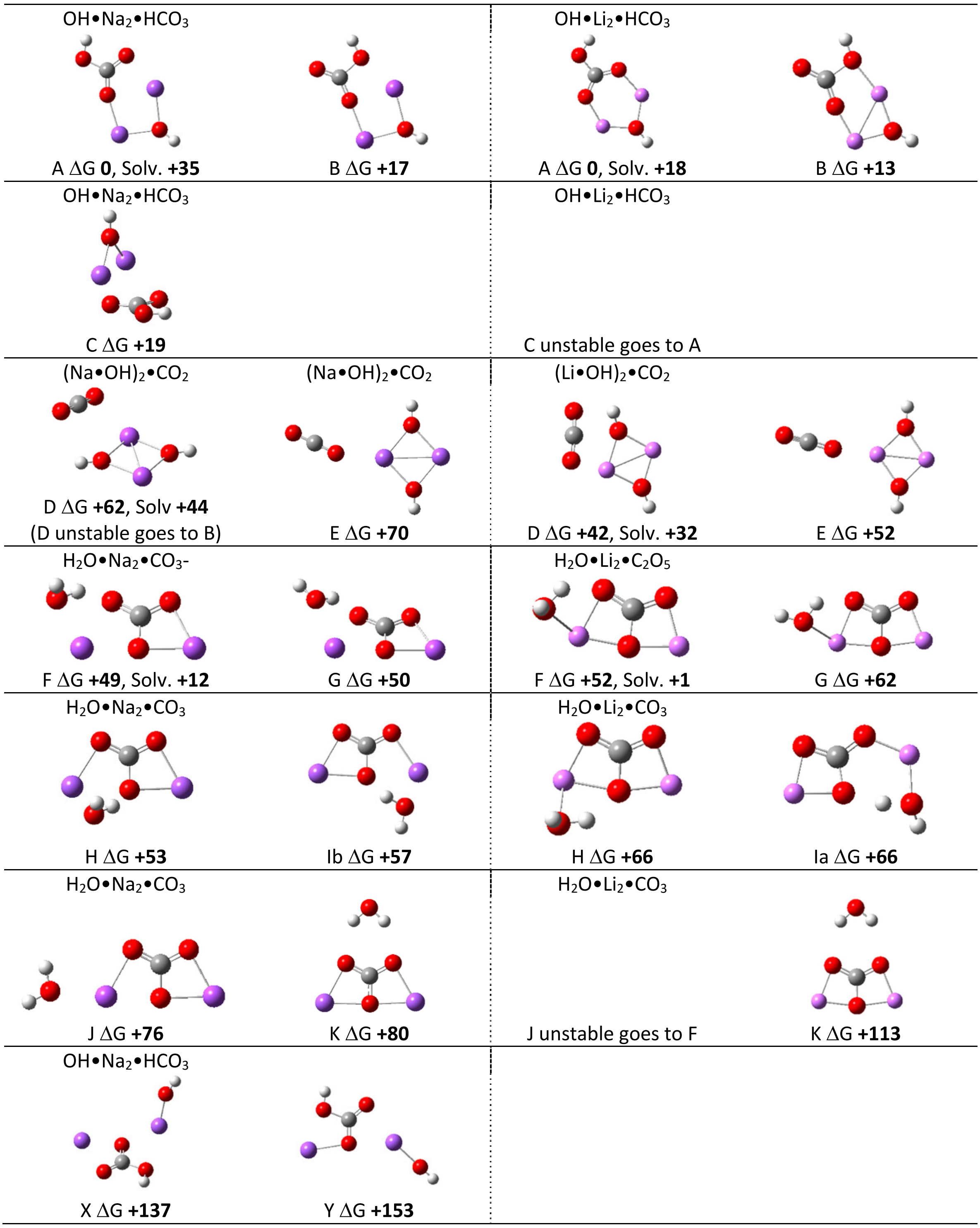

Supplementary Figure 31. Conformers C_{H2O}: H_2O•M_n•CO_3 and C'_{H2O}: OH•M_n•HCO_3 where M=Li or Na and n = 2. ΔG relative Gibbs free energies in the gas-phase to the lowest energy conformer. Solv. are solvation Gibbs free energies in the melt. Both Gibbs free energies in kJ mol^{-1} at 25 °C.

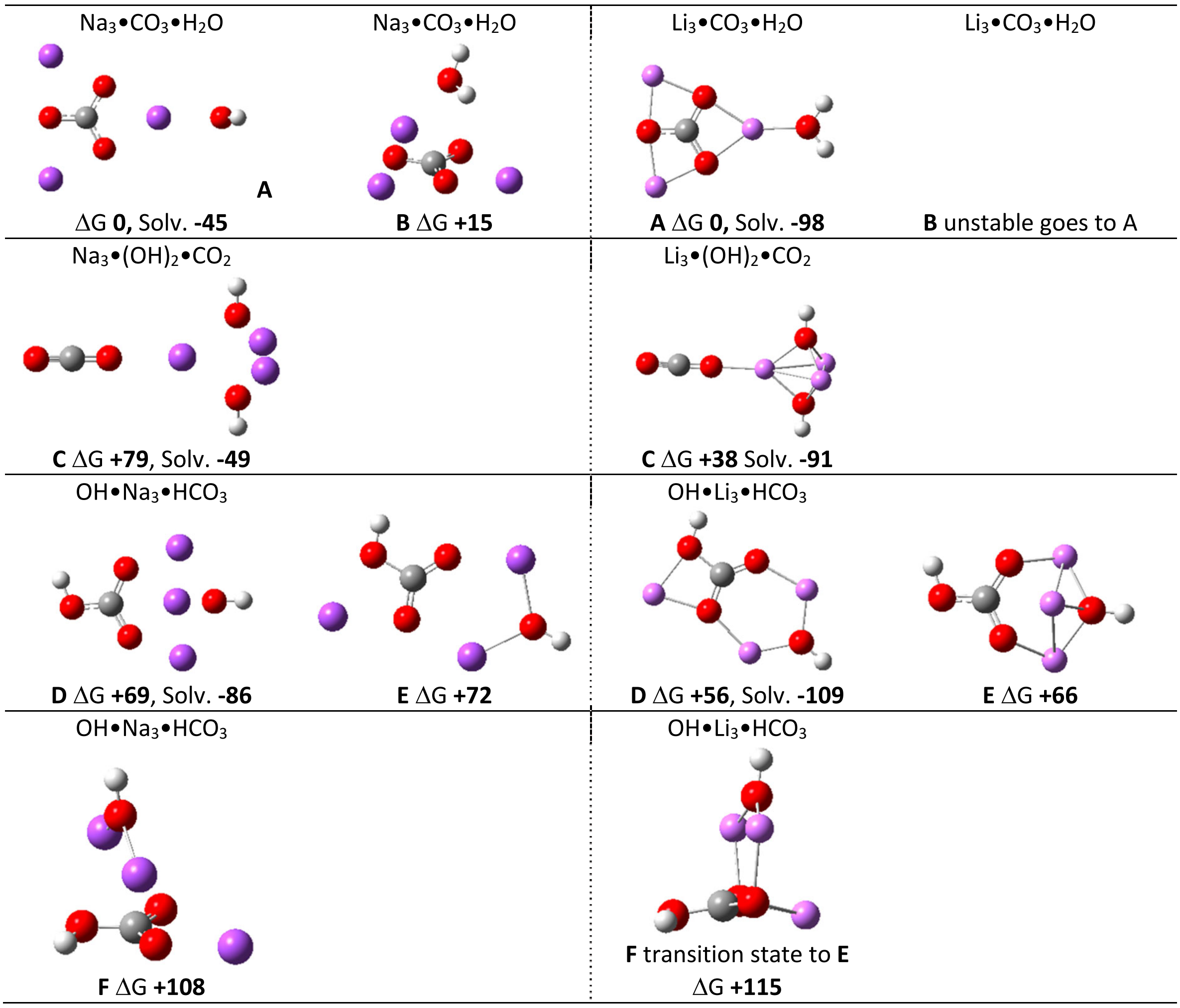

Supplementary Figure 32. Conformers C_{H2O}: H_2O•M_n•CO_3 and C'_{H2O}: OH•M_n•HCO_3 where M=Li or Na and n = 3. ΔG relative Gibbs free energies in the gas-phase to the lowest energy conformer. Solv. are solvation Gibbs free energies in the gas-phase. Both Gibbs free energies in kJ mol^{-1} at 25 ºC.

Supplementary References

1. Lin, J.Y.S., Ovalle-Encinia, O., Dual-phase ionic-conducting membranes: Pressure dependence of gas permeation flux. *J. Memb. Sci. Lett.* **3**, 100041 (2023).
2. Comesana-Gandara, B. *et al.* Redefining the Robeson upper bounds for CO_2/CH_4 and CO_2/N_2 separations using a series of ultrapermeable benzotriptycene-based Polymers of Intrinsic Microporosity. *Energy Environ. Sci.* **12**, 2733–2740 (2019).
3. Mutch, G. A. *et al.* Supported molten-salt membranes for carbon dioxide permeation. *J. Mater. Chem. A* **7**, 12951–12973 (2019).
4. Tsochataridou, S. *et al.* Measuring Membrane Permeation Rates through the Optical Visualization of a Single Pore. *ACS Appl. Mater. Interfaces* **12**, 16436–16441 (2020).
5. Kazakli, M. *et al.* Controlling molten carbonate distribution in dual-phase molten-salt ceramic membranes to increase carbon dioxide permeation rates. *J. Memb. Sci.* **617**, 118640 (2021).
6. Marenich, A.V., Olson, R.M., Kelly, C.P., Cramer, C.J., Truhlar, D.G., Self-Consistent Reaction Field Model for Aqueous and Nonaqueous Solutions Based on Accurate Polarized Partial Charges. *J. Chem. Theory Comput.* **3**, 2011–2033 (2007).
7. Marenich, A.V., Cramer, C.J., Truhlar, D.G., Universal Solvation Model Based on Solute Electron Density and on a Continuum Model of the Solvent Defined by the Bulk Dielectric Constant and Atomic Surface Tensions. *J. Phys. Chem. B.* **113**, 6378–6396 (2009).
8. Bernales, V.S., Marenich, A.V., Contreras, R., Cramer, C.J., Truhlar, D.G., Quantum Mechanical Continuum Solvation Models for Ionic Liquids. *J. Phys. Chem. B.* **116**, 9122–9129 (2012).
9. Bryantsev, V.S., Diallo, M.S., Goddard, W.A., Calculation of Solvation Free Energies of Charged Solutes Using Mixed Cluster/Continuum Models. *J. Phys. Chem. B.* **112**, 9709–9719 (2008).
10. White, S.H., Twardoch, U.M., The Behavior of Water in Molten Salts. *J. Electrochem. Soc.* **134**, 1080 (1987).
11. Frangini, S., Felici, C., Tarquini, P., A Novel Process for Solar Hydrogen Production Based on Water Electrolysis in Alkali Molten Carbonates. *ECS Trans.* **61**, 13 (2014).
12. Xiong, X., Lei, X., Zhang, C., Wang, J., Huang, K., Synergetic proton conduction in $BaZr_{0.8}Y_{0.2}O_{3-\delta}$-carbonate composite electrolyte for intermediate-temperature solid oxide fuel cells. *Solid State Ion.* **279**, 66–71 (2015).
13. Gürbüz, E., Grépin, E., Ringuedé, A., Lair, V., Cassir, M., Significance of Molten Hydroxides With or Without Molten Carbonates in High-Temperature Electrochemical Devices. *Front. Energy Res.* **9**, 666165 (2021).
